# School-level self-reported versus objective measurements of body mass index in public high school students

**DOI:** 10.1016/j.ypmed.2023.107616

**Published:** 2023-07-13

**Authors:** Hannah R. Thompson, Kristine A. Madsen, Caroline Nguyen, Kira Argenio, Emily D’Agostino, Kevin Konty, Sophia Day

**Affiliations:** a University of California Berkeley, School of Public Health, Community Health Sciences, 2121 Berkeley Way West, Berkeley, CA 94720, United States; b Duke University School of Medicine, Department of Population Health Sciences, Department of Family Medicine & Community Health, 311 Trent Drive, Durham, NC 27710, United States; c NYC Department of Health and Mental Hygiene, Office of School Health, 125 Worth St, New York, NY 10013, United States

**Keywords:** Body mass index (BMI), Adolescent health, Weight status, School health, Population health, Obesity

## Abstract

Population-level surveillance of student weight status (particularly monitoring students with a body mass index (BMI) ≥95th percentile) remains of public health interest. However, there is mounting concern about objectively measuring student BMI in schools. Using data from the nation’s largest school district, we determined how closely students’ self-reported BMI approximates objectively-measured BMI, aggregated at the school level, to inform decision-making related to school BMI measurement practices. Using non-matched data from *n* = 82,543 students with objective height/weight data and *n* = 7676 with self-reported height/weight from 84 New York City high schools (88% non-white and 75% free or reduced-price meal-eligible enrollment), we compared school-level mean differences in height, weight, BMI, and proportion of students by weight status, between objective and self-reported measures. At the school-level, the self-reported measurement significantly underestimated weight (−1.38 kg; 95% CI: −1.999, −0.758) and BMI (−0.38 kg/m^2^; 95% CI: −0.574, −0.183) compared to the objective measurement. Based on the objective measurement, 12.1% of students were classified as having obesity and 6.3% as having severe obesity (per CDC definition); the self-report data yielded 2.5 (95% CI: −1.964, −0.174) and 1.4 (95% CI: −2.176, −0.595) percentage point underestimates in students with obesity and severe obesity, respectively. This translates to 13% of students with obesity and 21% of students with severe obesity being misclassified if using self-reported BMI. School-level high school students’ self-reported data underestimate the prevalence of students with obesity and severe obesity and is particularly poor at identifying highest-risk students based on BMI percentile.

## 1. Introduction

Monitoring youths’ weight status at the population level continues to be a priority for many public health practitioners and policy makers in the United States (US), with school-based student body mass index (BMI) measurements constituting the largest source of these data in most states (Blondin et al., 2016). As of 2014, approximately 40% of US public and private schools reported screening students’ weight status using BMI (Centers for Disease Control and Prevention, 2015), the practice of which has been supported by the Institute of Medicine, the American Public Health Association, and the American Heart Association (Committee on Accelerating Progress in Obesity Prevention et al., 2012; Association AH, 2008; Association APH, 2002).

However, there is mounting concern about measuring student BMI in the school setting, with critics raising questions about the lack of safeguards in place for students and the unintended consequences (like increased weight stigma) associated with the practice (Sliwa et al., 2019; Madsen et al., 2021). As such, some of the nation’s largest Departments of Education have paused collection of objective BMI data in schools (e. g. California Department of Education, n.d.; Lee, 2020) or are working to determine the feasibility of doing so, such as the New York City Department of Education (NYCDOE).

Despite concerns about BMI screening in schools, population-level surveillance of student weight status remains of interest to many public health interest groups. In particular, monitoring the school-level proportion of students with a BMI ≥95th percentile for sex and age (students who are presumably at greatest cardiometabolic risk; Harrington et al., 2013), and specifically students classified as having severe obesity (≥120% of the 95th percentile or a BMI ≥35), students at the highest increased cardiometabolic risk (Skinner et al., 2015), is of interest. This monitoring is intended to help inform the design, implementation, and targeting of effective interventions to address student health, as well as to address related racial/ethnic health disparities (Konty et al., 2022; Patel et al., 2021). If assessing weight status remains a priority, student self-reported BMI, which would likely be more comfortable for many students (particularly females and those with a BMI ≥ 95th percentile) (Madsen et al., 2021; Kalich et al., 2008), might serve as a replacement for objectively-measured BMI.

While objectively measured and self-reported BMI are typically highly correlated, self-reported BMI has been shown to be less accurate, with a systematic bias towards underreported weight and overreported height (Wen and Kowaleski-Jones, 2012). For example, recent data from the National Health and Examination Survey, showed misclassification of adult participants with underweight and obesity ranged between 30 and 40% when using self-reported data (Keith et al., 2011). Further, differences between self-reported and measured BMI are known to differ by sex, age, race/ethnicity, and weight status, with females and those with overweight or obesity more likely to underreport weight compared to males and students with healthy weight, respectively (Yoshitake et al., 2012; Allison et al., 2020; Pérez et al., 2015). In teenagers, specifically, self-reported height and weight data have been shown to result in a lower estimate of the prevalence of overweight and obesity (Crawley and Portides, 1995). A 2007 review examining the accuracy of adolescents’ self-reported height and weight (Sherry et al., 2007) called for stronger evidence about subpopulation differences, a gap which remains to be filled.

As large Departments of Education like NYCDOE consider the feasibility of using students’ self-reported BMI as a replacement for objectively measured BMI, an important initial step is to identify how closely school-level self-reported BMI can estimate objectively-measured BMI across demographic groups. We utilize data from NYCDOE, the largest and among the most socio-economically and racially/ethnically diverse school districts in the country (Chen, 2018). Using both self-reported BMI (collected anonymously at the individual level) and objectively measured BMI (collected at the individual level), we sought to determine how closely high school students’ self-reported BMI can approximate students’ objectively-measured BMI, aggregated at the school-level. These data, collected in the real-world public-school setting, were compared overall, as well as stratified by key-characteristics known to be associated with height and weight over- and under-reporting (sex, age, and race/ethnicity). Results from this study are intended to inform decision-making related to school-based BMI measurement practices.

## 2. Methods

### 2.1. Data sources and study population

Data for this study were drawn from the New York City Youth Risk Behavior Survey (NYCYRBS) and NYC FITNESSGRAM^®^ (Konty et al., 2020; Day et al., 2014) datasets jointly managed by NYCDOE and NYC Department of Health and Mental Hygiene (NYCDOHMH) for the 2017–18 school year (the most recent year for which complete data are available from both sources).

As part of the FITNESSGRAM^®^, NYCDOE staff are required to conduct height and weight assessments annually for all students in grades 9–12 (NYC Department of Education, n.d.). NYCDOE schools are required to have ≥85% of eligible students complete the FITNESSGRAM^®^ assessment annually. The NYCYRBS is conducted by NYCDOHMH in collaboration with the NYCDOE as part of the National Centers for Disease Control and Prevention’s (CDC) Youth Risk Behavior Surveillance System (YRBSS) (Centers for Disease C, Prevention, and Brener, 2013). The sampling frame for the 2017–18 NYCYRBS constituted 431 public high schools serving NYC students, grades 9–12. From this frame, NYCDOHMH selected a sample of 99 schools to be representative of each borough (and the city, overall), among which 92 schools participated. Within participating schools, a sample of students was anonymously surveyed and their response data were statistically weighted to be representative of all students included in the sampling frame (New York City Department of Health and Mental Hygiene Bureau of Epidemiology Services Surveys and Data Analysis Unit, 2021). Because NYCYRBS data are collected without student identifiers to protect anonymity, they cannot be directly linked to individual student FITNESSGRAM^®^ results. Prior work has examined individually-collected and identified FITNESSGRAM^®^ data in relation to individually-collected but anonymous YRBS data in this population (Day et al., 2016).

The present study sample included 84 of the 92 high schools administering the 2017–18 NYCYRBS that also conducted the FITNESSGRAM^®^ that year, with total enrollment of 91,513 students. Among the 84 schools in the sample, FITNESSGRAM^®^ height and weight data were available for 82,543 students (90%; range across schools 24% to 100%) and YRBS height and weight data for 7676 students (8%; range across schools 2% to 61%). Both the NYCDOHMH Institutional Review Board and the UC Berkeley Committee for the Protection of Human Subjects deemed this non-human subject research.

### 2.2. NYCYRBS measures

Students selected to participate in the NYCYRBS completed an anonymous questionnaire during the school day. Students self-reported their age (in whole number); grade (9–12); sex (male or female); height (in feet and inches); weight (in pounds); and race/ethnicity (Hispanic/Latino, and non-Hispanic American Indian/Alaska Native, Asian, Black/African American, Native Hawaiian/Other Pacific Islander, White, and Multiple Races).

### 2.3. FITNESSGRAM^®^ measures

The FITNESSGRAM^®^ was conducted by physical education teachers with formal training on administering the test, including manuals, video-based training, and site visits, as well as standardized equipment (Konty et al., 2022; NYC Department of Education, n.d.). Height and weight were most frequently assessed using the Health-O-Meter 500 KL combined scale and stadiometer. Per protocol (Plowman and Meredith, 2013), height was measured twice to the nearest 0.1 in.; if the two measures were off by >0.5 in., a third height was recorded. Weight was measured to the nearest integer pound. These data were linked with individual student school records containing student birthdate (used to calculate age in whole numbers, rounded down to the nearest whole number); grade (9–12); gender (male or female); and race/ethnicity, as reported by the student’s parent/guardian (which was collapsed to match the seven YRBS race/ethnicity categories, to allow for comparisons between datasets).

### 2.4. BMI and weight status classification

BMI was calculated as weight in kilograms/height in meters (Konty et al., 2022). Student age, together with student sex, height (converted to meters), and weight (converted to kilograms), were converted to age- and sex-specific BMI percentile using the 2022 Center for Disease Control and Provention (CDC) clinical growth charts (Centers for Disease Control and Prevention, 2022). Measures resulting from the YRBS dataset were classified as self-reported; measures from the FITNESSGRAM^®^ dataset were classified as objectively measured.

For all measures, age- and sex-specific criteria were used to identify biologically implausible values; if a student was flagged as having at least one biologically implausible value, the individual’s height, weight, and BMI data were set to missing (0.02% of observations for FITNESSGRAM^®^ and 0.05% of observations for NYCYRBS) (Konty et al., 2022). As defined by the CDC (n.d.), students’ weight status was classified as: underweight (BMI < 5th percentile); healthy weight (BMI ≥ 5th and < 85th percentile); overweight (BMI ≥ 85th and < 95th percentile); obesity (BMI ≥ 95th percentile); or severe obesity (≥120% of the 95th percentile *or* a BMI ≥ 35).

### 2.5. School level characteristics

Publicly available school-level data were downloaded from the NYCDOE data website, including total school enrollment and proportion of students eligible for free or reduced-price meals (FRPM: a proxy for low socioeconomic status) (New York City Department of Ecuation Info Hub, n.d.). The proportion of NYCYRBS to FITNESSGRAM^®^ participants was calculated as the number of students who completed the NYCYRBS divided by the number of students who completed the FITNESSGRAM^®^ at each school.

### 2.6. Statistical analysis

Descriptive statistics were used to examine differences between the FITNESSGRAM^®^ and NYCYRBS samples. Because the anonymity of the NYCYRBS data precluded linking individual student data, data were aggregated at the school level for height, weight, BMI, and the proportion of students classified by weight status. School-level mean differences were calculated for all students, and for student groups stratified by grade, sex, and race/ethnicity, as the NYCYRBS school mean less the FITNESSGRAM^®^ school mean; negative differences suggest that students under-reported their true height or weight. Bland-Altman (B-A) plots compared the mean differences between school-level NYCYRBS and FITNESSGRAM^®^ measurements against the averages of the two measurements (Bland and Altman, 1986). B-A plots enable the visual assessment of measurement agreement and bias (present when the line of equality -zero difference- falls outside the limits of agreement (LOA), which are calculated as the mean of the two measurements ±1.96 SDs). Mean differences in height, weight, BMI, and the proportion of students in BMI weight status categories were also calculated using paired *t*-tests to provide 95% confidence intervals for the sample mean differences. Adjusted linear regression models were used to determine if school-level characteristics predicted mean differences. Analyses were conducted in Stata/MP 16.1 (StataCorp, College Station, Texas).

## 3. Results

Demographic characteristics of the students (*n* = 82,543 students with FITNESSGRAM^®^ data; *n* = 7676 with NYCYRBS data) from the 84 schools are described in Table 1. There were statistically significant differences (*p* < 0.01) in sex, age, grade, and race/ethnicity between the FITNESSGRAM^®^ and NYCYRBS samples. Across schools, the mean proportion of NYCYRBS participants to FITNESGRAM^®^ participants was 18% (SD ± 18%).

Across sample schools, average total enrollment was 1089 (SD ± 1143) students, with 48% (SD ± 16%) female; 12% (SD ± 15%) Asian; 30% (SD ± 22%) African American; 42% (SD ± 22%) Hispanic/Latino; and 12% (SD ± 18%) White student enrollment. On average, 75% (SD ± 16%) of students qualified for FRPM.

Table 2 presents the mean differences between NYCYRBS and FITNESSGRAM^®^ data for height, weight, and BMI for all students, as well as stratified by student sex, age, and race/ethnicity. Overall, the mean difference in height measurements was negligible (−0.003 m; 95% CI: −0.008, 0.001) and not significant. The mean difference in weight was −1.38 kg (95% CI: −1.999, −0.758) and the mean difference in BMI was −0.38 kg/m^2^ (95% CI: −0.574, −0.183), with the NYCYRBS data underestimating the FITNESSGRAM^®^ data in both cases. B-A plots, to assist in the visualization of differences between measurements, can be found in the Appendix file.

For females, the average NYCYRBS significantly underestimated weight (difference = −2.16 kg; 95% CI: −2.890, −1.438) and BMI (difference = −0.56 kg/m^2^; 95% CI: −0.857, −0.272) compared to the FITNESSGRAM^®^ measurement. However, for males, the NYCYRBS overestimated height (difference = 0.01 m; 95% CI: 0.002, 0.012), but was not significantly different for weight or BMI. For younger students, the NYCYRBS underestimated compared to the FITNESSGRAM^®^ measurement for weight (age 13 difference = −3.34 kg, 95% CI: −6.127, −0.563; age 15 difference = −1.63 kg, 95% CI: −2.550, −0.707; age 16 difference = −1.18 kg, 95% CI: −2.229, −0.122), which led to under-reporting for NYCYRBS BMI measurements. For older students (ages 17 and 18), there were no significant differences between NYCYRBS and FITNESSGRAM^®^ measurements of height, weight, or BMI. When looking within racial/ethnic groups, among American Indian/Alaskan Native and Hispanic/Latino students, the NYCYRBS weight measurement was underestimated (difference = −4.54 kg, 95% CI: −8.227, −0.850 and difference = −1.56 kg, 95% CI: −2.476, −0.636, respectively), which contributed to significant underestimation of BMI (difference = −1.81 kg/m^2^, 95% CI: −3.019, −0.606 and difference = −0.43 kg/m^2^, 95% CI: −0.711, −0.158 respectively).

**Fig. 1.**
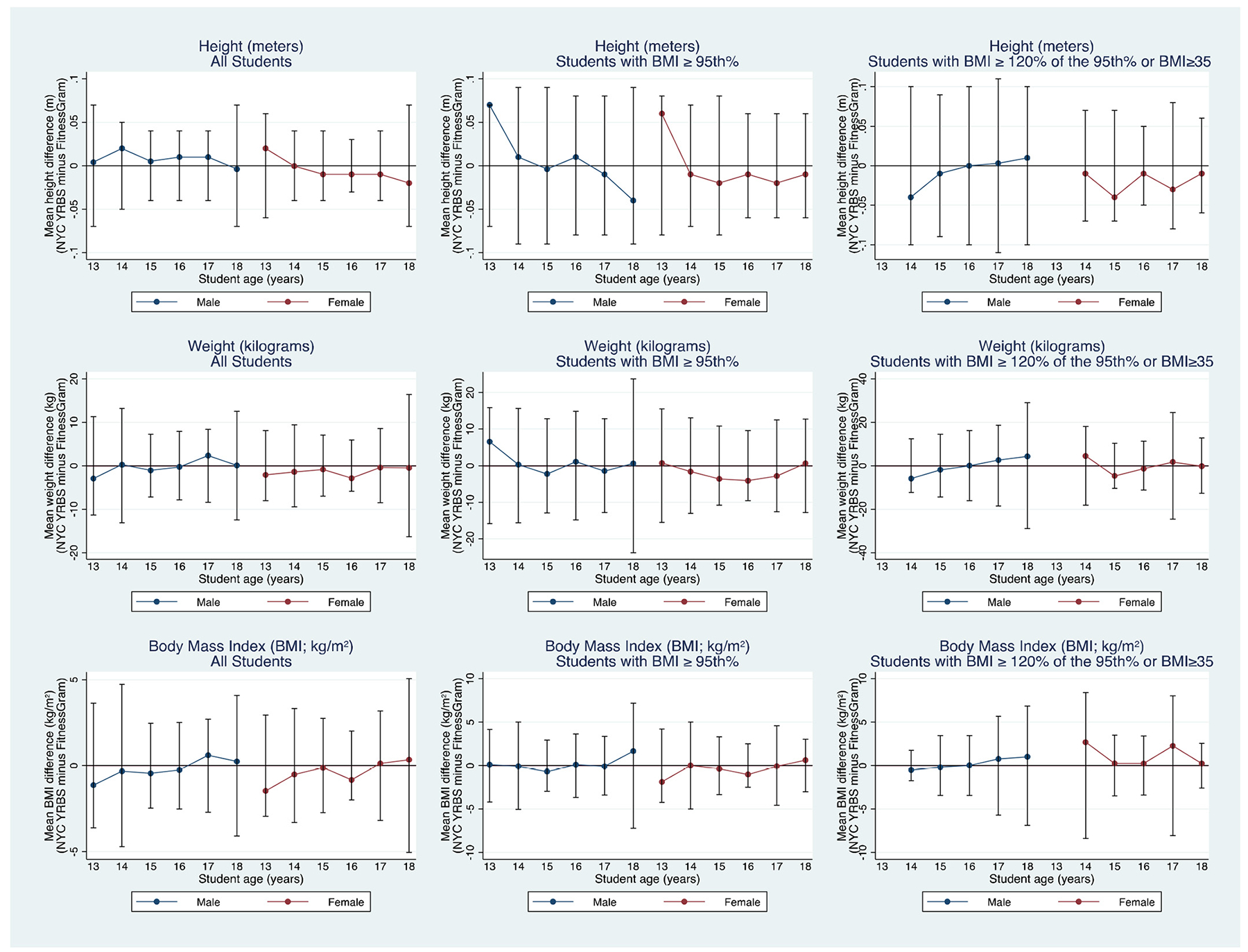
School-level mean measurement differences (and standard deviations) for height, weight, and Body Mass Index (BM) by measurement type (FITNESSGRAM^®^ and New York City Youth Risk Behavior Survey (NYCYRBS)), stratified by student sex and age (*N* = 84 schools).

Fig. 1 displays school-level mean differences between the NYCYRBS and FITNESSGRAM^®^ measurements for height, weight, and BMI for students stratified by both sex and age for all students, as well as for students with obesity and severe obesity (these data can be found in table format in the Appendix). Examining data for all students, the YRBS measurement underestimated BMI for females at ages 13 (−1.46 kg/m^2^ m, 95% CI: −2.489, −0.433) and 16 (−0.83 kg/m^2^, 95% CI: −1.279, −0.390) compared to the FITNESSGRAM^®^ measurement, but there were no statistically significant differences in BMI for other female ages or for males at any age (Appendix Table 1). There were only statistically significant differences in the school-level mean NYCYRBS and FITNESSGRAM^®^ measurements for females age 16 (−1.00 kg/m^2^, 95% CI: −1.703, −0.295) with obesity (Appendix Table 2), but there were no other differences by age and sex for students with obesity or severe obesity (Appendix Table 3).

Table 3 displays the mean school-level differences in proportion of students by weight status category between measurements (B-A plots can be found in the Appendix file). Based on the FITNESSGRAM^®^ measurement, 18.4% of students were classified as having obesity. The NYCYRBS self-report data yielded a 2.5 percentage point underestimate (95% CI: −3.776, −1.132) in students with obesity and a 1.4 percentage point underestimate of students with severe obesity (95% CI: −2.151, −0.569). This translates to 13% of students with obesity and 21% of students with severe obesity being misclassified if using self-reported BMI.

Based on the FITNESSGRAM^®^ measurement, 2.6% of students were classified as having underweight, 60.6% as having healthy weight, and 18.4% as having overweight. Overall, the NYCYRBS overestimated the proportion of students with underweight (difference = 0.6%, 95% CI: 0.069, 1.115) and healthy weight (difference = 2.3%, 95% CI: 0.429, 4.084).

The NYCYRBS underestimated the proportion of students with obesity and severe obesity for several groups of students, based on demographic characteristics (Table 3). The NYCYRBS underestimated the proportion of students with obesity for: females (difference = −3.7%, 95% CI: −5.443, −1.850); age 13 students (difference = −9.2%, 95% CI: −15.354, −2.942); age 14 students (difference = −5.9%, 95% CI: −9.332, −2.371); age 15 students (difference = −3.3%, 95% CI: −6.055, −0.546); American Indian/Alaska Native students (difference = −12.8%, 95% CI: −18.875, −6.639); White students (difference = −10.7%, 95% CI: −14.868, −6.518); and for Hispanic students (difference = −3.8%, 95% CI: −5.731, −1.823) compared to the FITNESSGRAM^®^. This translates to misclassification of female (22.1%), age 13 (46.9%), age 14 (28.4%), age 15 (17.5%), American Indian/Alaska Native (76.9%), White (57.5%), and Hispanic (18.7%) students with obesity if using the self-reported measure.

The NYCYRBS underestimated the proportion of students with severe obesity for females (difference = −2.1%, 95% CI: −3.060, −1.047); Age 15 students (difference = −2.0, 95% CI: −3.458, −0.531); Age 16 students (difference = −1.7, 95% CI: −2.929, −0.492); American Indian/Alaska Native students (difference = −4.3, 95% CI: −8.236, −0.369); and Hispanic/Latino students (difference = −2.0, 95% CI: −3.063, −0.909) compared to the FITNESSGRAM^®^ (Table 3). This translates to misclassification for female (36.6%), Age 15 (29.9%), Age 16 (27.4%), American Indian/Alaska Native (76.8%), and Hispanic (29.7%) students with severe obesity if using the self-reported measure.

For every 1% increase in the proportion of students who qualified for FRPM, NYCYRBS overestimated the proportion of students with obesity by 0.1% (95% CI: 0.017, 0.193) and by 0.02% for students with severe obesity (95% CI: −0.036, 0.073), adjusting for total school enrollment and proportion of NYCYRBS to FITNESSGRAM^®^ students measured (Table 4). None of the studied school characteristics significantly predicted the difference in school-level BMI between NYCYRBS and FITNESSGRAM^®^ measurement.

## 4. Discussion

In this study we compared NYCDOE 9th–12th grade students’ self-reported versus objectively-measured height, weight, BMI, and weight status, aggregated at the school level. We found that students’ self-reported data yielded weight and BMI underestimates compared to the objective measurements. This is consistent with prior studies in adolescents examining differences between self-reported and measured BMI (Allison et al., 2020; Pérez et al., 2015; Ghosh-Dastidar et al., 2016). We also found that students’ self-report overestimated the proportion of students with underweight (0.6 percentage points) and healthy weight (2.3 percentage points) but underestimated the proportion of students with obesity (2.5 percentage points) and severe obesity (1.4 percentage points). Similar to prior research among high school students (Allison et al., 2020; Pérez et al., 2015), the overall differences appear to be largely driven by underreporting of weight for females; NYCYRBS significantly overestimated the proportion of females with healthy weight by 5 percentage points, and significantly underestimated the proportion of females with obesity and severe obesity by 3.7 and 2.1 percentage points, respectively. These results suggest that high school students’ self-reported BMI, when averaged at the school-level, is not accurate compared to objectively measured BMI, given current sampling procedures.

Many school-based BMI measurement programs are used to identify schools or areas where students are at the highest potential health risk, and then apply resources or policy changes to help address these risks (Blondin et al., 2016; Konty et al., 2022). If we believe there is value in programs that track the proportion of at-risk youth based on weight status (BMI ≥95th percentile; 18% of this NYCDOE high school sample according to the objective measurement) (Harrington et al., 2013), then our findings suggest that shifting to self-reported estimates will likely underestimate obesity prevalence by 13%. More alarming, relying on self-reported estimates would result in underestimating severe obesity prevalence (6.3% of this population) by 22%. In this sense, 1 in 5 students at the very highest potential metabolic risk (including greater risk than others for hypertension, type 2 diabetes, metabolic syndrome, nonalcoholic fatty liver disease, and atherosclerosis) (Chung and Rhie, 2021) would be misclassified relying on self-reported BMI. This is similar to what has been observed in other studies, showing high self-reported misclassification or weight status by participants with obesity (Keith et al., 2011).

In addition, differences in school-level misclassification of weight status by student race/ethnicity and age were apparent. Students who are American Indian/Alaska Native (77% with both obesity and severe obesity), White (57.5% with obesity), and Hispanic (19% with obesity and 30% with severe obesity) would be misclassified if reliant on the self-reported measure. Multiple studies have found associations between race/ethnicity and errors in self-reported height and weight, primarily in cohorts of young adults and adults, though the direction and magnitude of differences are inconsistent, and understudied in U.S. adolescent populations .(Chau et al., 2013; Richmond et al., 2015; Stommel and Schoenborn, 2009; Hodge et al., 2020). Further, while a recent meta-analysis showed that self-reported height and weight values tended to be more reliable in children over age 11 (Rios-Leyvraz et al., 2022), the present study finds that when estimating school-level prevalence of obesity and severe obesity, self-reported data from younger high school aged (13–16 years-old) students with obesity and severe obesity demonstrated significant weight- status misclassification, whereas data from older high school aged (17 and 18 years-old) students did not.

Together, these findings suggest the NYCYRBS does not accurately estimate the proportion of students at highest potential health-related risk due to their weight status. Further, relying on school-level self-reported BMI data from younger, highly racially/ethnically diverse populations may result in inaccurate differences in weight status by both age and race/ethnicity.

These findings raise significant concerns if one of the primary purposes of BMI surveillance is to inform the design and implementation of effective interventions to address students’ at the highest health risk, as well as to address related racial/ethnic health disparities (Konty et al., 2022; Patel et al., 2021). However, if we are interested in trends (i.e. change over time), using self-report data might be acceptable, assuming that the bias inherent in the measure does not change over time (e.g., in response to shifts in social norms).

Valid questions remain about the value and utility of surveilling student BMI (Sliwa et al., 2019; Madsen et al., 2021; Ikeda et al., 2006). Nevertheless, these findings do not support relying on self-reported BMI aggregated at the school level, using current practices. If schools stop objective BMI measurements, additional research is needed to develop correction equations for self-reported data to more accurately estimate measured height, weight, and BMI from their highly diverse student population (Pérez et al., 2015). Using self-reported and objective BMI data from an individually-matched sample of NYCDOE students to develop appropriate BMI correction models could be an important next step, as prior evidence shows that correction models can improve the accuracy of BMI estimates from self-report data in youth (Ghosh-Dastidar et al., 2016). This work would have the additional potential benefit of reducing the unintended consequences associated with objective BMI measurement programs in schools (Madsen et al., 2021; Altman et al., 2022).

Several methodological limitations warrant mention, including first and foremost, the inability to link individual students’ self-reported and objective measurements. In addition, large differences in sample sizes between groups (with an average of only 1 NYCYRBS respondent for every 10 FITNESSGRAM^®^ participants) raise concerns for nonequivalence between the 2 samples. However, these data represent real-world practice, thus enabling us to determine if school-level self-reported BMI can estimate objective BMI, given the data collection methods currently employed in schools. Additionally, given the larger sample of students who fell into the “multiple race” category in the YRBS sample, particular caution should be taken when interpreting findings from multi-racial groups as well as other smaller racial/ethnic groups (American Indian/Alaska Native, Native Hawaiian/Other Pacific Islander). It is possible that increasing the NYCYRBS sample size could help improve the comparability of the two tests, but further research is necessary.

This study finds that high school students’ self-reported BMI, when averaged at the school-level, is not accurate, and may be particularly poor at identifying students at the highest potential metabolic risk based on BMI percentile. These findings provide evidence in support of collecting objective student weight status in schools. However, these results must be carefully considered alongside the growing evidence that many students (particularly females, those who are unhappy with their weight, or perceive themselves to be overweight) are uncomfortable with school-based objective BMI measurements (Madsen et al., 2021; Altman et al., 2022; Thompson and Madsen, 2017; Tatum et al., 2021). If schools were to cease objective BMI measurement, identifying appropriate correction equations for highly diverse student populations would be an important step to ensure self-reported BMI data are accurate and valuable to decision makers.

## Supplementary Material

Supplementary Material

## Figures and Tables

**Table 1:** Participant characteristics (*n* = 90,219) by height and weight data source, 2017–18 school year.

Characteristic	FITNESSGRAM^®^ N (%) *N* = 82,543	NYCYRBS^a^ N (%) *N* = 7676	*p*-value for difference^b^
*Sex*			
Female	40,447 (49.0)	3932 (51.2)	<0.001
male	42,096 (51.0)	3744 (48.8)	
*Age*			
Age 13	2068 (2.5)	193 (2.5)	<0.001
Age 14	17,491 (21.2)	1563 (20.4)	
Age 15	20,013 (24.3)	2058 (26.8)	
Age 16	20,679 (25.1)	1766 (23.0)	
Age 17	18,219 (22.1)	1700 (22.2)	
Age 18	4073 (4.9)	396 (5.2)	
*Grade*			
Grade 9	23,516 (28.5)	1836 (24.1)	<0.001
Grade 10	21,784 (26.4)	2164 (28.4)	
Grade 11	19,045 (23.1)	1678 (22.0)	
Grade 12	18,198 (22.1)	1945 (25.5)	
*Race/ethnicity*			
American Indian/Alaska Native	661 (0.8)	95 (1.3)	<0.001
Asian/Pacific Islander	19,572 (24.0)	1047 (14.1)	
Non-Hispanic Black/African American	15,969 (19.6)	1591 (21.5)	
Native Hawaiian/Other Pacific Islander	442 (0.5)	96 (1.3)	
White	16,550 (20.3)	1034 (14.0)
Hispanic/Latino	28,074 (34.4)	3282 (44.3)
Multi-racial	403 (0.5)	258 (3.5)

a New York City Youth Risk Behavior Survey; data were collected anonymously, so overlap between two samples cannot be determined (i.e. samples are not independent).

b *p*-values for differences calculated using chi-squared tests.

**Table 2:** School-level mean measurement and measurement differences by measurement type (FITNESSGRAM^®^ and New York City Youth Risk Behavior Survey (NYCYRBS)) and student demographic characteristics (*N* = 84 schools).

	FITNESS-GRAM^®^ measurement Mean ± SD	NYCYRBS measurement Mean ± SD	Mean difference^a^ Mean ± SD	95% confidence interval for mean difference^b^
*All students*	*n* = 82,543	*n* = 7676		
Height (m)	1.7 ± 0.02	1.7 ± 0.03	−0.003 ± 0.02	−0.008, 0.001
Weight (kg)	66.6 ± 3.00	65.3 ± 3.50	**−1.38 ± 2.86**	**−1.999, −0.758**
BMI	23.8 ± 1.04	23.4 ± 1.06	**−0.38 ± 0.90**	**−0.574, −0.183**
*Female*	*n* = 40,477	*n* = 3932		
Height (m)	1.6 ± 0.02	1.6 ± 0.02	**−0.01 ± 0.02**	**−0.012, 0.004**
Weight (kg)	62.8 ± 3.26	60.7 ± 378	**−2.16 ± 3.33**	**−2.890, −1.438**
BMI	23.9 ± 1.28	23.3 ± 1.43	**−0.56 ± 1.34**	**−0.857, −0.272**
*Male*	*n* = 42,096	*n* = 3744		
Height (m)	1.7 ± 0.02	1.7 ± 0.02	**0.01 ± 0.02**	**0.002, 0.012**
Weight (kg)	70.2 ± 2.65	69.9 ± 5.57	−0.12 ± 5.38	−1.302, 1.062
BMI	23.6 ± 0.89	23.4 ± 1.60	−0.19 ± 1.57	−0.540, 0.150
*Age 13*	*n* = 2068	*n* = 193		
Height (m)	1.6 ± 0.04	1.6 ± 0.06	0.01 ± 0.06	−0.012, 0.022
Weight (kg)	60.7 ± 6.50	58.6 ± 9.87	**−3.34 ± 9.15**	**−6.127, −0.563**
BMI	22.9 ± 2.13	22.1 ± 3.81	**−1.30 ± 3.34**	**−2.312, −0.284**
*Age 14*	*n* = 17,491	*n* = 1563		
Height (m)	1.6 ± 0.02	1.7 ± 0.04	0.01 ± 0.04	−0.001, 0.015
Weight (kg)	63.0 ± 3.35	62.3 ± 8.16	−0.72 ± 7.75	−2.502, 1.066
BMI	23.1 ± 1.15	22.7 ± 2.88	−0.42 ± 2.85	−1.075, 0.238
*Age 15*	*n* = 20,013	*n* = 2058		
Height (m)	1.7 ± 0.03	1.7 ± 0.04	−0.004 ± 0.03	−0.011, 0.004
Weight (kg)	65.2 ± 2.91	63.5 ± 4.39	**−1.63 ± 4.19**	**−2.550, −0.707**
BMI	23.5 ± 1.11	23.1 ± 1.53	**−0.44 ± 1.49**	**−0.763, −0.107**
*Age 16*	*n* = 20,679	*n* = 1766		
Height (m)	1.7 ± 0.03	1.7 ± 0.04	0.0003 ± 0.04	−0.007, 0.008
Weight (kg)	67.8 ± 3.68	66.7 ± 5.17	**−1.18 ± 4.86**	**−2.229, −0.122**
BMI	24.0 ± 1.15	23.6 ± 1.48	**−0.41 ± 1.44**	**−0.726, −0.103**
*Age 17*	*n* = 18,219	*n* = 1700		
Height (m)	1.7 ± 0.03	1.7 ± 0.04	−0.003 ± 0.04	−0.012, 0.006
Weight (kg)	69.2 ± 3.50	70.0 ± 7.83	0.78 ± 8.87	−0.974, 2.529
BMI	24.2 ± 1.29	24.6 ± 2.49	0.36 ± 2.59	−0.215, 0.937
*Age 18*	*n* = 4073	*n* = 396		
Height (m)	1.7 ± 0.04	1.7 ± 0.07	−0.01 ± 0.08	−0.033, 0.003
Weight (kg)	69.8 ± 4.79	70.2 ± 14.66	0.14 ± 14.93	−3.417, 3.705
BMI	24.2 ± 1.45	24.7 ± 4.43	0.49 ± 4.53	−0.586, 1.574
*AmInd/AlaskNat*	*n* = 661	*n* = 95		
Height (m)	1.7 ± 0.05	1.7 ± 0.11	0.01 ± 0.12	−0.028, 0.046
Weight (kg)	65.5 ± 10.2	62.5 ± 10.45	**−4.54 ± 11.53**	**−8.227, −0.850**
BMI	23.5 ± 3.23	22.2 ± 3.16	**−1.81 ± 3.77**	**−3.019, −0.606**
*Asian/PI*	n = 19,572	*n* = 1047		
Height (m)	1.7 ± 0.04	1.7 ± 0.06	0.003 ± 0.05	−0.009, 0.016
Weight (kg)	62.0 ± 5.50	61.6 ± 7.59	−0.28 ± 7.19	−1.996, 1.432
BMI	22.4 ± 1.56	22.2 ± 1.98	−0.21 ± 1.97	−0.677, 0.265
*Black/AfAm*	*n* = 15,969	*n* = 1591		
Height (m)	1.7 ± 0.02	1.7 ± 0.04	0.001 ± 0.04	−0.007, 0.010
Weight (kg)	69.2 ± 3.12	69.7 ± 8.63	0.55 ± 8.52	−1.326, 2.417
BMI	24.1 ± 1.09	24.2 ± 2.29	0.08 ± 2.37	−0.446, 0.596
*Hawaiian/OthPI*	*n* = 442	*n* = 96		
Height (m)	1.7 ± 0.06	1.7 ± 0.08	0.02 ± 0.10	−0.010, 0.057
Weight (kg)	63.0 ± 12.04	64.6 ± 14.24	3.57 ± 16.91	−2.071, 9.203
BMI	23.0 ± 4.19	22.7 ± 3.92	0.69 ± 4.62	−0.855, 2.229
*White*	*n* = 16,550	*n* = 1034		
Height (m)	1.7 ± 0.03	1.7 ± 0.06	**0.02 ± 0.07**	**0.006, 0.041**
Weight (kg)	67.9 ± 6.2	66.3 ± 11.11	−0.77 ± 10.99	−3.584, 2.043
BMI	23.8 ± 2.00	22.7 ± 3.79	−0.79 ± 3.68	−1.733, 0.151
*Hispanic/Latino*	*n* = 28,074	*n* = 3282		
Height (m)	1.7 ± 0.02	1.7 ± 0.03	−0.003 ± 0.03	−0.010, 0.003
Weight (kg)	66.5 ± 2.88	64.9 ± 3.72	**−1.56 ± 4.24**	**−2.476, −0.636**
BMI	24.1 ± 0.97	23.7 ± 1.13	**−0.43 ± 1.27**	**−0.711, −0.158**
*Multi-racial*	*n* = 403	*n* = 258		
Height (m)	1.7 ± 0.07	1.7 ± 0.07	−0.01 ± 0.09	−0.042, 0.012
Weight (kg)	64.7 ± 9.96	64.1 ± 11.31	1.21 ± 13.78	−2.931, 5.348
BMI	22.8 ± 2.70	23.2 ± 3.49	0.83 ± 4.43	−0.501, 2.159

NYCYRBS = New York City Youth Risk Behavior Survey; BMI = Body Mass Index; AmInd/AlaskNat = American Indian/ Alaska Native; PI=Pacific Islander; AfAm = African American; Oth = Other.

a Mean difference calculated as NYCYRBS minus FITNESSGRAM^®^; a negative mean difference suggests that student self-report under-estimates the true value.

b 95% confidence interval for mean difference between the FITNESSGRAM^®^ and NYCYRBS measurement calculated using paired *t*-tests; bolded when statistically significant at *p* < 0.05.

**Table 3:** School-level proportion of students in Body Mass Index (BMI) categories,^a^ by measurement type (FITNESSGRAM^®^ and New York City Youth Risk Behavior Survey (NYCYRBS)) and student demographic characteristics (N = 84 schools).

	FITNESS GRAM^®^ -measured % Mean ± SD	NYCYRBS- measured % Mean ± SD	Mean difference in proportion^b^ Mean ± SD	95% confidence interval for mean difference^c^
*All students*	*n* = 82,543	*n* = 7676		
Underweight	2.6 ± 1.5	3.2 ± 2.5	**0.59 ± 2.41**	**0.069, 1.115**
Healthy weight	60.6 ± 7.6	62.9 ± 9.1	**2.26 ± 8.42**	**0.429, 4.084**
Overweight	18.4 ± 3.3	18.0 ± 5.7	−0.39 ± 5.40	−1.566, 0.778
Obesity	18.4 ± 5.7	15.9 ± 6.2	**−2.45 ± 6.09**	**−3.776, −1.132**
Severe obesity	6.4 ± 3.0	5.0 ± 3.3	**−1.36 ± 3.65**	**−2.151, −0.569**
*Female*	*n* = 40,477	*n* = 3932		
Underweight	2.1 ± 1.5	2.4 ± 4.2	0.36 ± 4.36	−0.583, 1.311
Healthy weight	60.5 ± 11.3	65.5 ± 13.8	**4.94 ± 16.25**	**1.418, 8.472**
Overweight	19.7 ± 4.8	19.3 ± 10.1	−0.47 ± 8.92	−2.407, 1.465
Obesity	16.5 ± 6.9	12.8 ± 7.7	**−3.65 ± 8.28**	**−5.443, −1.850**
Severe obesity	5.6 ± 3.4	3.6 ± 3.8	**−2.05 ± 4.64**	**−3.060, −1.047**
*Male*	*n* = 42,096	*n* = 3744		
Underweight	3.1 ± 1.8	4.2 ± 3.9	**1.04 ± 3.98**	**0.174, 1.914**
Healthy weight	59.6 ± 9.9	60.8 ± 9.6	1.12 ± 11.51	−1.391, 3.637
Overweight	16.5 ± 4.1	16.4 ± 7.0	−0.07 ± 8.42	−1.908, 1.770
Obesity	19.5 ± 6.2	18.6 ± 8.6	−0.89 ± 7.70	−2.575, 0.789
Severe obesity	6.8 ± 3.3	6.2 ± 5.8	−0.57 ± 5.76	−1.827, 0.688
*Age 13*	*n* = 2068	*n* = 193		
Underweight	1.2 ± 2.6	3.1 ± 16.3	1.90 ± 15.82	−1.694, 5.487
Healthy weight	49.6 ± 26.8	31.6 ± 35.1	**−17.95 ± 44.67**	**−28.090, −7.814**
Overweight	19.4 ± 17.9	22.5 ± 31.5	3.13 ± 33.06	−4.379, 10.629
Obesity	19.5 ± 18.2	10.3 ± 21.9	**−9.15 ± 27.34**	**−15.354, −2.942**
Severe obesity	7.3 ± 10.4	4.5 ± 17.9	−2.73 ± 22.20	−7.765, 2.311
*Age 14*	*n* = 17,491	*n* = 1563		
Underweight	1.6 ± 1.7	2.0 ± 4.3	0.40 ± 4.39	−0.553, 1.350
Healthy weight	57.8 ± 11.4	54.5 ± 28.4	−3.26 ± 30.74	−9.932, 3.411
Overweight	20.0 ± 7.4	18.0 ± 16.2	−2.00 ± 17.89	−5.883, 1.881
Obesity	20.6 ± 7.1	14.7 ± 16.8	**−5.85 ± 16.04**	**−9.332, −2.371**
Severe obesity	6.8 ± 4.1	5.7 ± 13.0	−1.04 ± 13.44	−3.953, 1.882
*Age 15*	*n* = 20,013	*n* = 2058		
Underweight	2.1 ± 2.0	2.4 ± 4.6	0.23 ± 5.10	−0.874, 1.337
Healthy weight	59.5 ± 9.1	61.0 ± 18.6	1.50 ± 17.41	−2.279, 5.275
Overweight	19.5 ± 5.1	18.7 ± 12.6	−0.81 ± 12.86	−3.601, 1.981
Obesity	18.9 ± 7.0	15.6 ± 12.1	**−3.30 ± 12.69**	**−6.055, −0.546**
Severe obesity	6.7 ± 3.7	4.7 ± 6.3	**−2.00 ± 6.75**	**−3.458, −0.531**
*Age 16*	*n* = 20,679	*n* = 1766		
Underweight	2.5 ± 1.9	3.9 ± 7.4	1.36 ± 7.72	−0.320, 3.032
Healthy weight	61.3 ± 8.4	64.2 ± 14.9	2.81 ± 15.16	−0.476, 6.104
Overweight	17.7 ± 4.3	15.6 ± 9.4	−2.08 ± 9.99	−4.249, 0.087
Obesity	18.5 ± 7.1	16.4 ± 10.9	−2.09 ± 11.37	−4.556, 0.377
Severe obesity	6.2 ± 3.3	4.5 ± 5.4	**−1.71 ± 5.61**	**−2.929, −0.492**
*Age 17*	*n* = 18,219	*n* = 1700		
Underweight	3.3 ± 2.4	4.3 ± 6.4	0.93 ± 6.07	−0.389, 2.247
Healthy weight	63.5 ± 9.5	56.5 ± 22.1	**−6.96 ± 23.10**	**−11.976, −1.950**
Overweight	16.6 ± 5.0	15.0 ± 12.1	−1.66 ± 12.36	−4.347, 1.020
Obesity	16.6 ± 6.9	19.5 ± 17.9	2.94 ± 19.06	−1.202, 7.072
Severe obesity	6.0 ± 4.1	6.8 ± 13.0	0.77 ± 13.38	−2.130, 3.677
*Age 18*	*n* = 4073	*n* = 396		
Underweight	5.4 ± 4.5	6.3 ± 12.1	0.95 ± 12.24	−1.706, 3.605
Healthy weight	64.4 ± 12.8	52.2 ± 35.8	**−12.14 ± 35.76**	**−19.903, −4.382**
Overweight	16.2 ± 8.5	12.8 ± 22.2	−3.47 ± 22.90	−8.438, 1.500
Obesity	14.0 ± 8.0	12.0 ± 20.4	−2.00 ± 22.15	−6.811, 2.802
Severe obesity	4.6 ± 5.0	4.9 ± 13.2	0.03 ± 14.08	−3.022, 3.091
*American Indian/Alaska Native*	*n* = 661	*n* = 95		
Underweight	4.6 ± 10.4	5.5 ± 19.0	0.91 ± 19.68	−3.527, 5.346
Healthy weight	55.7 ± 31.6	37.5 ± 44.0	**−18.22 ± 62.52**	**−32.316, −4.124**
Overweight	15.4 ± 20.9	12.2 ± 28.6	−3.27 ± 34.65	−11.079, 4.547
Obesity	16.6 ± 21.9	3.9 ± 14.9	**−12.76 ± 27.14**	**−18.875, −6.639**
Severe obesity	5.6 ± 15.6	1.3 ± 7.1	**−4.30 ± 17.45**	**−8.236, −0.369**
*Asian/ Pacific Islander (PI)*	*n* = 19,572	*n* = 1047		
Underweight	4.4 ± 6.1	3.1 ± 6.0	−1.28 ± 8.34	−3.111, 0.554
Healthy weight	69.2 ± 16.7	59.5 ± 34.4	**−9.72 ± 38.56**	**−18.187, −1.243**
Overweight	15.2 ± 10.9	13.2 ± 24.0	−2.08 ± 25.78	−7.748, 3.582
Obesity	11.1 ± 9.1	9.6 ± 14.9	−1.56 ± 17.54	−5.411, 2.296
Severe obesity	2.9 ± 5.1	1.7 ± 6.5	−1.24 ± 7.78	−2.952, 0.466
*Black/African American*	*n* = 15,969	*n* = 1591		
Underweight	2.4 ± 1.7	2.2 ± 3.8	−0.22 ± 4.21	−1.130, 0.697
Healthy weight	60.4 ± 8.0	58.0 ± 19.8	−2.39 ± 20.28	−6.790, 2.011
Overweight	17.9 ± 5.3	18.2 ± 14.2	0.34 ± 14.62	−2.835, 3.510
Obesity	19.3 ± 6.0	19.2 ± 14.2	−0.11 ± 15.06	−3.381, 3.157
Severe obesity	8.0 ± 3.8	8.1 ± 10.5	0.11 ± 10.60	−2.195, 2.405
*Native Hawaiian/other PI*	n = 442	n = 96		
Underweight	2.3 ± 7.5	6.3 ± 19.6	3.96 ± 20.76	−0.812, 8.741
Healthy weight	59.1 ± 18.1	47.2 ± 40.6	**−20.06 ± 58.47**	**−33.516, −6.610**
Overweight	10.8 ± 21.1	7.4 ± 20.4	−3.46 ± 27.90	−9.882, 2.956
Obesity	14.0 ± 24.5	9.6 ± 25.6	−4.44 ± 35.68	−12.647, 3.770
Severe obesity	3.1 ± 13.6	2.3 ± 13.1	−0.80 ± 19.26	−5.236, 3.628
***White***	*n* = 16,550	*n* = 1034		
Underweight	2.7 ± 5.1	3.1 ± 11.96	0.46 ± 9.61	−1.622, 2.549
Healthy weight	59.1 ± 18.1	47.2 ± 40.6	**−11.94 ± 40.59**	**−20.745, −3.126**
Overweight	19.6 ± 14.6	14.4 ± 26.0	−5.22 ± 30.55	−11.845, 1.413
Obesity	18.6 ± 13.4	7.9 ± 18.0	**−10.69 ± 19.24**	**−14.868, −6.518**
Severe obesity	5.9 ± 7.4	2.9 ± 12.5	−2.98 ± 14.26	−6.076, 0.112
*Hispanic/Latino*	*n* = 28,074	*n* = 3282		
Underweight	1.9 ± 1.4	2.6 ± 3.4	0.69 ± 3.64	−0.102, 1.476
Healthy weight	57.7 ± 7.5	59.7 ± 12.5	1.96 ± 13.87	−1.047, 4.973
Overweight	20.2 ± 4.7	21.3 ± 11.8	1.13 ± 12.12	−1.503, 3.758
Obesity	20.2 ± 6.8	16.4 ± 7.6	**−3.78 ± 9.00**	**−5.731, −1.823**
Severe obesity	6.7 ± 3.5	4.7 ± 3.9	**−1.99 ± 4.96**	**−3.063, −0.909**
*Multi-racial*	*n* = 403	*n* = 258		
Underweight	1.0 ± 4.4	1.9 ± 7.0	0.91 ± 7.24	−0.729, 2.557
Healthy weight	49.1 ± 41.2	58.8 ± 37.0	9.70 ± 54.94	−2.769, 22.172
Overweight	9.7 ± 17.4	17.1 ± 27.8	**7.40 ± 30.54**	**0.469, 14.334**
Obesity	7.7 ± 17.9	13.1 ± 25.3	5.36 ± 31.82	−1.863, 12.581
Severe obesity	2.1 ± 7.5	3.1 ± 8.9	1.00 ± 11.44	−1.597, 3.599

a Weight status categories for BMI based on age- and sex-specific criteria set by US Center for disease Control’s 2022 growth charts; underweight ≤5th %; healthy weight = 5th to <85th %; overweight = ≥85th and < 95th %; obesity = ≥95th % severe obesity = ≥120% of the 95th % or BMI ≥ 35.

b Mean difference calculated as NYCYRBS minus FITNESSGRAM^®^; a negative mean difference signifies NYCYRBS measure is underreported compared to the FITNESSGRAM^®^ measure.

c 95% confidence interval for mean difference between the FITNESSGRAM^®^ and NYCYRBS measurements calculated using paired *t*-tests; bolded when statistically significant at *p* < 0.05.

**Table 4:** School-level predictors of school-level differences between FITNESSGRAM^®^- and New York City Youth Risk Behavior Survey (NYCYRBS)-measured body mass index (BMI) and proportion of students ^3^95th % for BMI (*n* = 84 schools; *n* = 90,883 students).^a^

School-level characteristics	Difference^b^ in BMI between FITNESSGRAM^®^ and NYCYRBS measurements mean ± SE	95% CI for mean difference	Difference^b^ in proportion of students classified as having obesity^c^ % ± SE	95% CI for difference in proportion	Difference^b^ in proportion of students classified as having severe obesity^c^ % ± SE	95% CI for difference in proportion
Total school enrollment (per 100 students)	0.01 ± 0.01	−0.012, 0.028	0.001 ± 0.001	−0.001, 0.001	0.0002 ± 0.0004	−0.001, 0.001
Proportion of NYCYRBS to FITNESSGRAM^®^ students measured	0.01 ± 0.01	−0.002, 0.023	0.001 ± 0.001	−0.0002, 0.001	0.0003 ± 0.0003	−0.0002, 0.001
Proportion of students who qualify for free or reduced-price meals	1.01 ± 0.66	−0.312, 2.322	**0.11 ± 0.4**	**0.017, 0.193**	**0.02 ± 0.03**	**−0.036, 0.073**

a Estimates determined by multivariate models including all 3 variables (total school enrollment; proportion of NYCYRBS to FITNESSGRAM^®^ students measured; and proportion of students who qualify for free or reduced-price meals).

b Mean differences calculated as NYCYRBS minus FITNESSGRAM^®^; a negative mean difference signifies NYCYRBS measure is underreported compared to the FITNESSGRAM^®^ measure.

c Weight status categories for BMI based on age- and sex-specific criteria set by US Center for disease Control’s 2022 growth charts; obesity ≥95th %; severe obesity = ≥120% of the 95th % or BMI ≥35.

## Data Availability

The authors do not have permission to share data.
