# Supplementary material for: School-level self-reported versus objective measurements of body mass index in public high school students
Source: Prev Med. Author manuscript; Available in PMC 2023 Sep 27. (PMC10529345; doi:10.1016/j.ypmed.2023.107616)
Supplement: Supplementary Material [file NIHMS1921930-supplement-Supplementary_Material.docx]

**Appendix Table 1: School-level mean measurement and measurement differences for height, weight, and Body Mass Index (BM) by measurement type (FITNESSGRAM® and New York City Youth Risk Behavior Survey (NYCYRBS)), stratified by student sex and age (N=84 schools), all students**

|  | FITNESS-GRAM® measurement  (n=83,193 students)  Mean ± SD | NYCYRBS measurement  (N=7,690 students)  Mean ± SD | Mean difference^A^  Mean ± SD | 95% confidence interval for mean difference^B^ |
| --- | --- | --- | --- | --- |
| Female Age 13 | n=1,059 | n=111 |  |  |
| Height (m) | 1.6 ± 0.04 | 1.6 ± 0.06 | **0.02 ± 0.06** | **0.001, 0.043** |
| Weight (kg) | 60.7 ± 10.63 | 57.7 ± 10.56 | −2.14 ± 8.08 | 4.965, 0.676 |
| BMI | 23.4 ± 3.28 | 22.1 ± 3.55 | **-1.46 ± 2.95** | **-2.489, -0.433** |
| Male Age 13 | n=1,009 | n=82 |  |  |
| Height (m) | 1.6 ± 0.05 | 1.7 ± 0.08 | 0.004 ± 0.07 | -0.024, 0.033 |
| Weight (kg) | 61.6 ± 6.70 | 59.2 ± 11.83 | −2.95 ± 11.33 | -7.260, 1.356 |
| BMI | 22.5 ± 2.02 | 21.6 ± 4.06 | −1.13 ± 3.62 | -2.505, 0.248 |
| Female Age 14 | n=8,734 | n=817 |  |  |
| Height (m) | 1.6 ± 0.02 | 1.6 ± 0.03 | −0.0004 ± 0.04 | -0.009, 0.008 |
| Weight (kg) | 60.4 ± 3.96 | 59.0 ± 9.13 | −1.41 ± 9.44 | -3.629, 0.806 |
| BMI | 23.2 ± 1.56 | 22.7 ± 3.20 | −0.51 ± 3.31 | -1.292, 0.263 |
| Male Age 14 | n=8,757 | n=746 |  |  |
| Height (m) | 1.7 ± 0.03 | 1.7 ± 0.04 | **0.02 ± 0.05** | **0.006, 0.030** |
| Weight (kg) | 65.4 ± 4.01 | 65.5 ± 13.40 | 0.22 ± 13.18 | -2.925, 3.362 |
| BMI | 22.9 ± 1.26 | 22.6 ± 4.64 | −0.33 ± 4.72 | -1.456, 0.793 |
| Female Age 15 | n=10,017 | n=1,100 |  |  |
| Height (m) | 1.6 ± 0.02 | 1.6 ± 0.04 | −0.01 ± 0.04 | -0.014, 0.004 |
| Weight (kg) | 61.8 ± 3.36 | 60.9 ± 7.22 | −0.85 ± 7.00 | -2.411, 0.703 |
| BMI | 23.6 ± 1.37 | 23.5 ± 2.82 | −0.12 ± 2.74 | -0.726, 0.495 |
| Male Age 15 | n=9,996 | n=958 |  |  |
| Height (m) | 1.7 ± 0.02 | 1.7 ± 0.04 | 0.005 ± 0.04 | -0.004, 0.014 |
| Weight (kg) | 68.5 ± 3.36 | 67.5 ± 6.87 | −1.10 ± 7.22 | -2.754, 0.545 |
| BMI | 23.4 ± 1.13 | 22.9 ± 2.43 | −0.45 ± 2.46 | -1.008, 0.115 |
| Female Age 16 | n=10,171 | n=893 |  |  |
| Height (m) | 1.6 ± 0.02 | 1.6 ± 0.03 | **-0.01 ± 0.03** | **-0.017, -0.001** |
| Weight (kg) | 63.7 ± 3.95 | 60.5 ± 6.26 | **-2.90 ± 5.89** | **-4.212, -1.592** |
| BMI | 24.2 ± 1.48 | 23.3 ± 2.22 | **-0.83 ± 2.00** | **-1.279, -0.390** |
| Male Age 16 | n=10,508 | n=873 |  |  |
| Height (m) | 1.7 ± 0.02 | 1.7 ± 0.04 | 0.01 ± 0.04 | -0.003, 0.016 |
| Weight (kg) | 71.5 ± 3.32 | 71.1 ± 8.18 | −0.28 ± 7.87 | -2.017, 1.464 |
| BMI | 23.7 ± 1.08 | 23.5 ± 2.58 | −0.25 ± 2.52 | -0.807, 0.308 |
| Female Age 17 | n=8,750 | n=844 |  |  |
| Height (m) | 1.6 ± 0.02 | 1.6 ± 0.04 | −0.01 ± 0.04 | -0.018, 0.002 |
| Weight (kg) | 64.7 ± 4.15 | 64.4 ± 8.74 | −0.43 ± 8.53 | -2.364, 1.508 |
| BMI | 24.4 ± 1.61 | 24.5 ± 3.14 | 0.13 ± 3.18 | -0.593, 0.850 |
| Male Age 17 | n=9,469 | n=856 |  |  |
| Height (m) | 1.7 ± 0.02 | 1.8 ± 0.04 | 0.01 ± 0.04 | -0.002, 0.016 |
| Weight (kg) | 73.4 ± 3.46 | 75.8 ± 8.72 | **2.34 ± 8.39** | **0.421, 4.254** |
| BMI | 24.1 ± 1.14 | 24.7 ± 2.79 | 0.61 ± 2.70 | -0.009, 1.224 |
| Female Age 18 | n=1,716 | n=1667 |  |  |
| Height (m) | 1.6 ± 0.03 | 1.6 ± 0.06 | **−0.02 ± 0.07** | **-0.038, -0.003** |
| Weight (kg) | 64.3 ± 6.16 | 64.0 ± 16.33 | −0.51 ± 16.37 | -4.854, 3.833 |
| BMI | 24.3 ± 2.26 | 24.7 ± 5.04 | 0.35 ± 5.05 | -0.990, 1.691 |
| Male Age 18 | n=2,357 | n=229 |  |  |
| Height (m) | 1.7 ± 0.03 | 1.7 ± 0.07 | −0.004 ± 0.07 | -0.022, 0.014 |
| Weight (kg) | 73.8 ± 5.71 | 74.2 ± 12.16 | 0.05 ± 12.48 | -3.292, 3.391 |
| BMI | 24.1 ± 1.66 | 24.4 ± 4.00 | 0.24 ± 4.09 | -0.853, 1.335 |

NYCYRBS = New York City Youth Risk Behavior Survey; BMI = Body Mass Index; AmInd/AlaskNat = American Indian/ Alaska Native; PI=Pacific Islander; AfAm = African American; Oth=Other

^A^ Mean difference calculated as NYCYRBS minus FITNESSGRAM®; a negative mean difference signifies NYCYRBS measure is underreported compared to the FITNESSGRAM® measure

^B^ 95% confidence interveal for mean difference calculated using paired t-tests; bolded when statistically significant at p<0.05

**Appendix Table 2: School-level mean measurement and measurement differences for height, weight, and Body Mass Index (BM) by measurement type (FITNESSGRAM® and New York City Youth Risk Behavior Survey (NYCYRBS)), stratified by student sex and age (N=84 schools), students with obesity**

|  | FITNESS-GRAM® measurement  (n=83,193 students)  Mean ± SD | NYCYRBS measurement  (N=7,690 students)  Mean ± SD | Mean difference^A^  Mean ± SD | 95% confidence interval for mean difference^B^ |
| --- | --- | --- | --- | --- |
| Female Age 13 | n=174 | n=14 |  |  |
| Height (m) | 1.6 ± 0.05 | 1.6 ± 0.08 | 0.06 ± 0.08 | -0.005, 0.115 |
| Weight (kg) | 82.5 ± 10.91 | 80.3 ± 13.10 | 0.70 ± 15.50 | -11.213, 12.623 |
| BMI | 31.6 ± 3.79 | 30.1 ± 2.93 | -1.87 ± 4.22 | -5.112, 1.378 |
| Male Age 13 | n=209 | n=18 |  |  |
| Height (m) | 1.7 ± 0.06 | 1.7 ± 0.08 | **0.07 ± 0.07** | **0.008, 0.128** |
| Weight (kg) | 82.5 ± 10.82 | 81.0 ± 9.31 | 6.54 ± 15.82 | -6.683, 19.765 |
| BMI | 29.7 ± 3.04 | 29.1 ± 3.40 | 0.12 ± 4.17 | -3.364, 3.607 |
| Female Age 14 | n=1,317 | n=92 |  |  |
| Height (m) | 1.6 ± 0.03 | 1.6 ± 0.06 | -0.01 ± 0.07 | -0.035, 0.006 |
| Weight (kg) | 83.3 ± 5.25 | 81.7 ± 11.70 | -1.62 ± 13.02 | -5.623, 2.392 |
| BMI | 31.6 ± 2.00 | 31.8 ± 4.41 | 0.02 ± 5.00 | -1.520, 1.557 |
| Male Age 14 | n=1,742 | n=129 |  |  |
| Height (m) | 1.7 ± 0.04 | 1.7 ± 0.08 | 0.01 ± 0.09 | -0.020, 0.032 |
| Weight (kg) | 87.8 ± 5.02 | 88.8 ± 15.21 | 0.30 ± 15.60 | -4.336, 4.931 |
| BMI | 30.4 ± 1.37 | 30.5 ± 4.55 | -0.07 ± 5.01 | -1.555, 1.423 |
| Female Age 15 | n=1,479 | n=133 |  |  |
| Height (m) | 1.6 ± 0.03 | 1.6 ± 0.07 | **-0.02 ± 0.08** | **-0.045, -0.004** |
| Weight (kg) | 86.1 ± 5.23 | 82.8 ± 10.04 | **-3.60 ± 10.83** | **-6.500, -0.696** |
| BMI | 32.8 ± 1.57 | 32.4 ± 3.23 | -0.35 ± 3.31 | -1.234, 0.539 |
| Male Age 15 | n=1,837 | n=170 |  |  |
| Height (m) | 1.7 ± 0.03 | 1.7 ± 0.09 | -0.004 ± 0.09 | -0.026, 0.019 |
| Weight (kg) | 92.9 ± 4.73 | 91.0 ± 12.80 | -2.23 ± 12.87 | -5.615, 1.155 |
| BMI | 31.5 ± 1.24 | 30.9 ± 2.83 | -0.67 ± 2.95 | -1.449, 0.100 |
| Female Age 16 | n=1,395 | n=114 |  |  |
| Height (m) | 1.6 ± 0.03 | 1.6 ± 0.06 | -0.01 ± 0.06 | -0.032, 0.002 |
| Weight (kg) | 88.6 ± 5.06 | 85.0 ± 9.32 | **-4.11 ± 9.54** | **-6.796, -1.430,** |
| BMI | 33.4 ± 1.56 | 32.4 ± 2.52 | **-1.00 ± 2.50** | **-1.703, -0.295** |
| Male Age 16 | n=1,866 | n=148 |  |  |
| Height (m) | 1.7 ± 0.03 | 1.7 ± 0.07 | 0.01 ± 0.08 | -0.014, 0.026 |
| Weight (kg) | 97.7 ± 5.00 | 98.9 ± 15.11 | 1.09 ± 14.81 | -2.639, 4.821 |
| BMI | 32.2 ± 1.50 | 32.3 ± 4.02 | 0.11 ± 3.66 | -0.808, 1.038 |
| Female Age 17 | n=1,133 | n=117 |  |  |
| Height (m) | 1.6 ± 0.04 | 1.6 ± 0.05 | **-0.02 ± 0.06** | **-0.039, -0.005** |
| Weight (kg) | 92.3 ± 6.32 | 90.8 ± 11.56 | -2.80 ± 12.53 | -6.433, 0.841 |
| BMI | 34.5 ± 1.74 | 34.7 ± 4.19 | -0.06 ± 4.57 | -1.393, 1.263 |
| Male Age 17 | n=1,491 | n=172 |  |  |
| Height (m) | 1.8 ± 0.03 | 1.7 ± 0.08 | -0.01 ± 0.08 | -0.029, 0.013 |
| Weight (kg) | 101.7 ± 7.24 | 100.3 ± 12.38 | -1.44 ± 12.84 | -4.597, 1.714 |
| BMI | 33.1 ± 1.87 | 33.1 ± 3.56 | -0.09 ± 3.38 | -0.921, 0.740 |
| Female Age 18 | n=201 | n=19 |  |  |
| Height (m) | 1.6 ± 0.05 | 1.6 ± 0.08 | -0.01 ± 0.06 | -0.052, 0.029 |
| Weight (kg) | 92.0 ± 9.49 | 95.4 ± 22.84 | 0.63 ± 12.78 | -7.960, 9.215 |
| BMI | 35.0 ± 2.95 | 36.2 ± 5.43 | 0.64 ± 3.02 | -1.389, 2.664 |
| Male Age 18 | n=357 | n=41 |  |  |
| Height (m) | 1.7 ± 0.06 | 1.7 ± 0.09 | **-0.04 ± 0.09** | **-0.073, -0.001** |
| Weight (kg) | 102.5 ± 9.64 | 103.59 ± 22.07 | 0.59 ± 23.73 | -8.795, 9.981 |
| BMI | 33.8 ± 4.18 | 35.4 ± 6.34 | 1.68 ± 7.19 | -1.165, 4.525 |

NYCYRBS = New York City Youth Risk Behavior Survey; BMI = Body Mass Index; AmInd/AlaskNat = American Indian/ Alaska Native; PI=Pacific Islander; AfAm = African American; Oth=Other

^A^ Weight status categories for BMI based on age- and sex-specific criteria set by US Center for Disease Control’s 2022 growth charts; Obesity = ≥95^th^ %

^B^Mean difference calculated as NYCYRBS minus FITNESSGRAM®; a negative mean difference signifies NYCYRBS measure is underreported compared to the FITNESSGRAM® measure

^C^ 95% confidence interval for mean difference calculated using paired t-tests; bolded when statistically significant at p<0.05

**Appendix Table 3: School-level mean measurement and measurement differences for height, weight, and Body Mass Index (BM) by measurement type (FITNESSGRAM® and New York City Youth Risk Behavior Survey (NYCYRBS)), stratified by student sex and age (N=84 schools), students with severe obesity^A,B^**

|  | FITNESS-GRAM® measurement  (n=4,235 students)  Mean ± SD | NYCYRBS measurement  (N=361 students)  Mean ± SD | Mean difference^C^  Mean ± SD | 95% confidence interval for mean difference^D^ |
| --- | --- | --- | --- | --- |
| Female Age 14 | n=393 | n=30 |  |  |
| Height (m) | 1.6 ± 0.06 | 1.6 ± 0.08 | -0.01 ± 0.07 | -0.048, 0.022 |
| Weight (kg) | 98.4 ± 10.41 | 100.4 ± 16.49 | 4.44 ± 18.10 | -4.560, 13.441 |
| BMI | 37.0 ± 2.61 | 38.8 ± 7.58 | 2.69 ± 8.40 | -1.483, 6.869 |
| Male Age 14 | n=543 | n=38 |  |  |
| Height (m) | 1.7 ± 0.04 | 1.7 ± 0.10 | -0.04 ± 0.10 | -0.086, 0.009 |
| Weight (kg) | 100.8 ± 8.22 | 100.3 ± 17.87 | **-5.94 ± 12.35** | **-11.722, -0.167** |
| BMI | 34.8 ± 2.19 | 35.3 ± 4.68 | -0.50 ± 1.74 | -1.316, 0.312 |
| Female Age 15 | n=451 | n=33 |  |  |
| Height (m) | 1.6 ± 0.05 | 1.6 ± 0.07 | **-0.04 ± 0.07** | **-0.071, -0.013** |
| Weight (kg) | 100.1 ± 8.30 | 96.6 ± 9.54 | **-4.68 ± 10.38** | **-9.059, -0.291** |
| BMI | 37.9 ± 2.18 | 38.8 ± 3.13 | 0.27 ± 3.49 | -1.199, 1.746 |
| Male Age 15 | n=621 | n=53 |  |  |
| Height (m) | 1.7 ± 0.04 | 1.7 ± 0.10 | -0.01 ± 0.09 | -0.046, 0.020 |
| Weight (kg) | 105.3 ± 8.43 | 103.7 ± 14.49 | -1.86 ± 14.49 | -7.171, 3.459 |
| BMI | 35.8 ± 1.86 | 35.7 ± 2.68 | -0.18 ± 3.45 | -1.441, 1.089 |
| Female Age 16 | n=434 | n=26 |  |  |
| Height (m) | 1.6 ± 0.04 | 1.6 ± 0.05 | -0.01 ± 0.05 | -0.039, 0.013 |
| Weight (kg) | 103.2 ± 8.15 | 105.8 ± 10.67 | -1.36 ± 11.19 | -6.924, 4.205 |
| BMI | 38.9 ± 2.41 | 39.4 ± 3.91 | 0.24 ± 3.40 | -1.448, 1.933 |
| Male Age 16 | n=594 | n=51 |  |  |
| Height (m) | 1.7 ± 0.04 | 1.7 ± 0.09 | -0.0002 ± 0.10 | -0.034, 0.033 |
| Weight (kg) | 113.4 ± 9.74 | 114.1 ± 16.65 | 0.03 ± 16.08 | -5.581, 5.640 |
| BMI | 37.4 ± 2.53 | 37.3 ± 4.13 | 0.04 ± 3.45 | -1.170, 1.240 |
| Female Age 17 | n=420 | n=42 |  |  |
| Height (m) | 1.6 ± 0.05 | 1.6 ± 0.06 | -0.03 ± 0.08 | -0.065, 0.008 |
| Weight (kg) | 107.5 ± 12.60 | 110.1 ± 20.63 | 1.72 ± 24.56 | -9.173, 12.605 |
| BMI | 39.9 ± 3.07 | 42.2 ± 8.34 | 2.27 ± 8.06 | -1.307, 5.840 |
| Male Age 17 | n=464 | n=55 |  |  |
| Height (m) | 1.8 ± 0.05 | 1.7 ± 0.10 | 0.003 ± 0.11 | -0.037, 0.044 |
| Weight (kg) | 117.9 ± 10.35 | 119.0 ± 16.03 | 2.59 ± 18.55 | -3.987, 9.170 |
| BMI | 38.3 ± 2.30 | 39.6 ± 5.15 | 0.78 ± 5.68 | -1.234. 2.796 |
| Female Age 18 | n=81 | n=10 |  |  |
| Height (m) | 1.6 ± 0.08 | 1.7 ± 0.08 | -0.01 ± 0.06 | -0.097, 0.078 |
| Weight (kg) | 106.7 ± 12.60 | 118.4 ± 24.67 | -0.22 ± 12.69 | -20.422, 19.973 |
| BMI | 40.8 ± 2.98 | 42.1 ± 5.36 | 0.24 ± 2.57 | -3.849, 4.333 |
| Male Age 18 | n=111 | n=14 |  |  |
| Height (m) | 1.7 ± 0.10 | 1.7 ± 0.12 | 0.01 ± 0.10 | -0.063, 0.075 |
| Weight (kg) | 120.2 ± 16.37 | 119.3 ± 21.25 | 4.31 ± 28.94 | -16.389, 25.019 |
| BMI | 39.4 ± 4.99 | 41.7 ± 7.09 | 1.03 ± 6.88 | -3.887, 5.955 |

NYCYRBS = New York City Youth Risk Behavior Survey; BMI = Body Mass Index; AmInd/AlaskNat = American Indian/ Alaska Native; PI=Pacific Islander; AfAm = African American; Oth=Other

^A^ Weight status categories for BMI based on age- and sex-specific criteria set by US Center for Disease Control’s 2022 growth charts; severe obesity = ≥120% of the 95^th^ % or BMI≥35

^B^ Insufficient cell size precluded analysis for age 13 students

^C^Mean difference calculated as NYCYRBS minus FITNESSGRAM®; a negative mean difference signifies NYCYRBS measure is underreported compared to the FITNESSGRAM® measure

^D^ 95% confidence interval for mean difference calculated using paired t-tests; bolded when statistically significant at p<0.05

**Appendix Figures: Bland-Altman Plots of Height, Weight, and Body Mass Index (BMI) for all students, and stratified by sex, age, and race/ethnicity**

**Figure 1: Bland-Altman Plots of height, weight, and BMI, for all students**

**
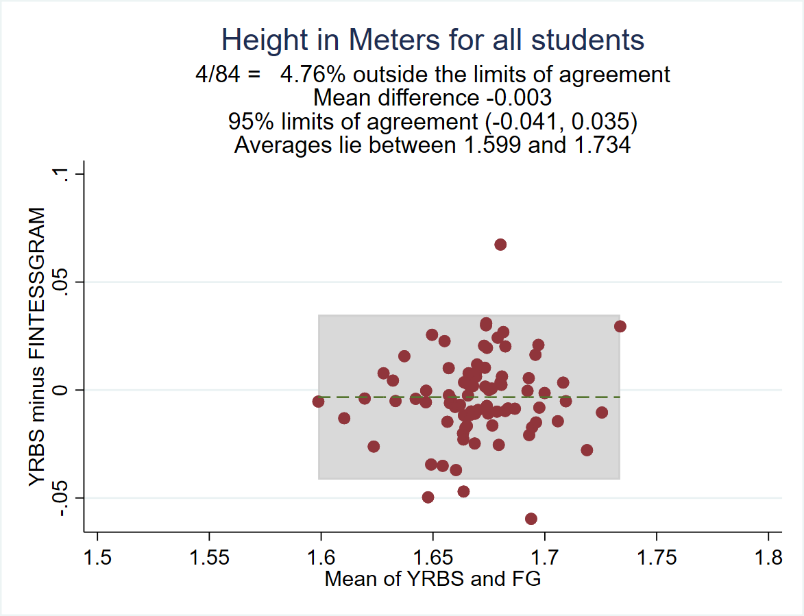

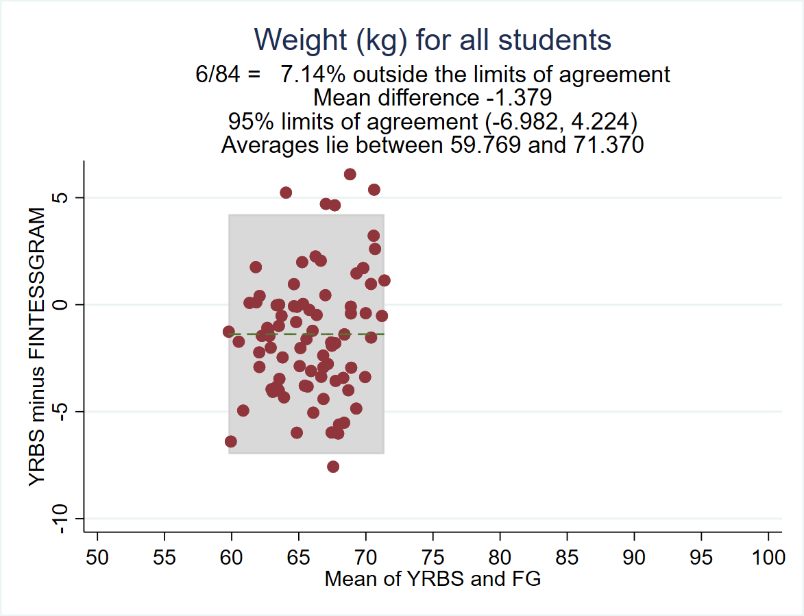
**

**
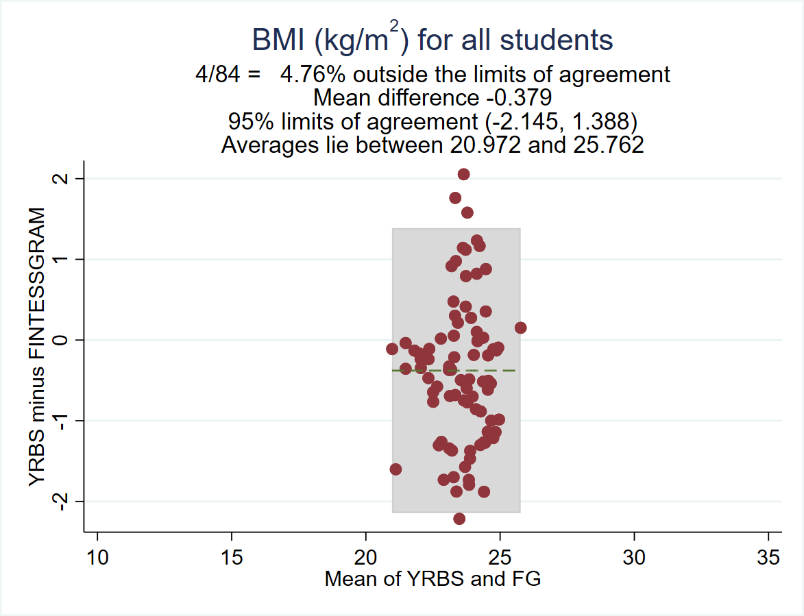
Figure 2: Bland-Altman Plots of height, weight, and BMI, for female students**

**
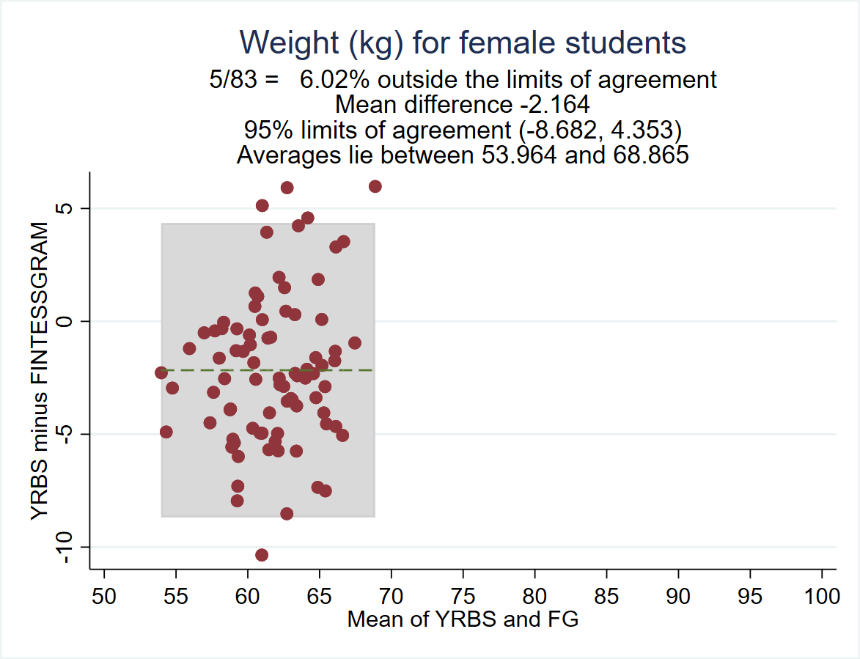

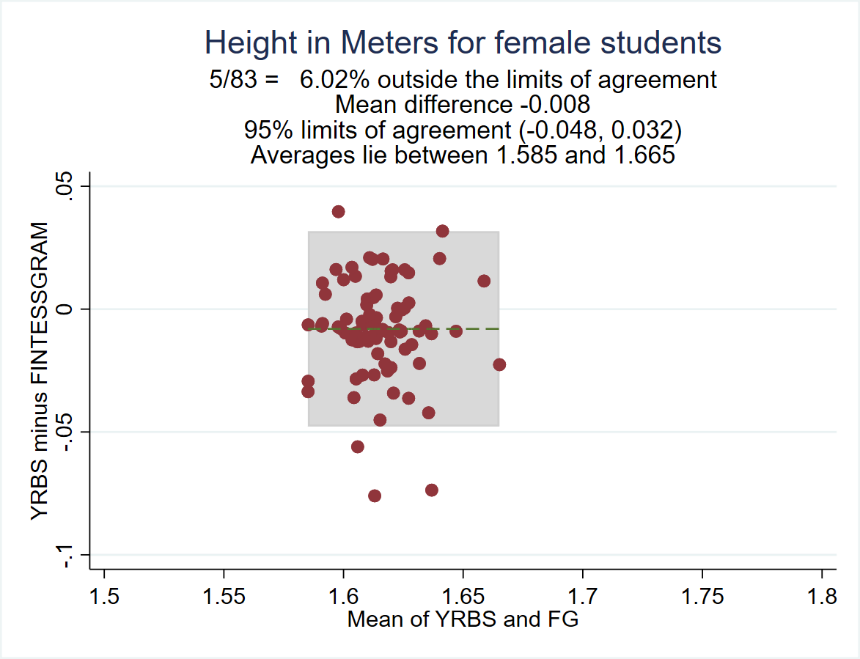
**

**
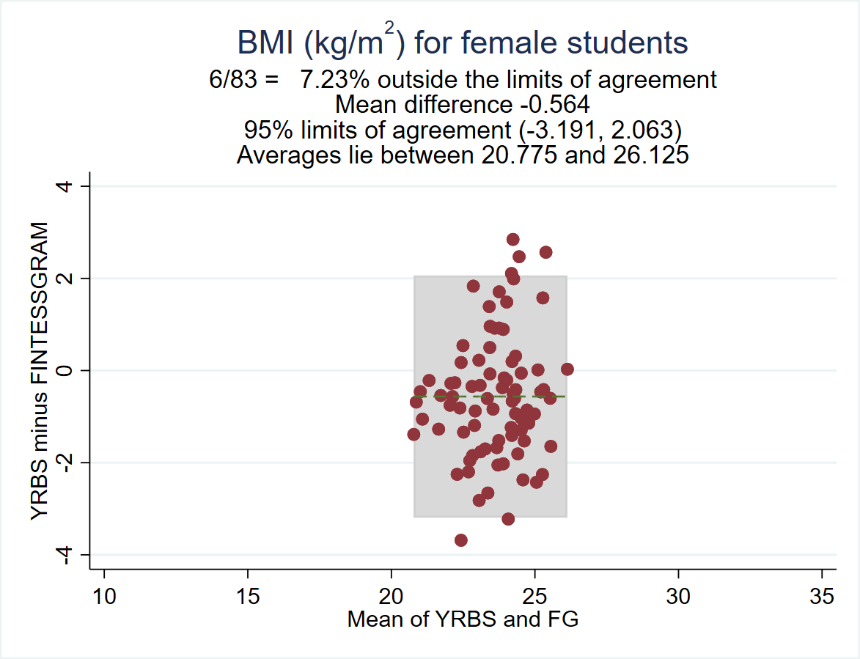
**

**Figure 3: Bland-Altman Plots of height, weight, and BMI, for male students**

**
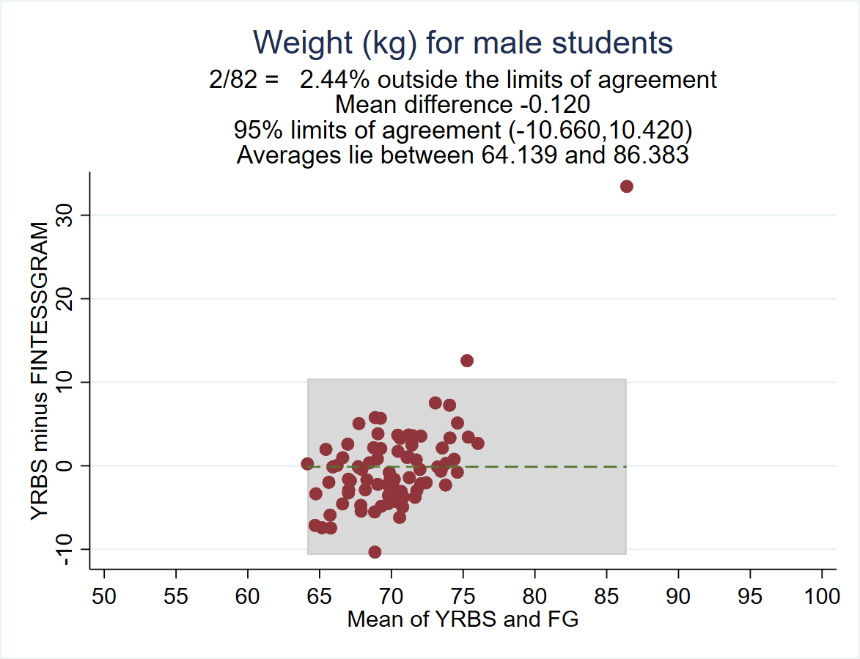

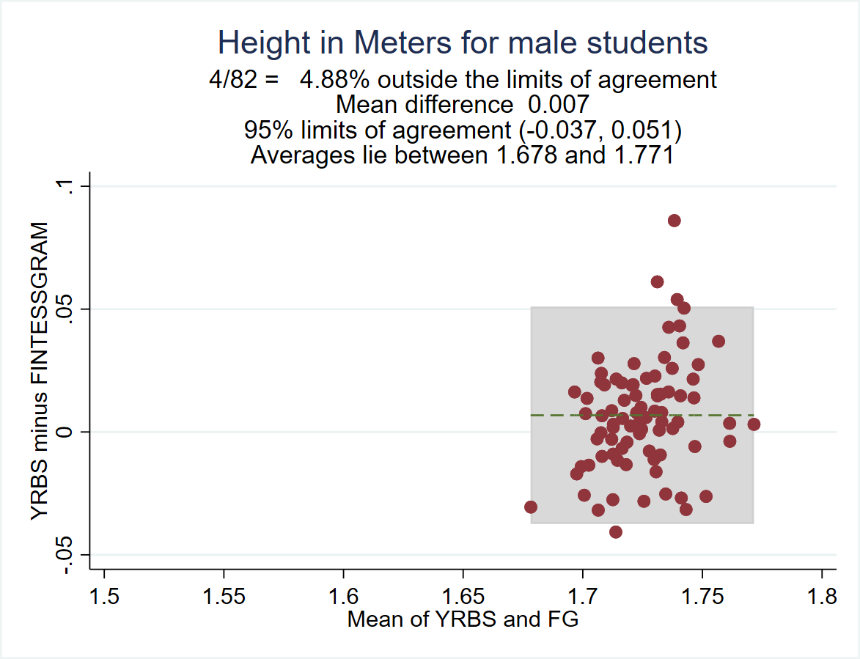
**

**
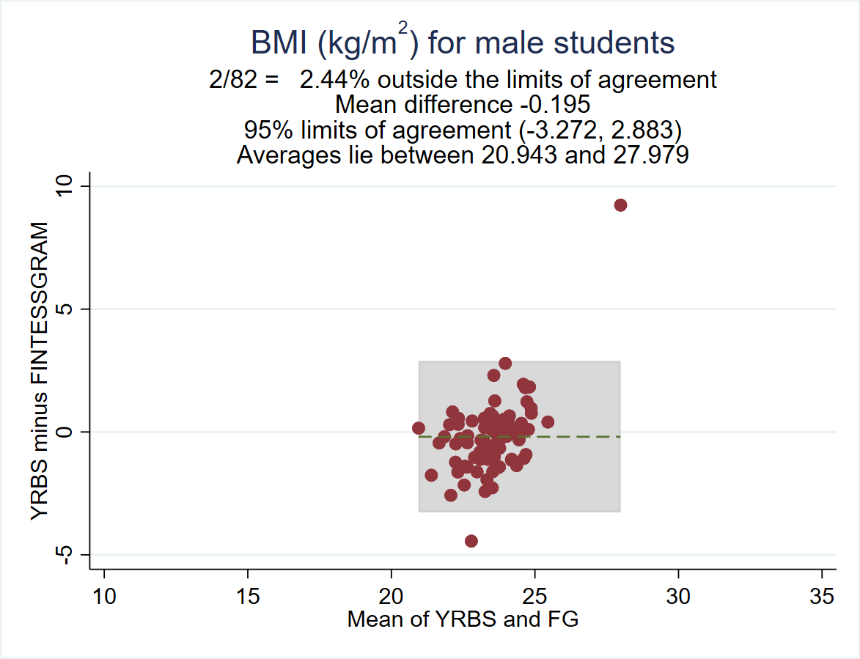
**

**Figure 4: Bland-Altman Plots of height, weight, and BMI, for students age 13**

**
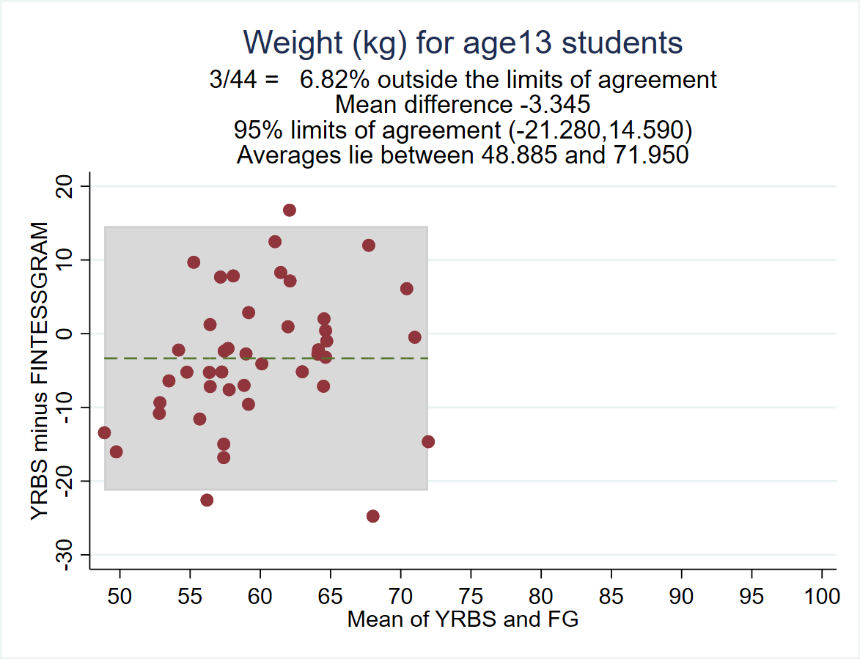

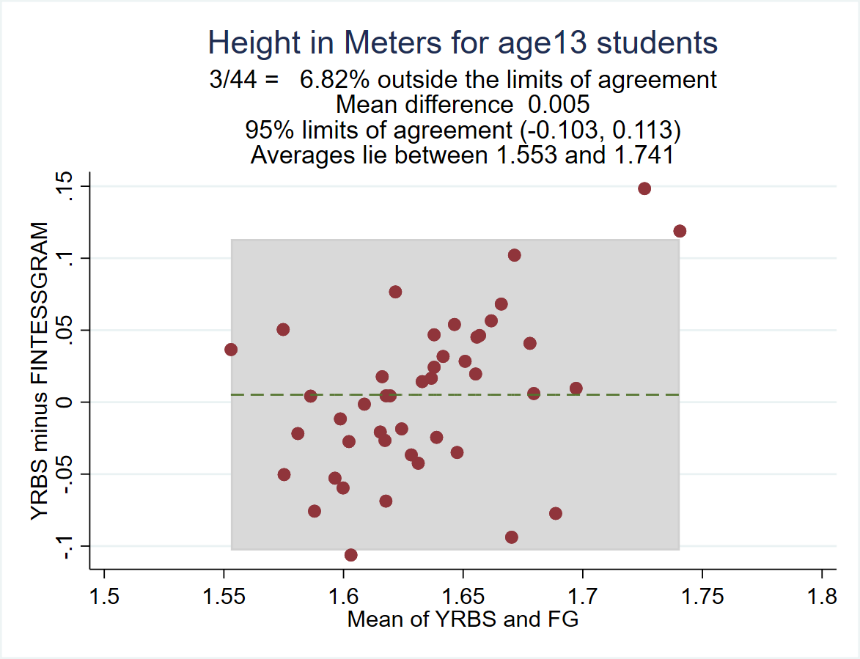
**

**
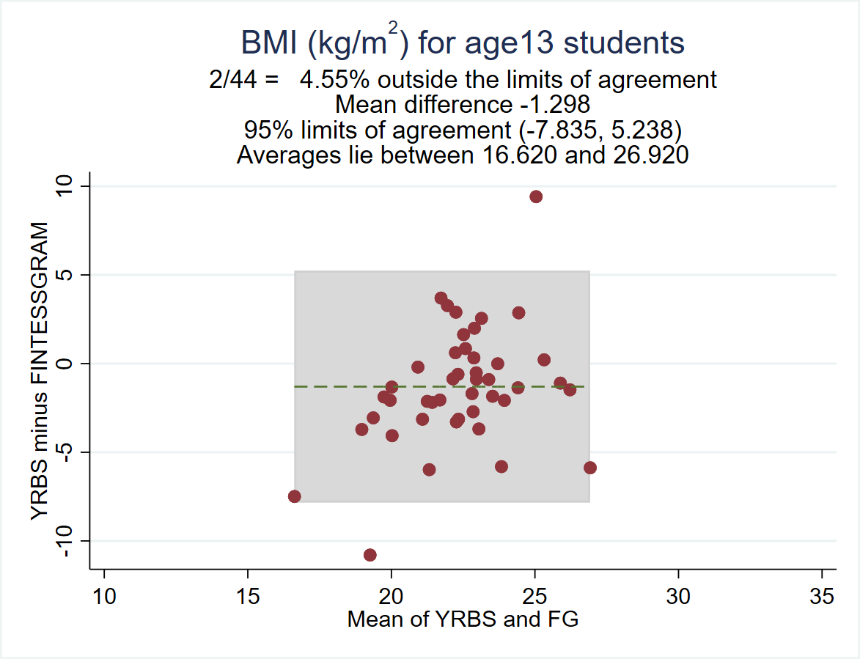
**

**Figure 5: Bland-Altman Plots of height, weight, and BMI, for students age 14**

**
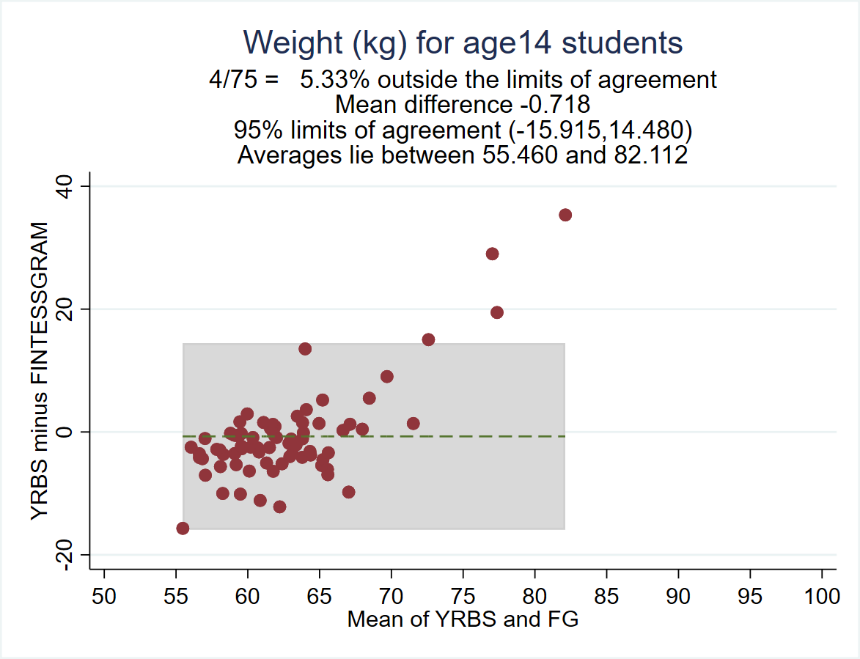

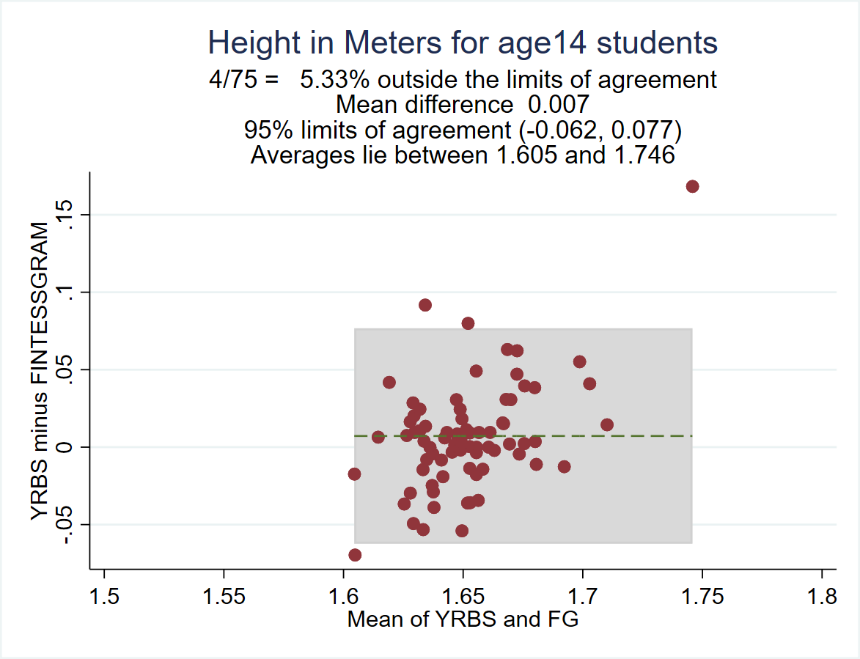
**

**
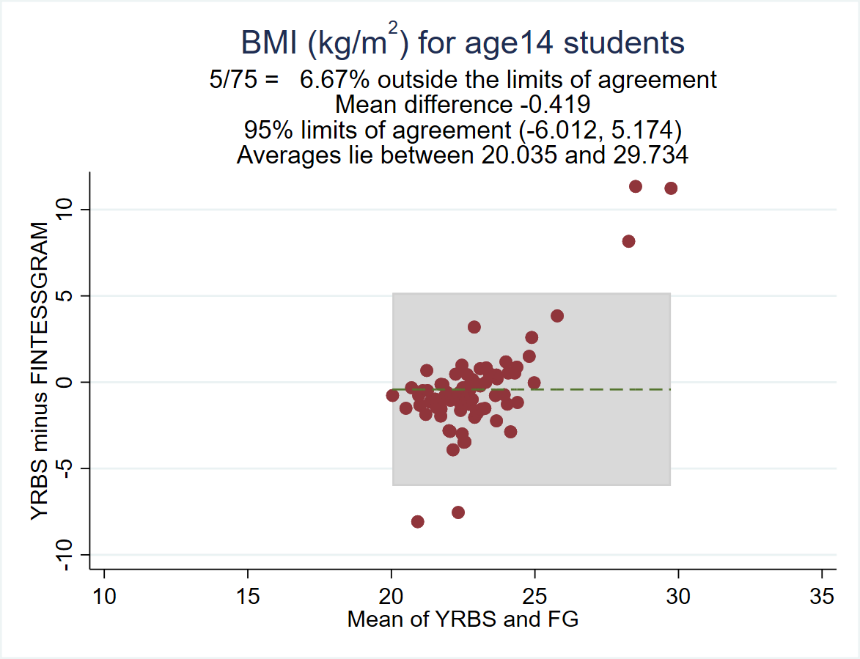
**

**Figure 6: Bland-Altman Plots of height, weight, and BMI, for students age 15**

**
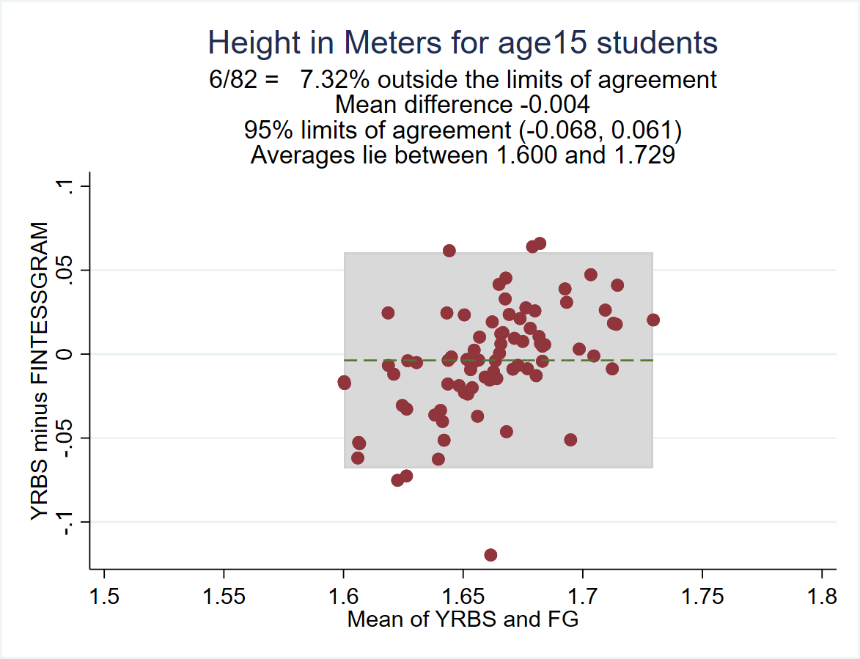

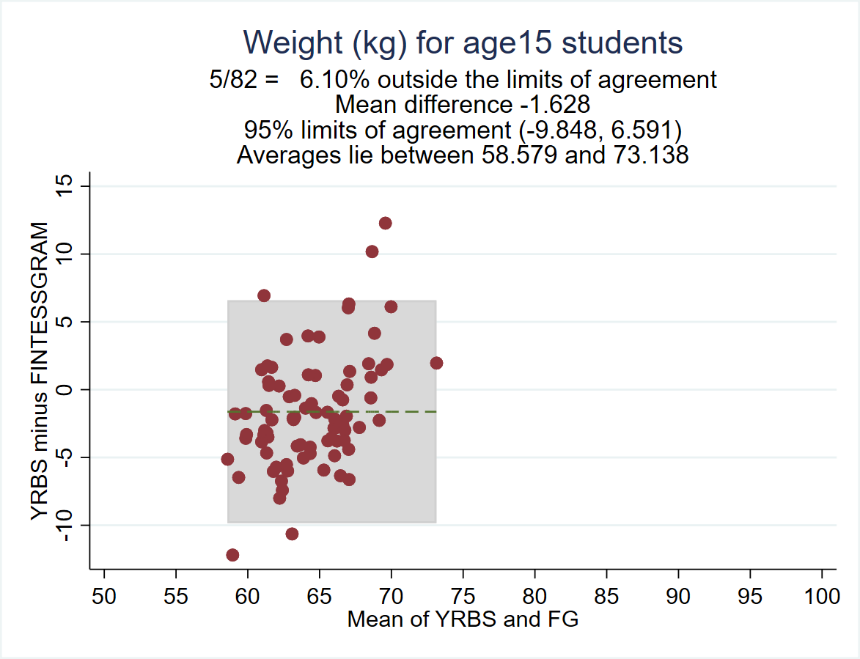
**

**
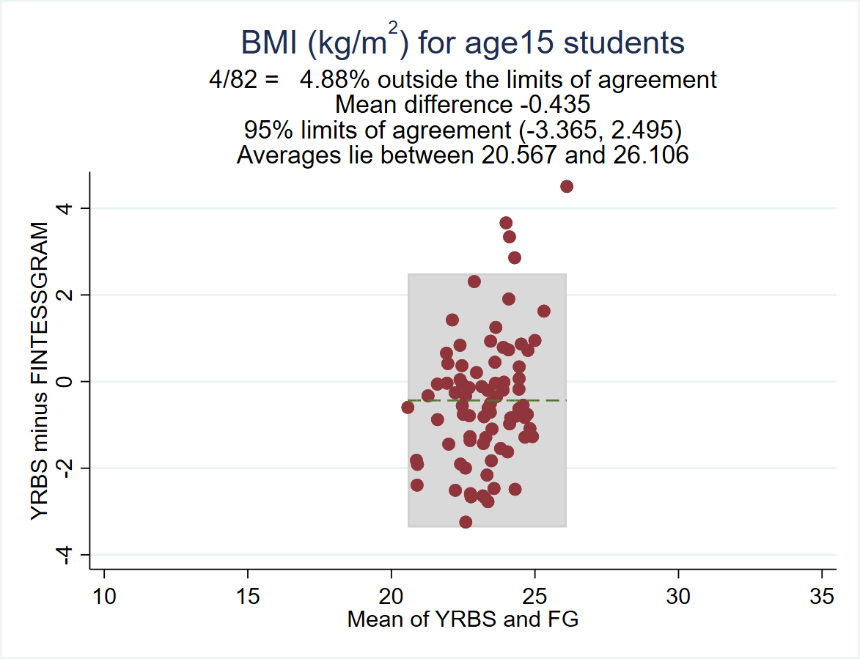
**

**Figure 7: Bland-Altman Plots of height, weight, and BMI, for students age 16**

**
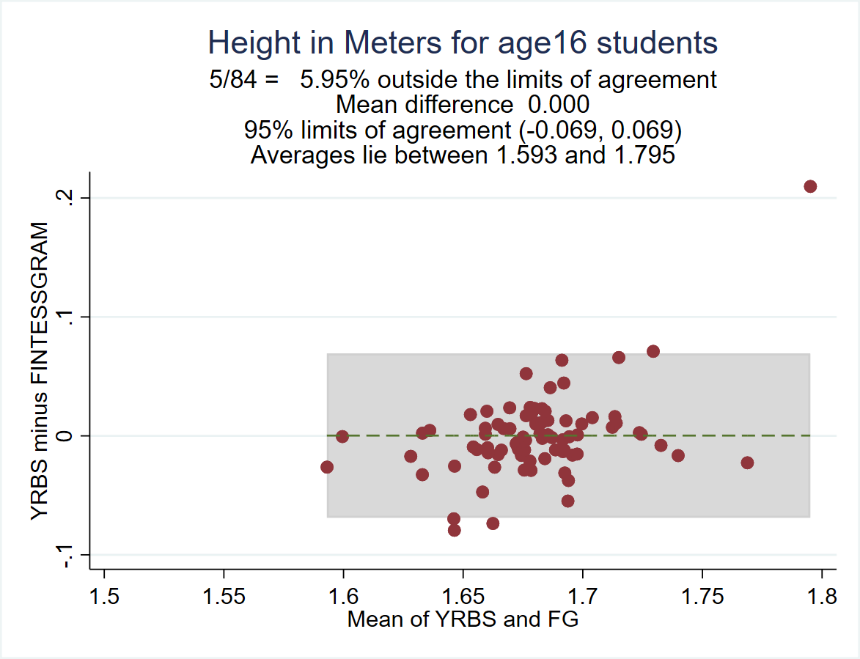

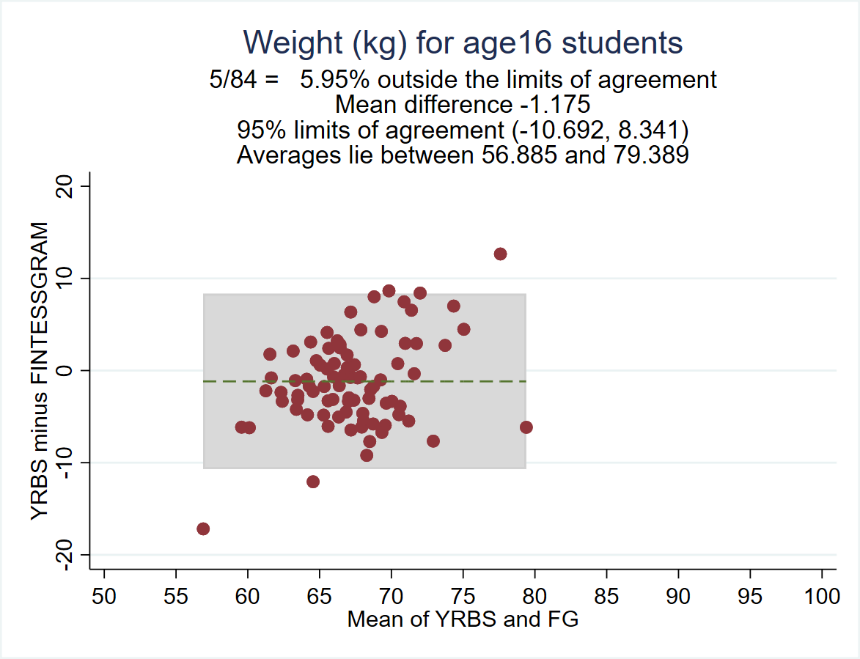
**

**
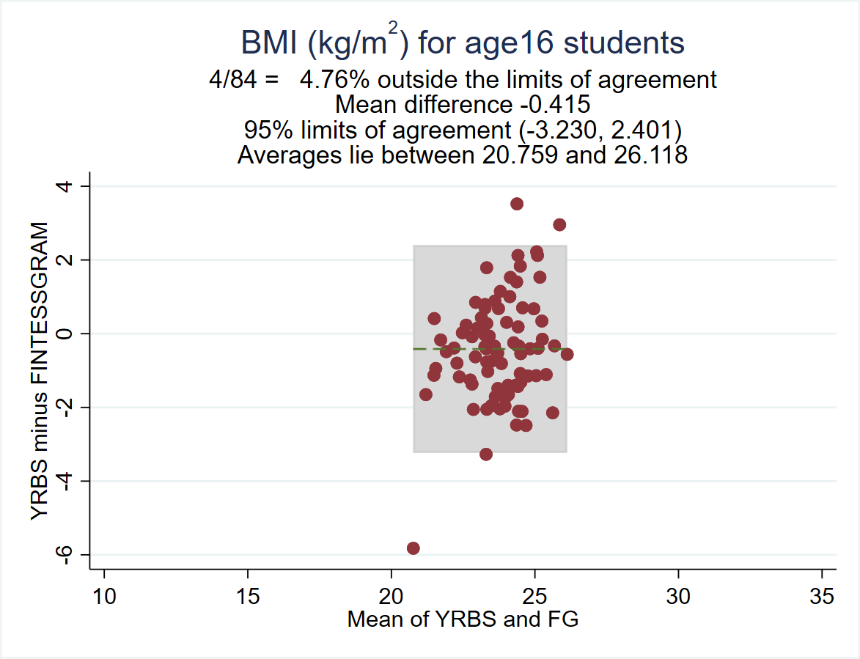
**

**Figure 8: Bland-Altman Plots of height, weight, and BMI, for students age 17**

**
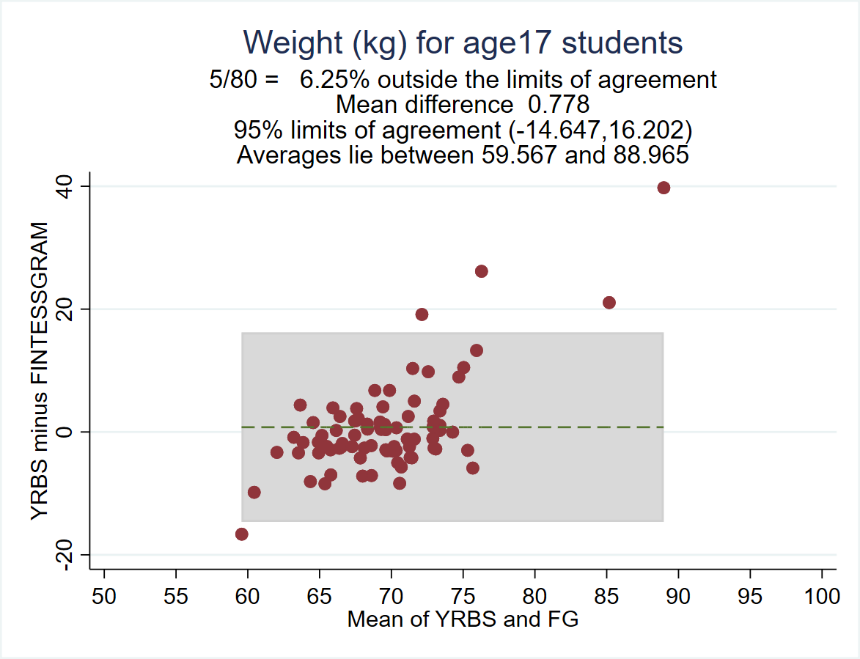

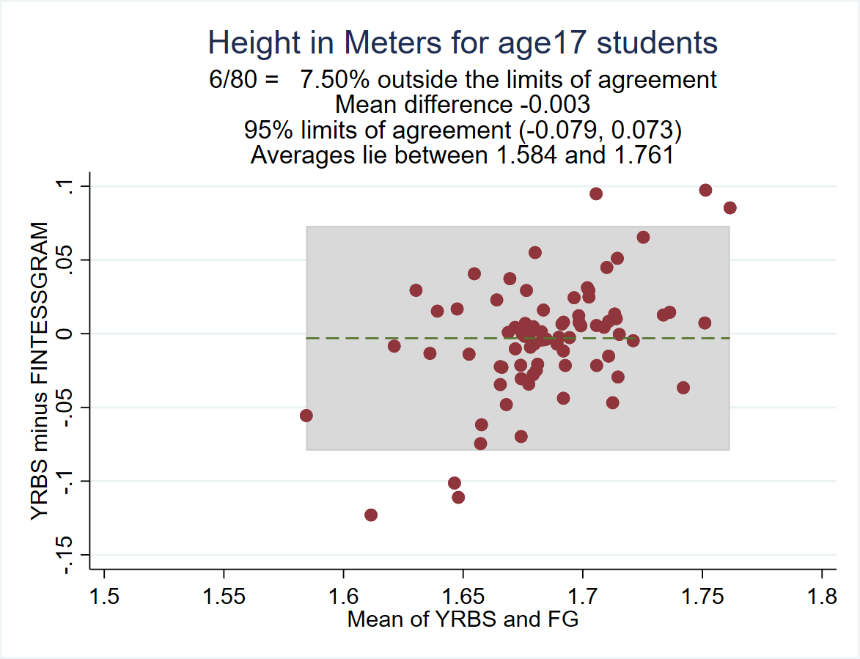
**

**
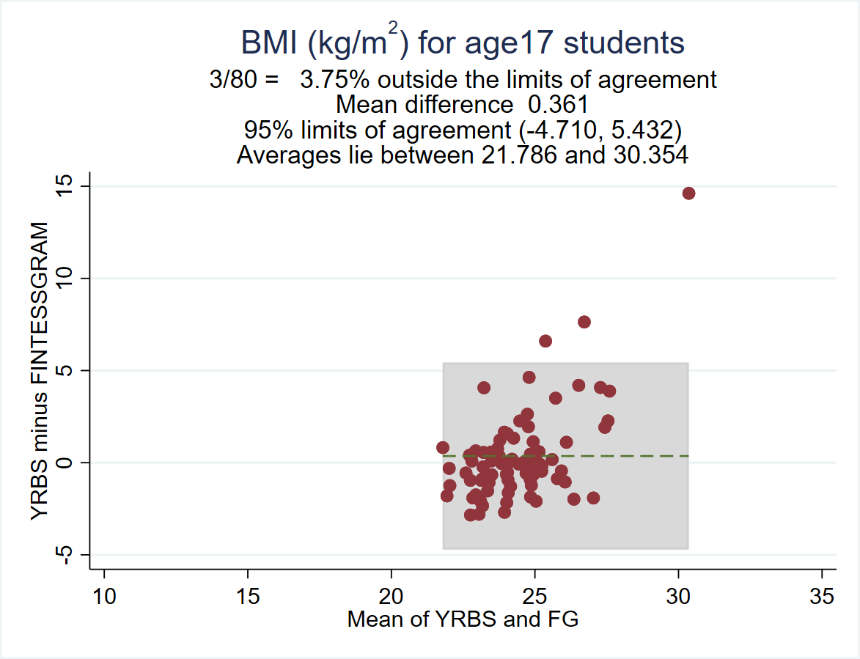
**

**Figure 9: Bland-Altman Plots of height, weight, and BMI, for students age 18**

**
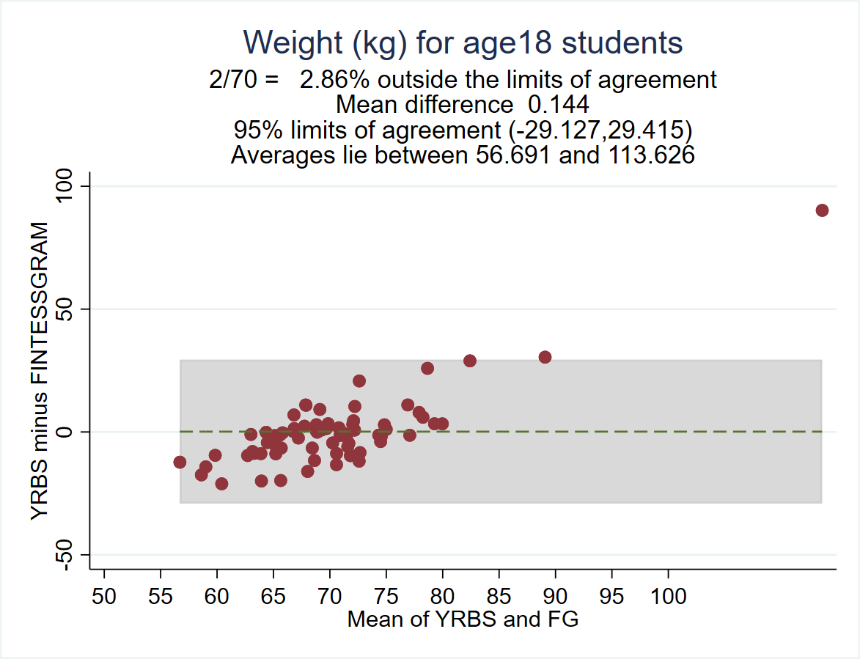

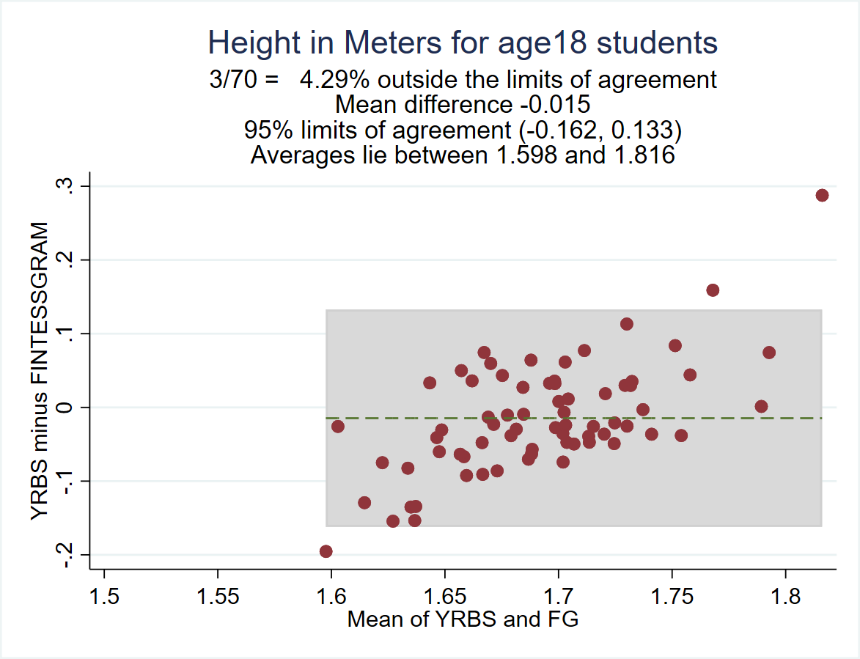
**

**
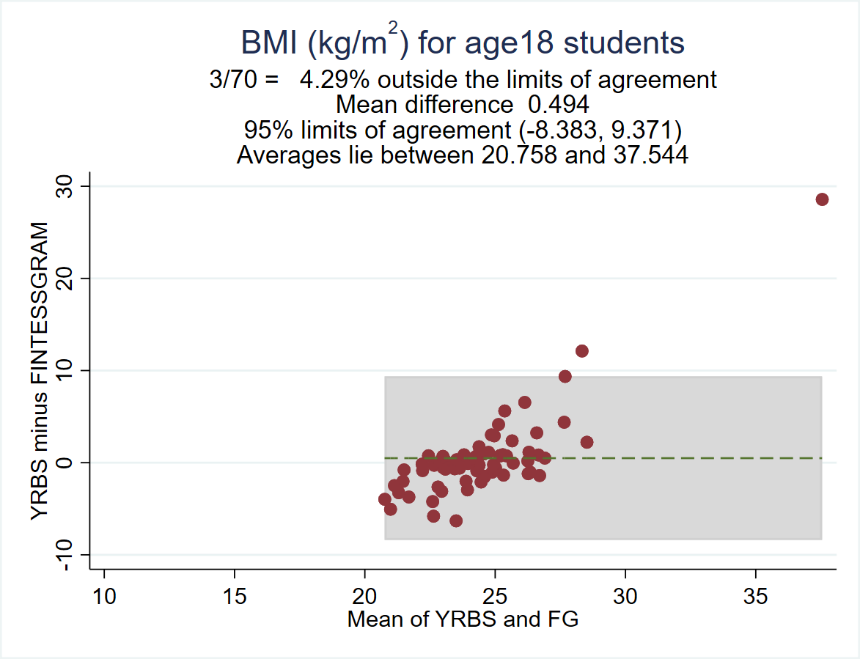
**

**Figure 10: Bland-Altman Plots of height, weight, and BMI, for American Indian/Alaska Native students**

**
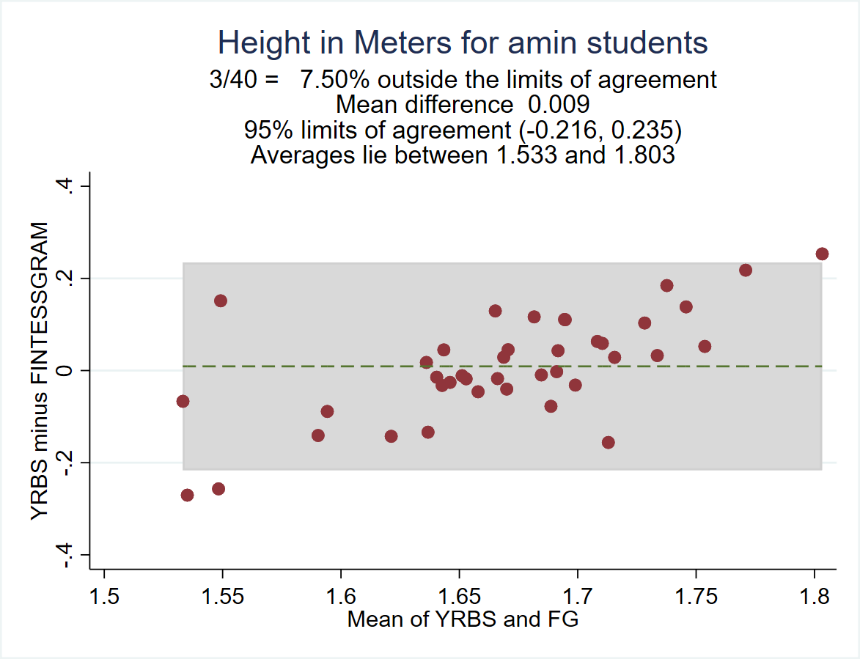

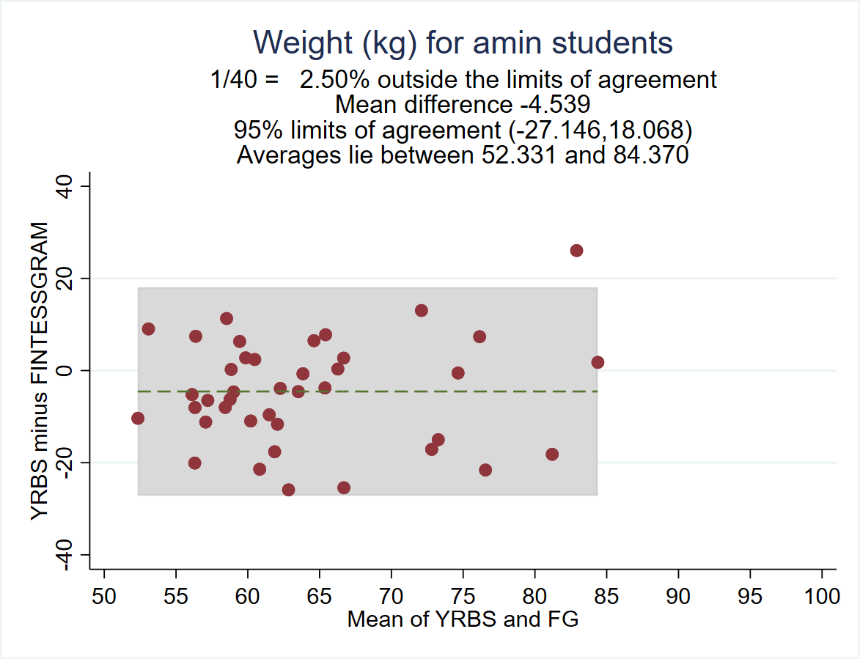
**

**
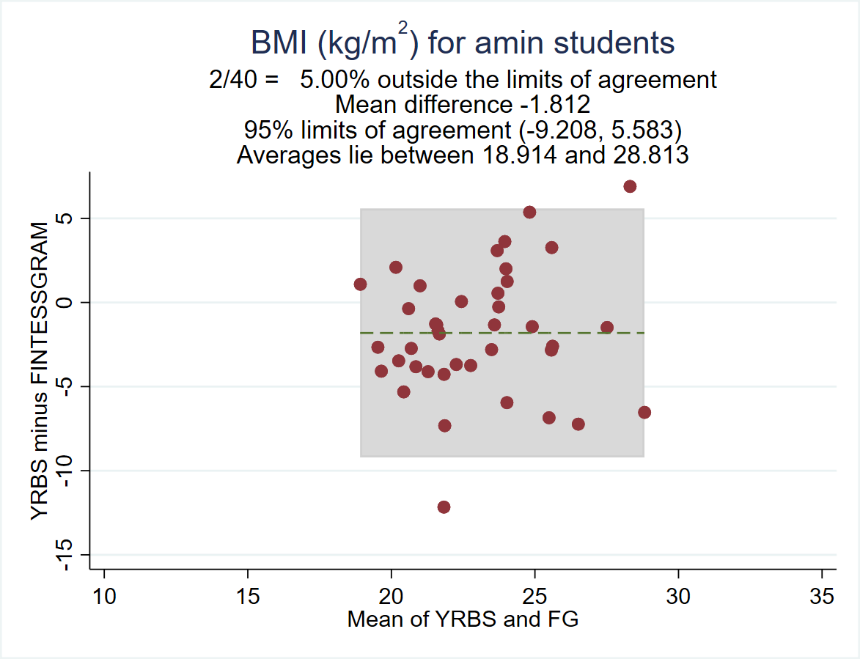
**

**Figure 11: Bland-Altman Plots of height, weight, and BMI, for Asian/Pacific Islander students**

**
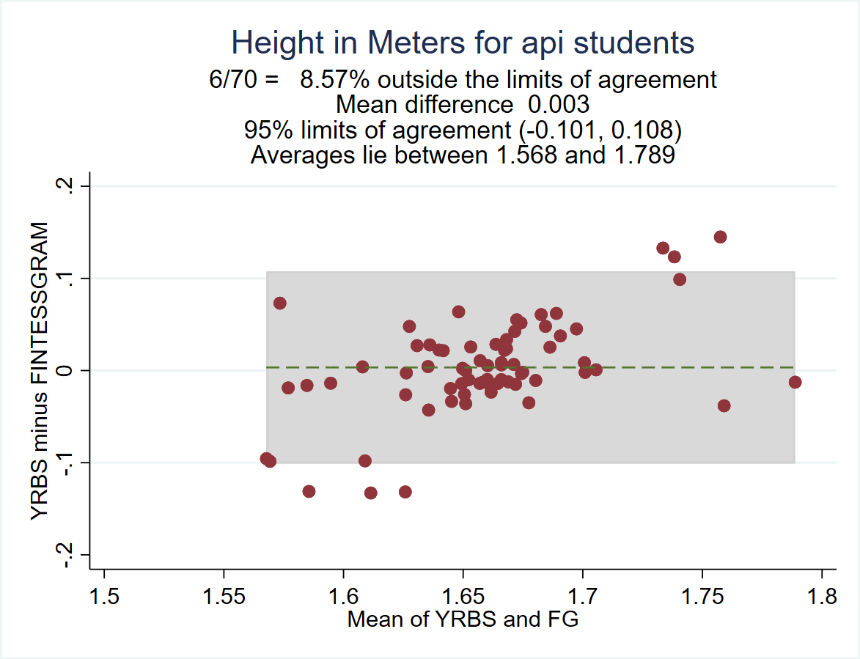

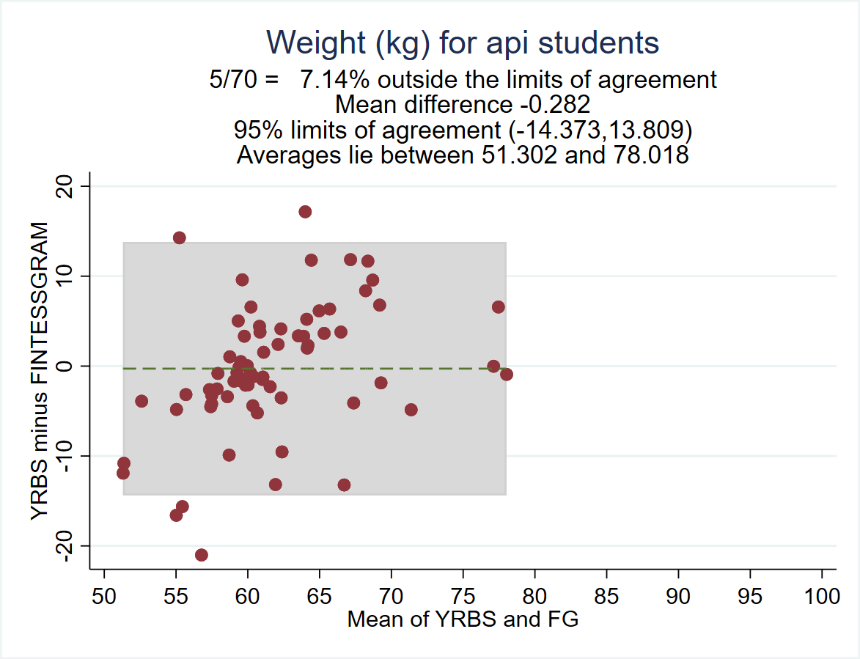
**

**
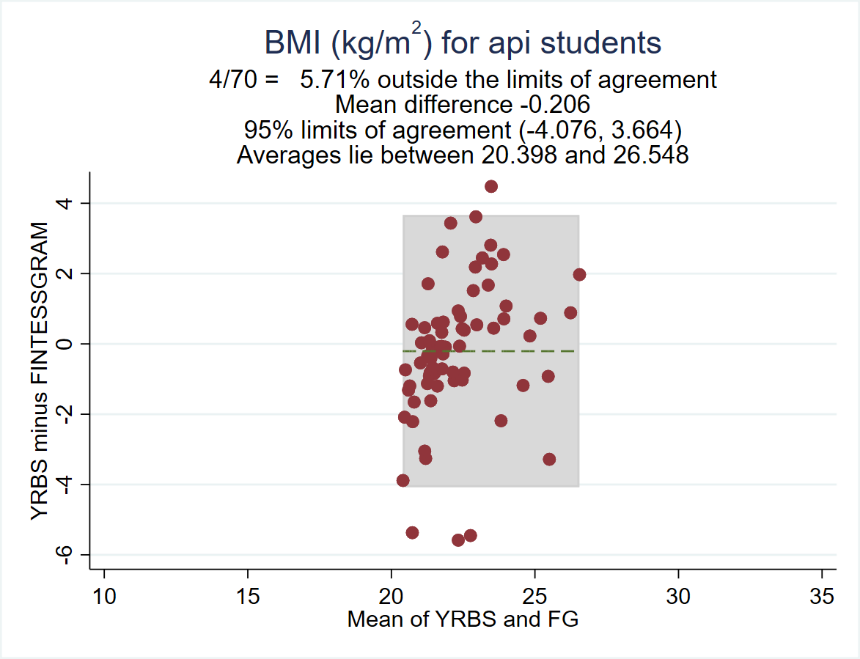
**

**Figure 12: Bland-Altman Plots of height, weight, and BMI, for Non-Hispanic Black**

**
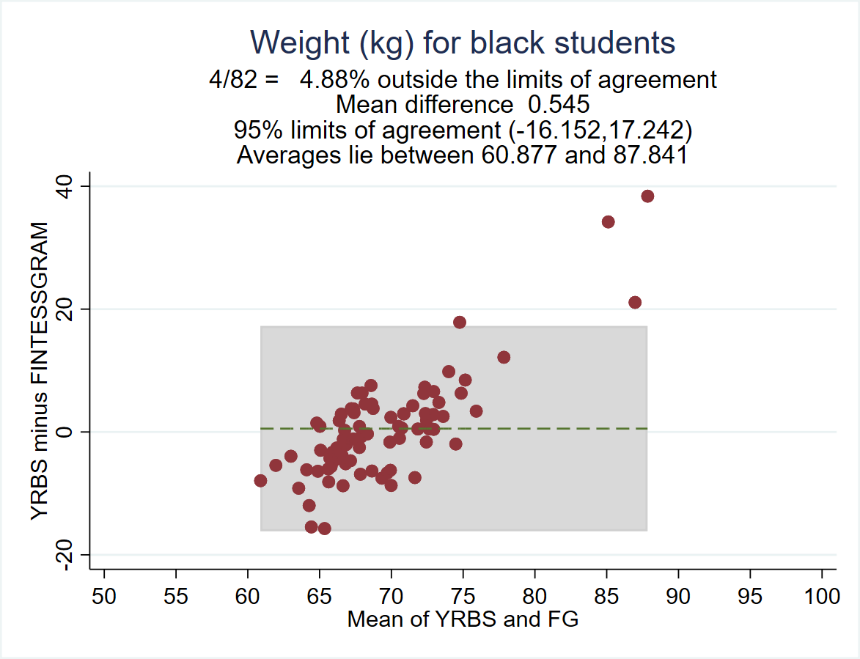

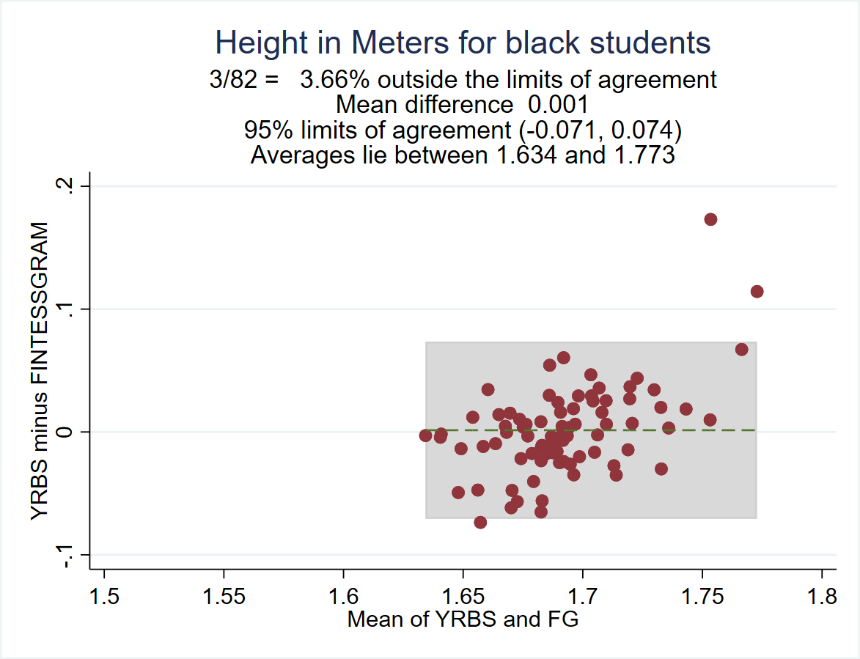
**

**
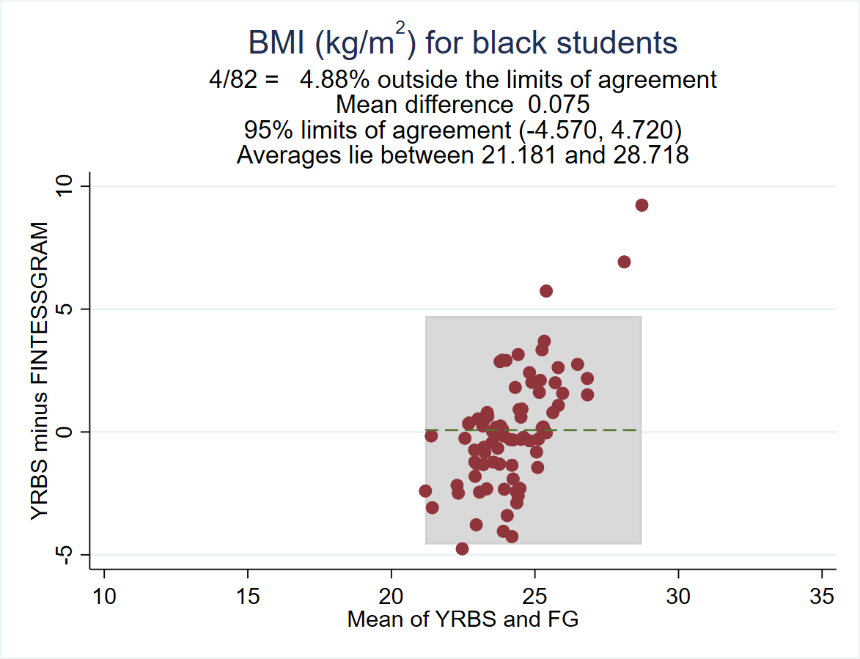
**

**Figure 13: Bland-Altman Plots of height, weight, and BMI, for Native Hawaiian students**

**
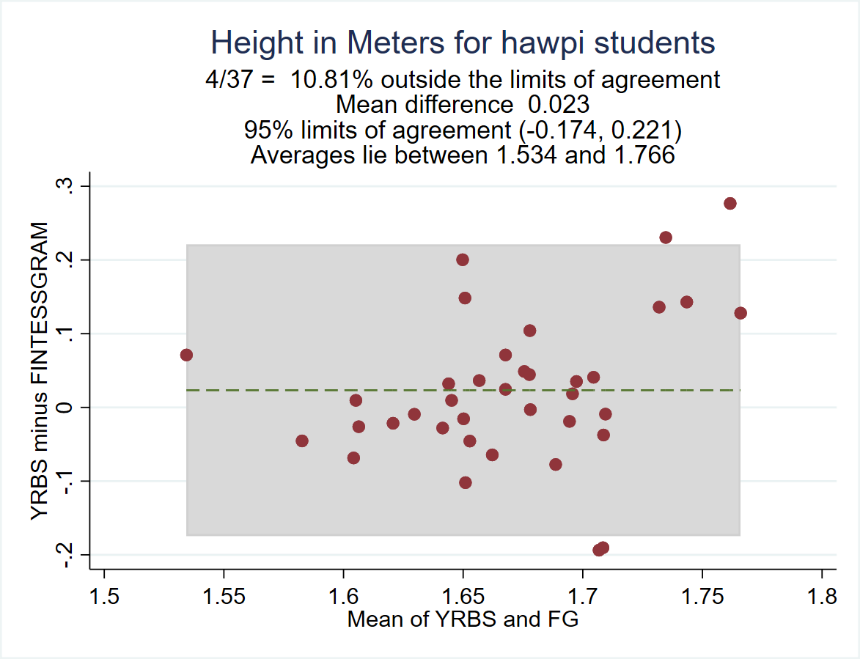

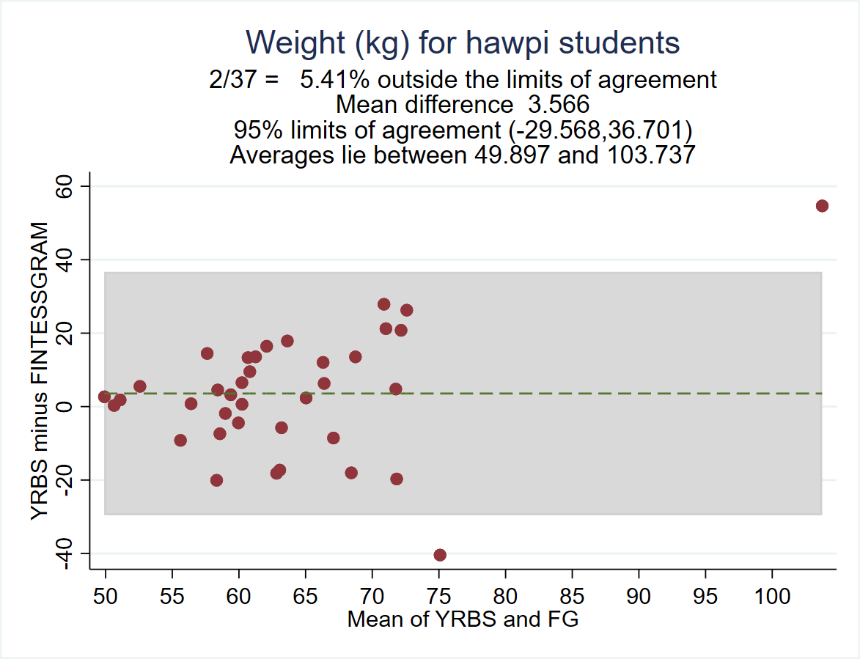
**

**
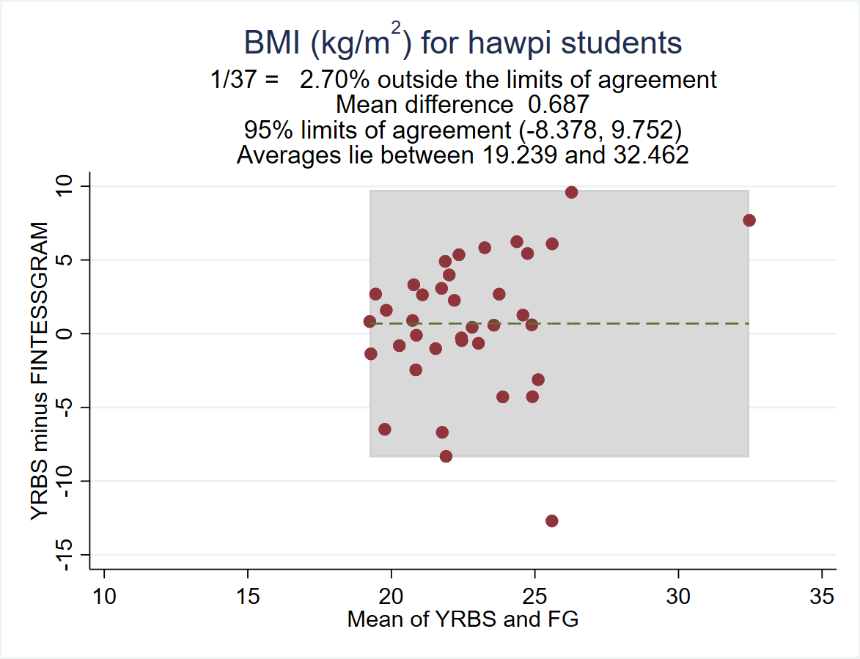
**

**Figure 14: Bland-Altman Plots of height, weight, and BMI, for White students**

**
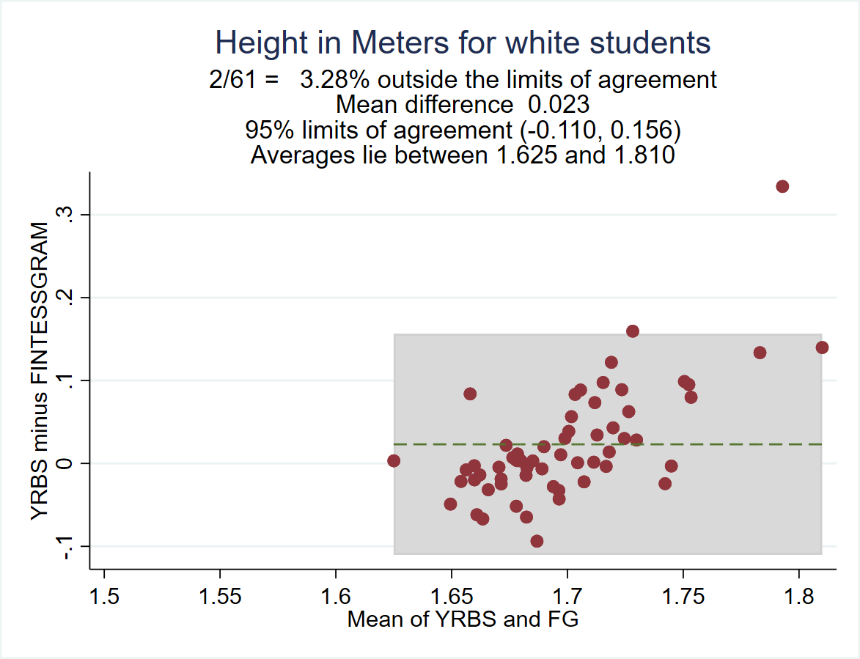

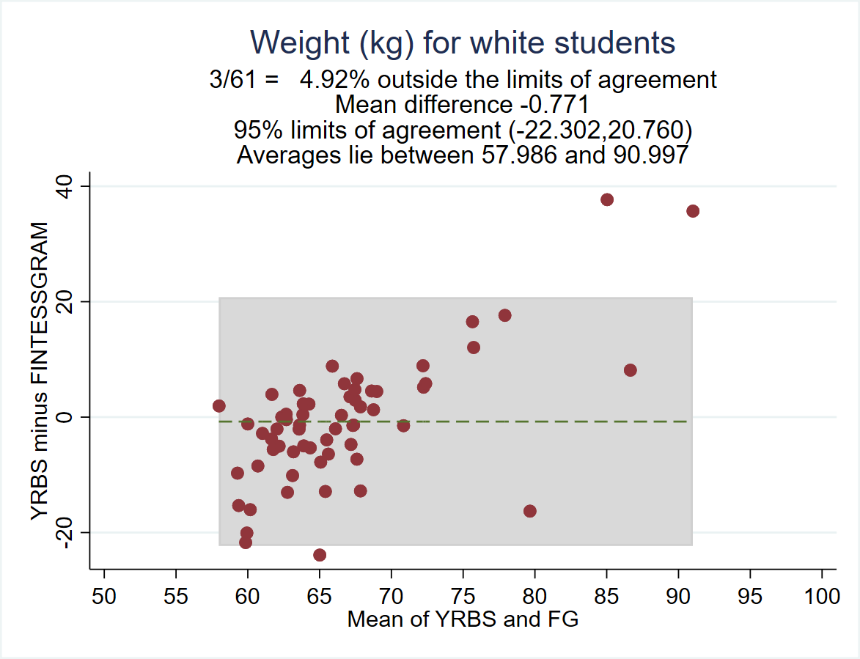
**

**
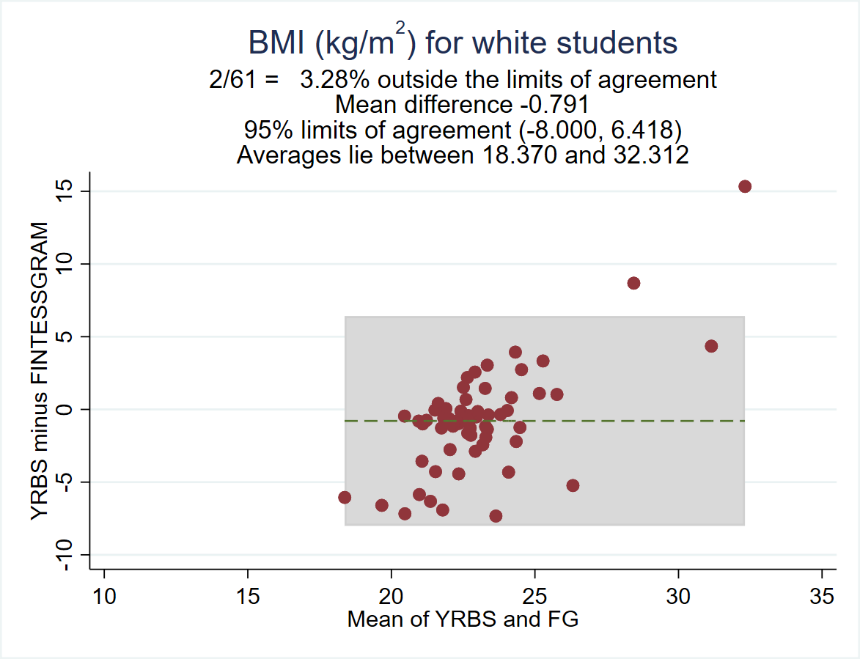
**

**Figure 15: Bland-Altman Plots of height, weight, and BMI, for Hispanic Latino students**

**
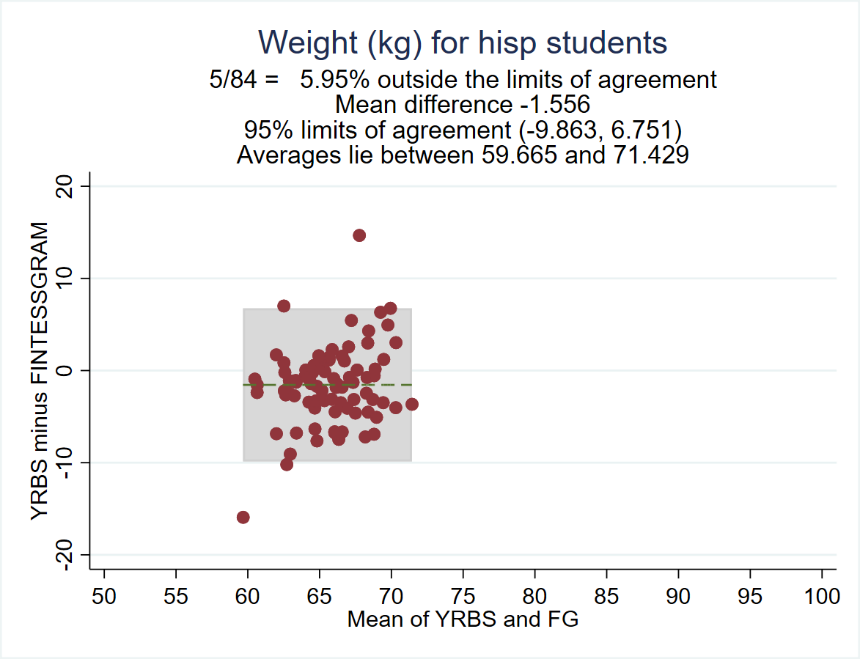

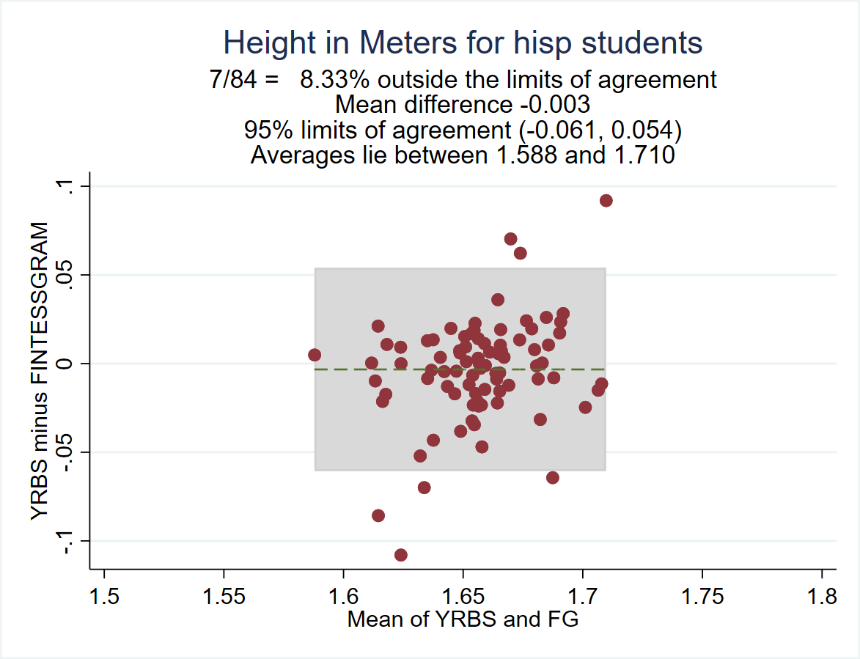
**

**
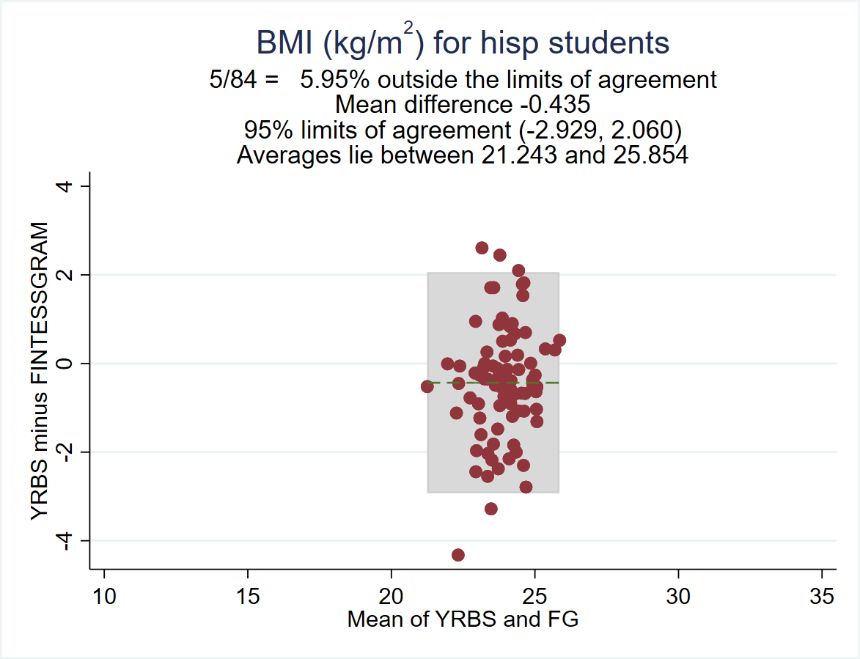
**

**Figure 16: Bland-Altman Plots of height, weight, and BMI, for Multi-racial students**

**
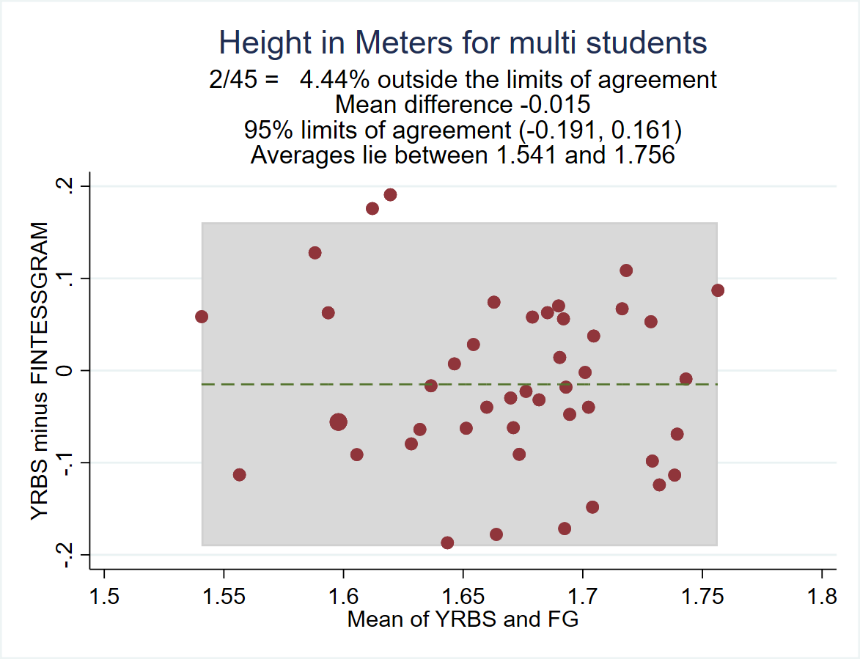

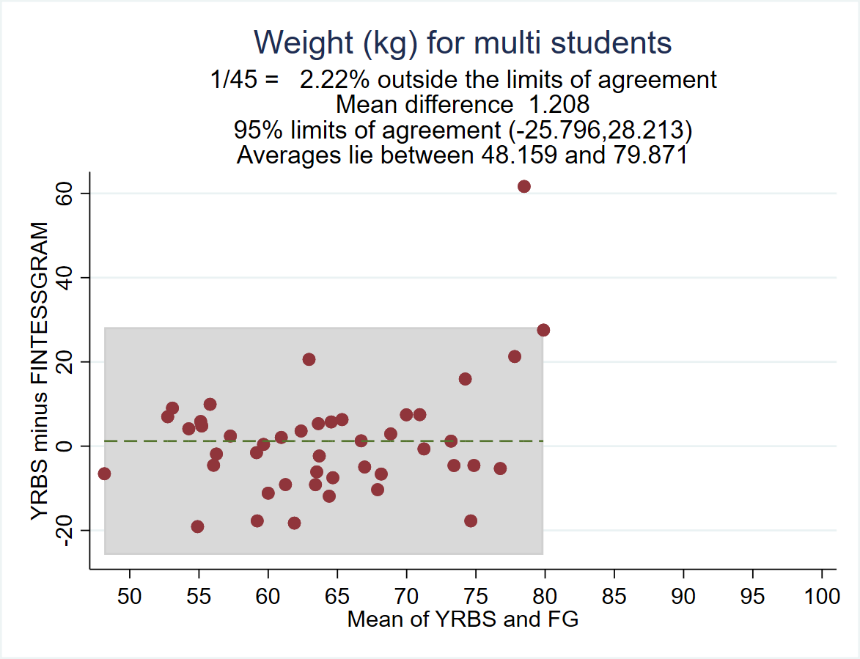
**

**
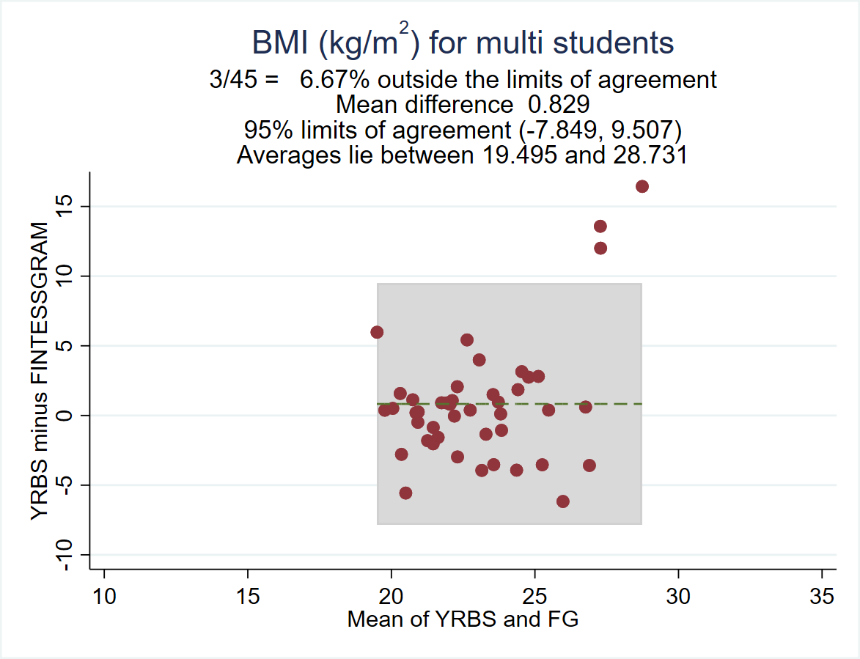
**

**Figure 17: Bland-Altman Plots of height, weight, and BMI, for Age 13 females and Age 13 males – all students**


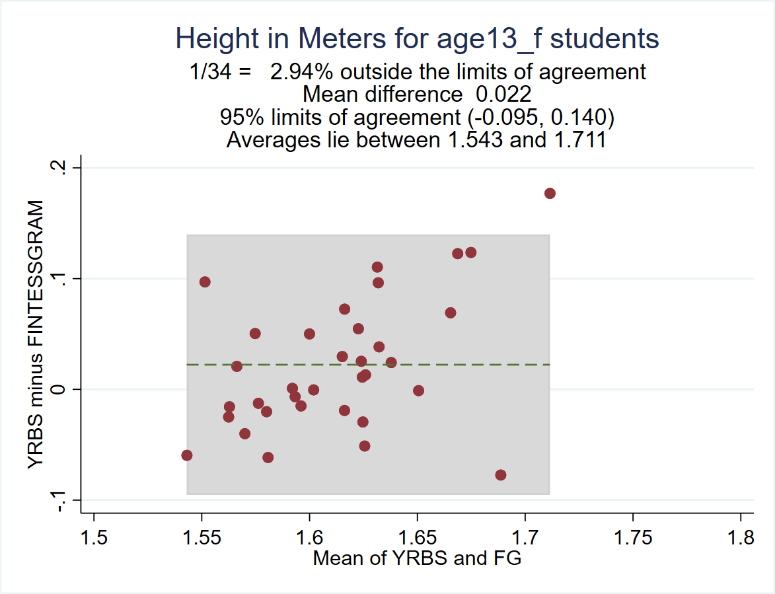

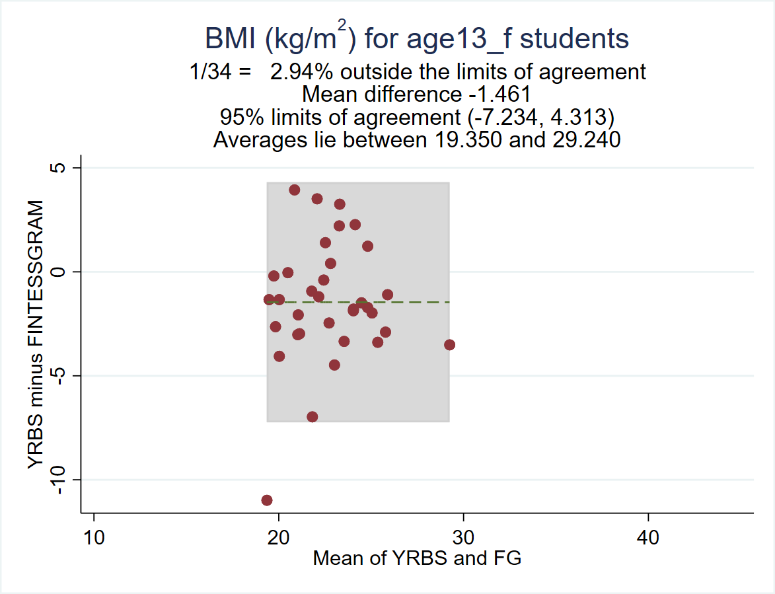

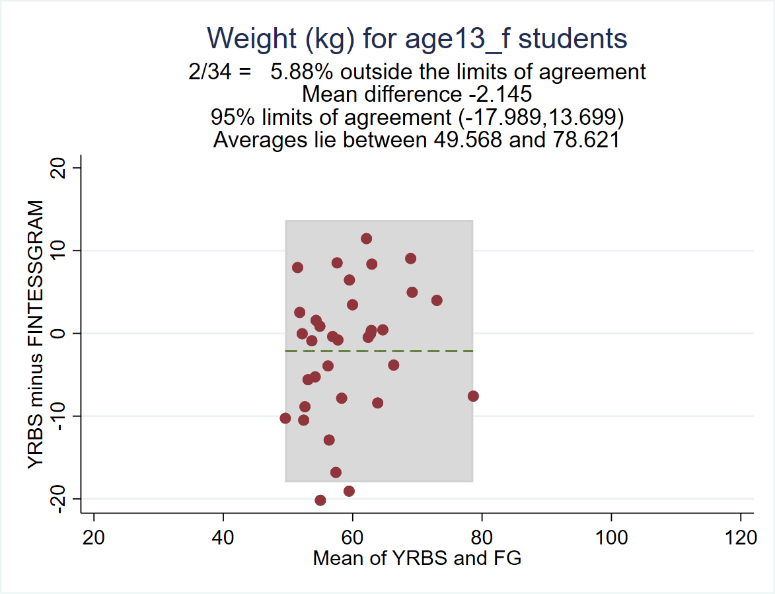

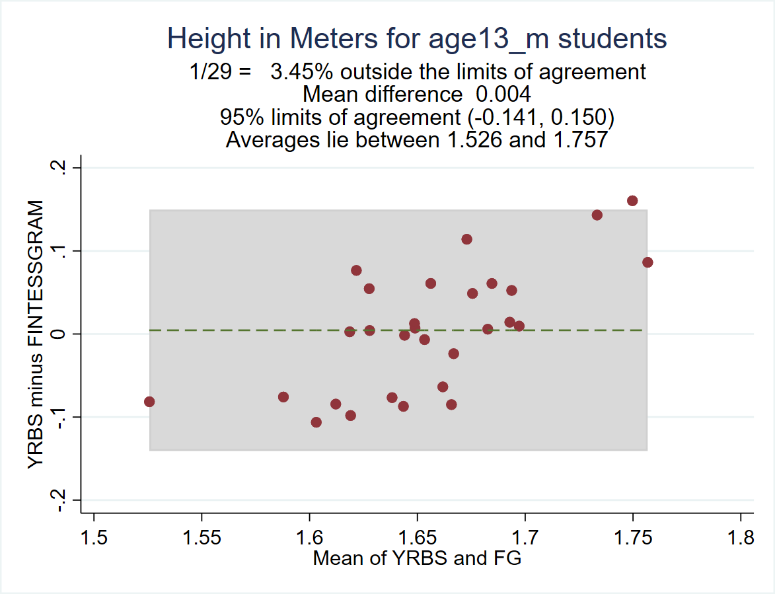

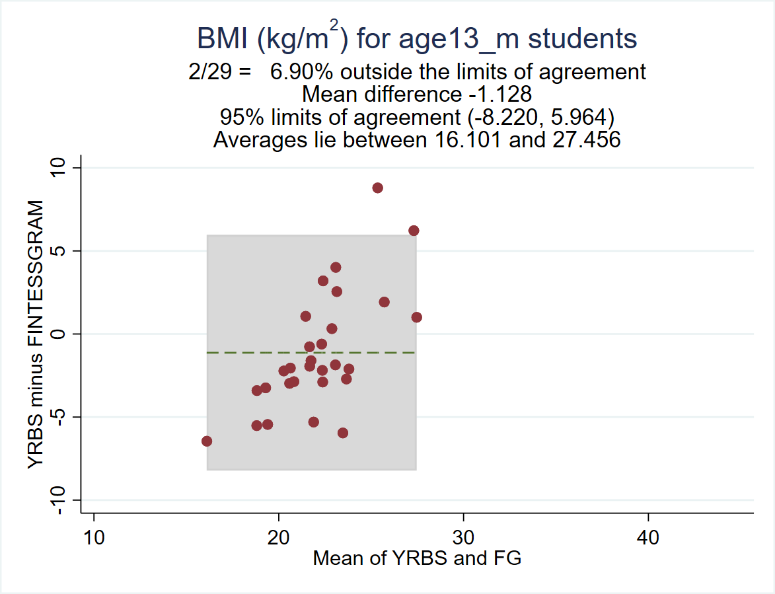

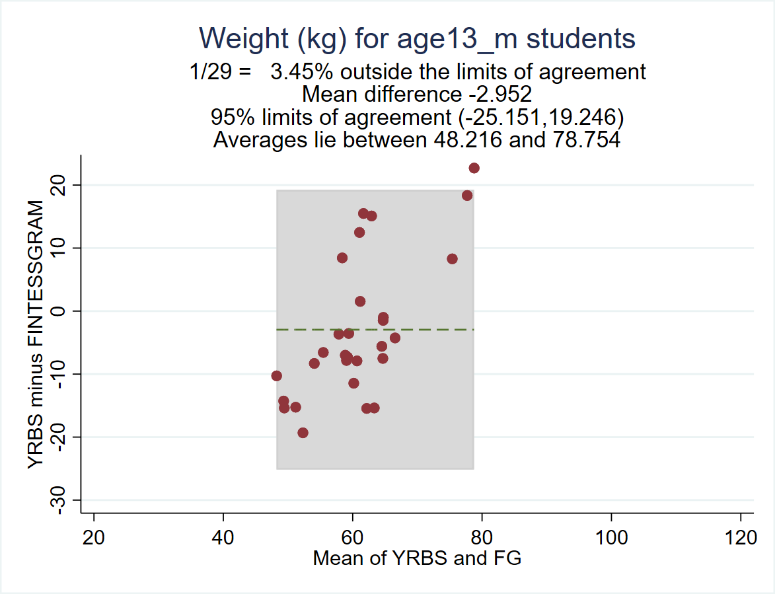


**Figure 18: Bland-Altman Plots of height, weight, and BMI, for Age 14 females and Age 14 males – all students**


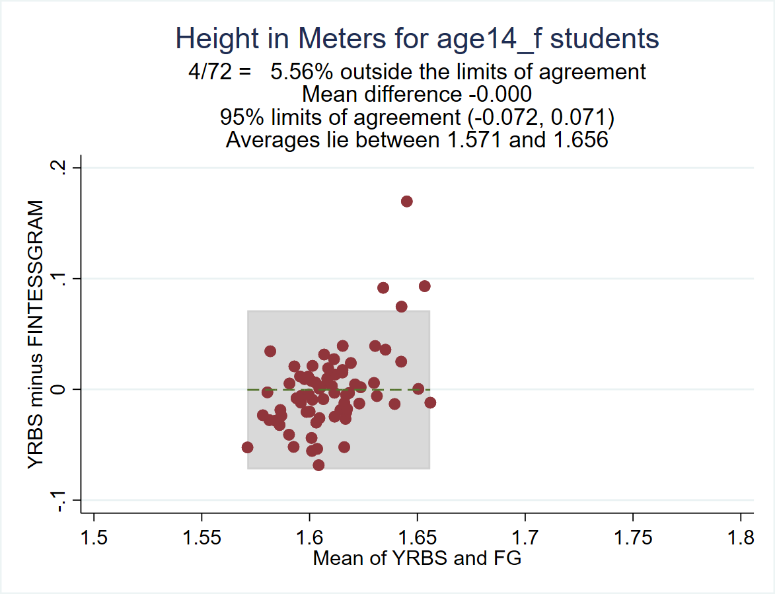

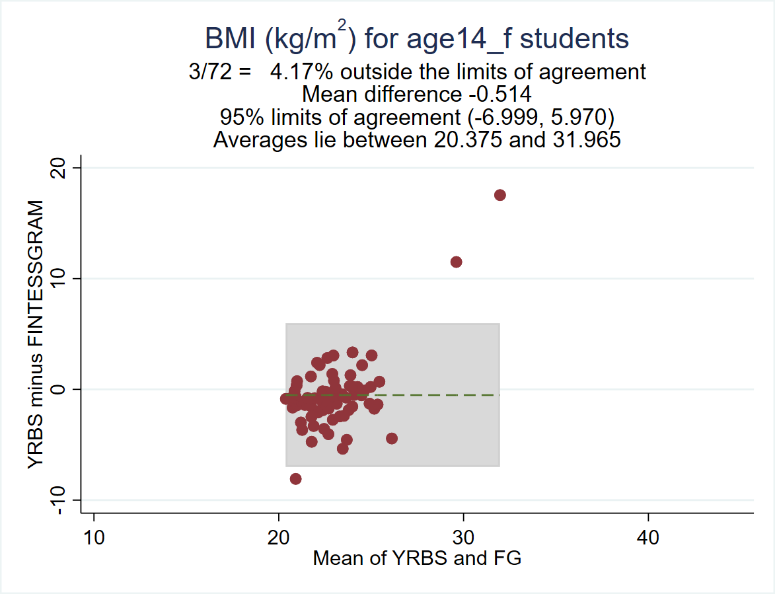

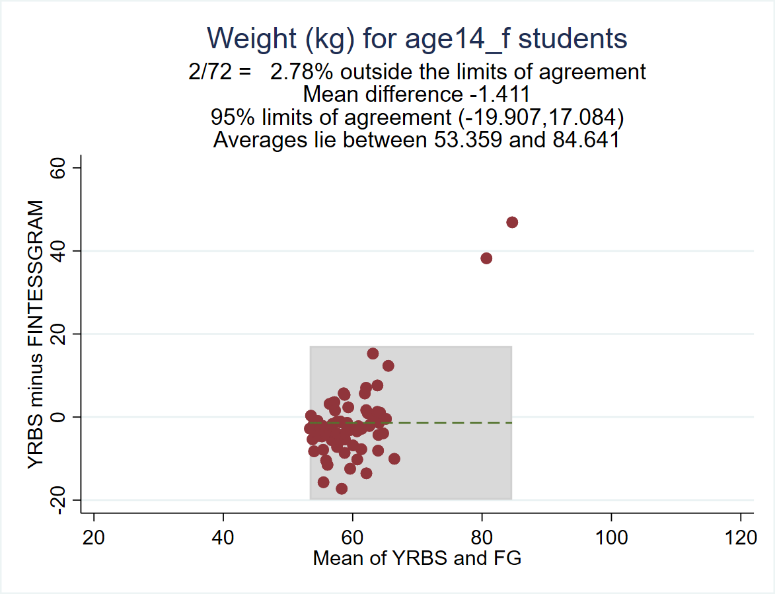

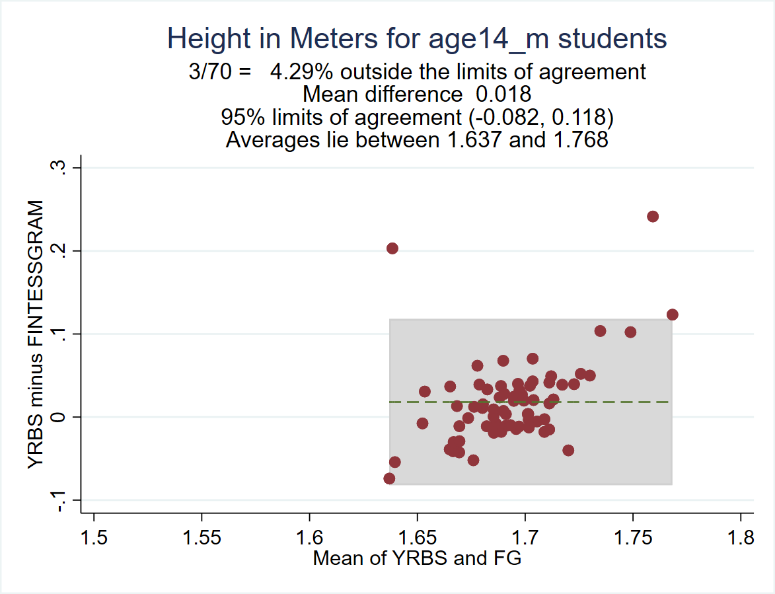

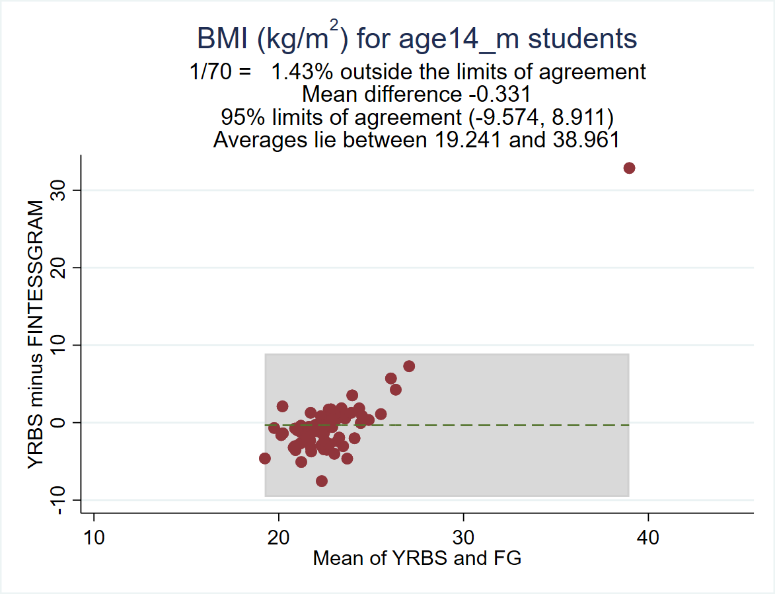

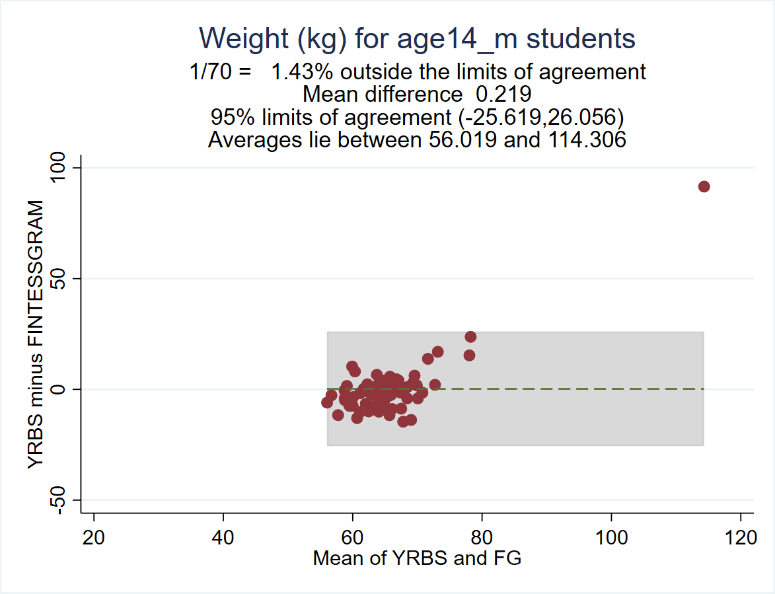


**Figure 19: Bland-Altman Plots of height, weight, and BMI, for Age 15 females and Age 15 males – all students**


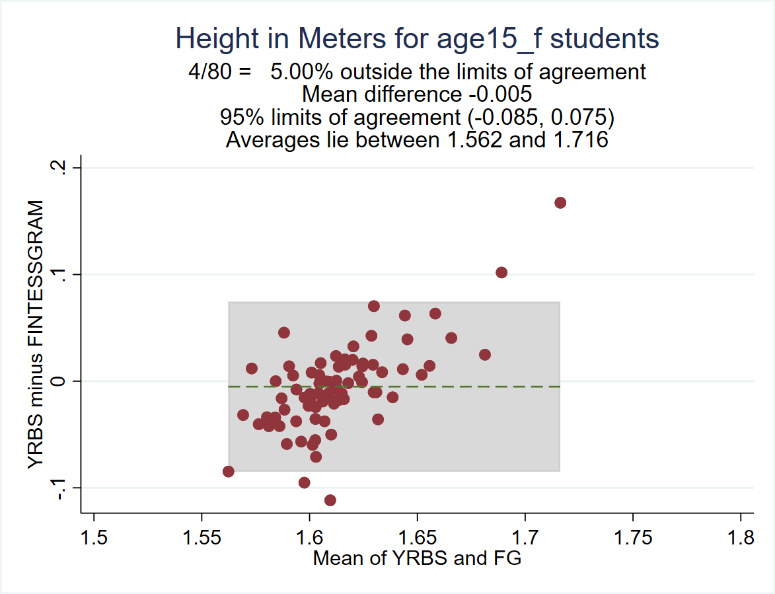

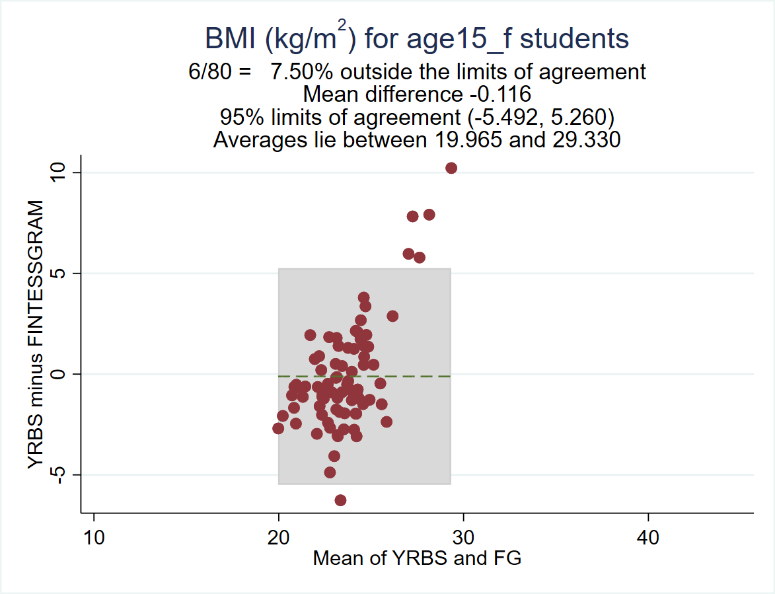

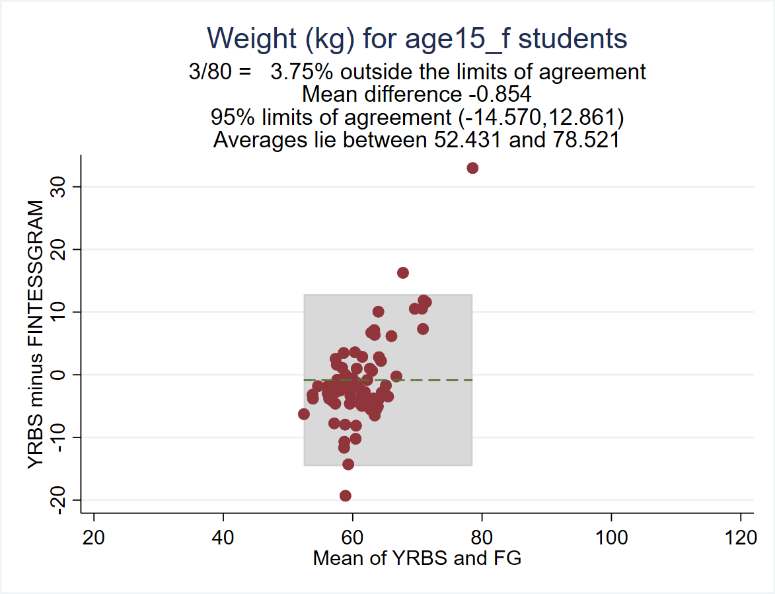

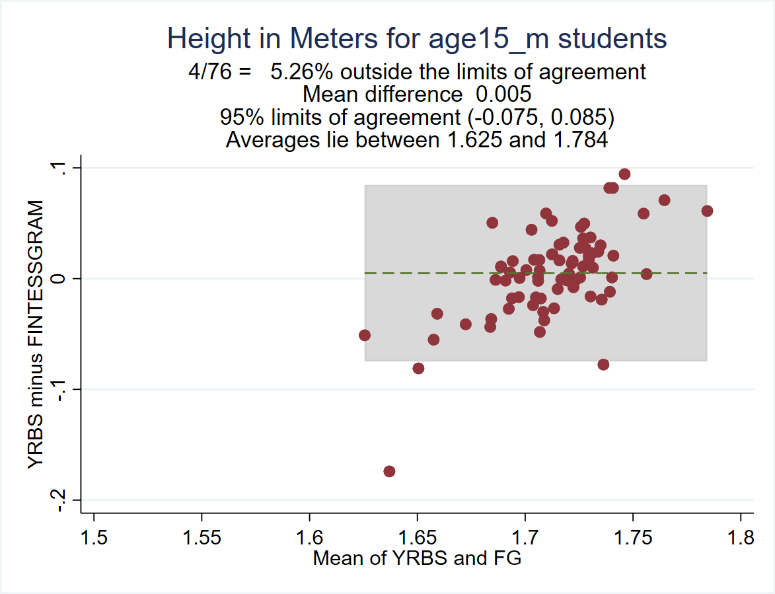

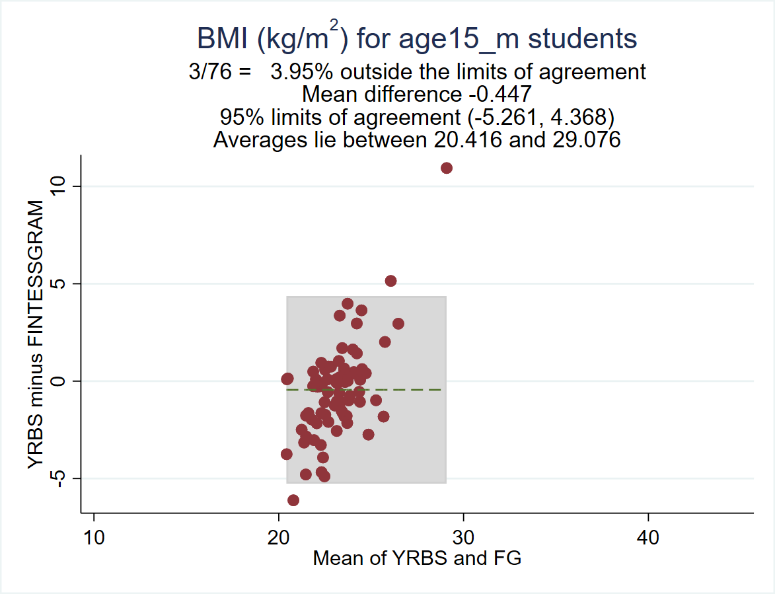

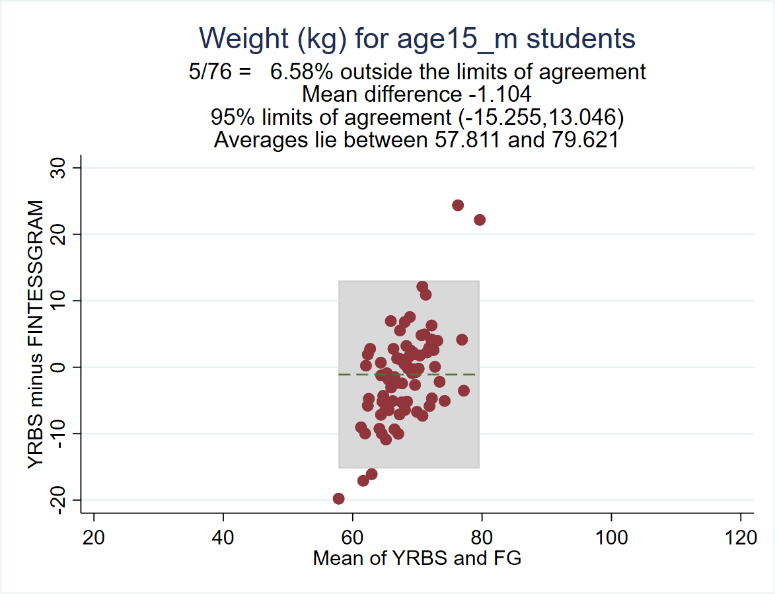


**Figure 20: Bland-Altman Plots of height, weight, and BMI, for Age 16 females and Age 16 males – all students**


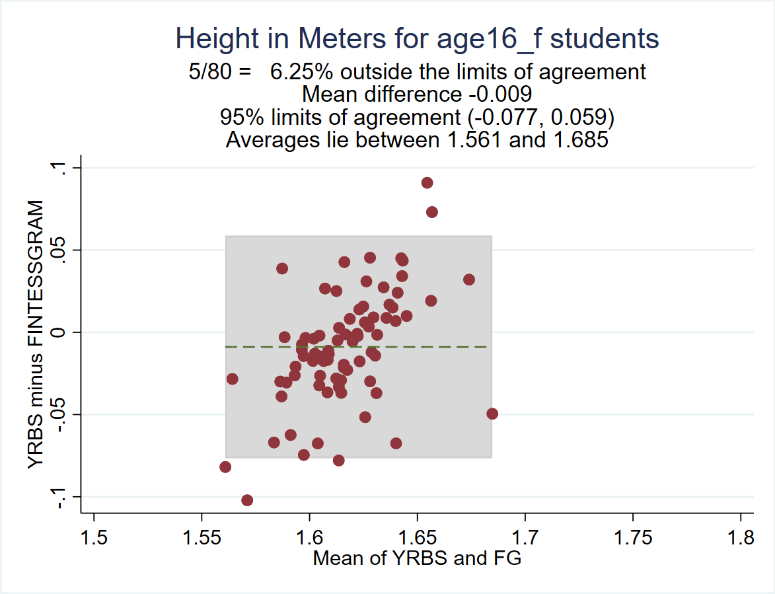

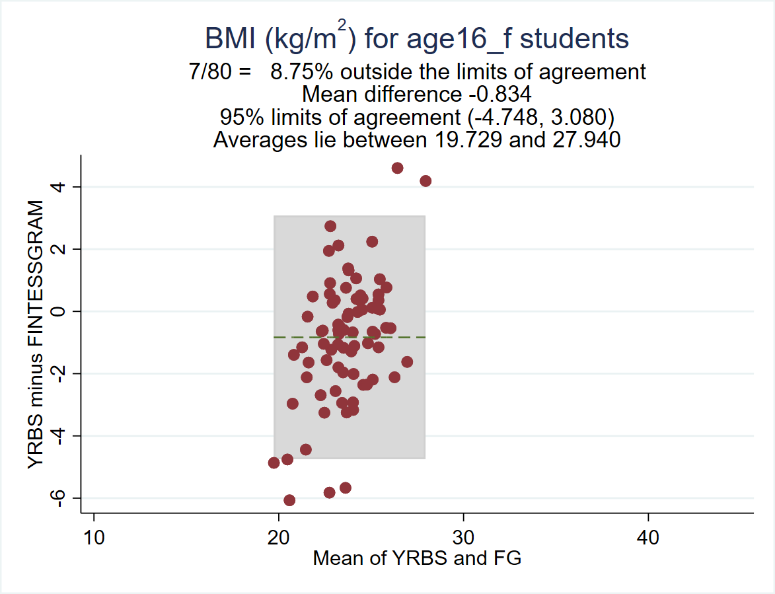

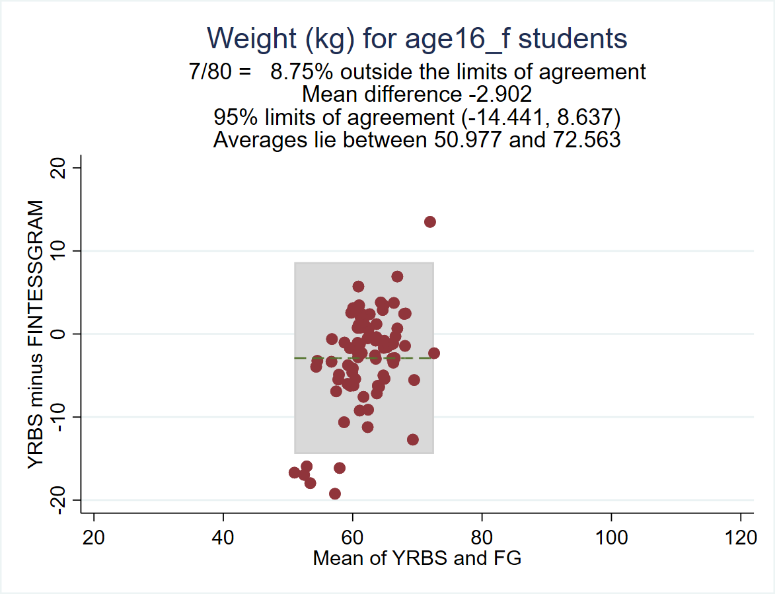

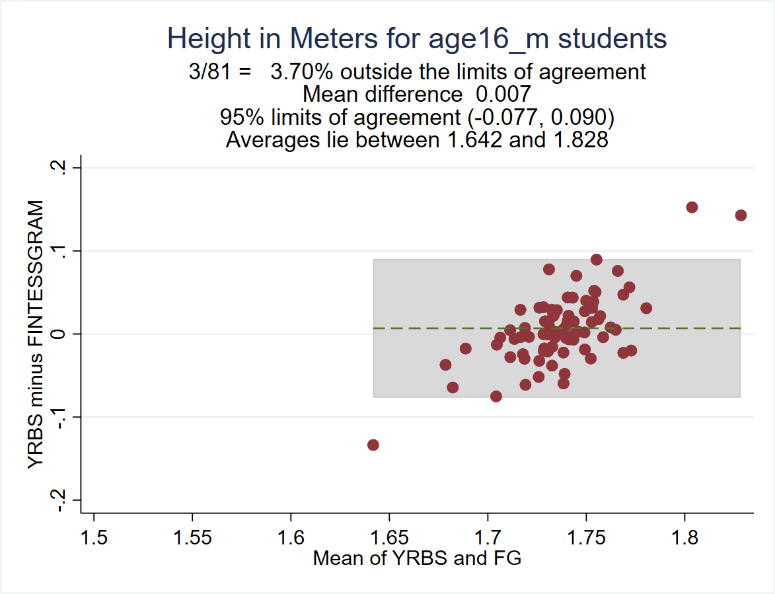

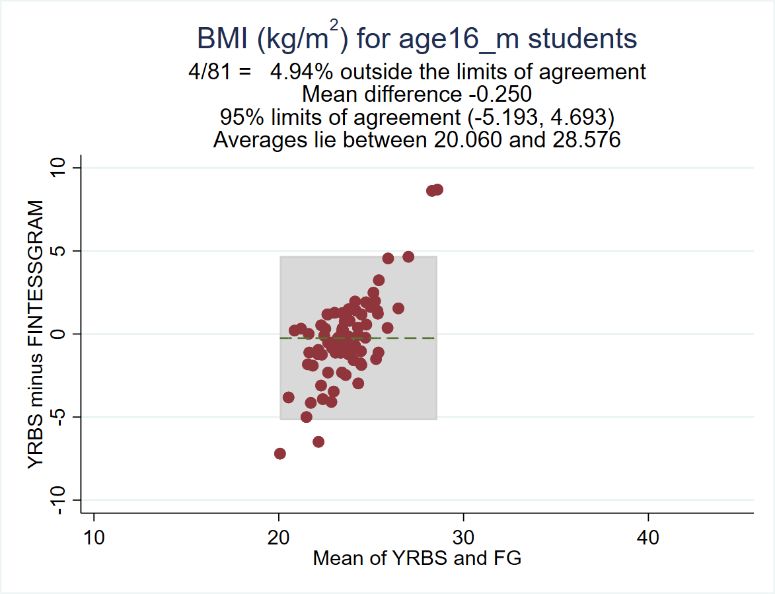

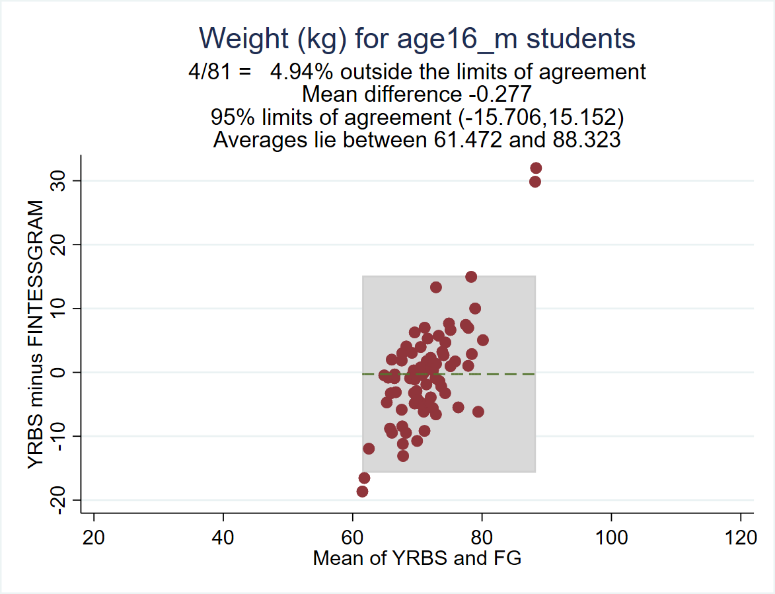


**Figure 21: Bland-Altman Plots of height, weight, and BMI, for Age 17 females and Age 17 males – all students**


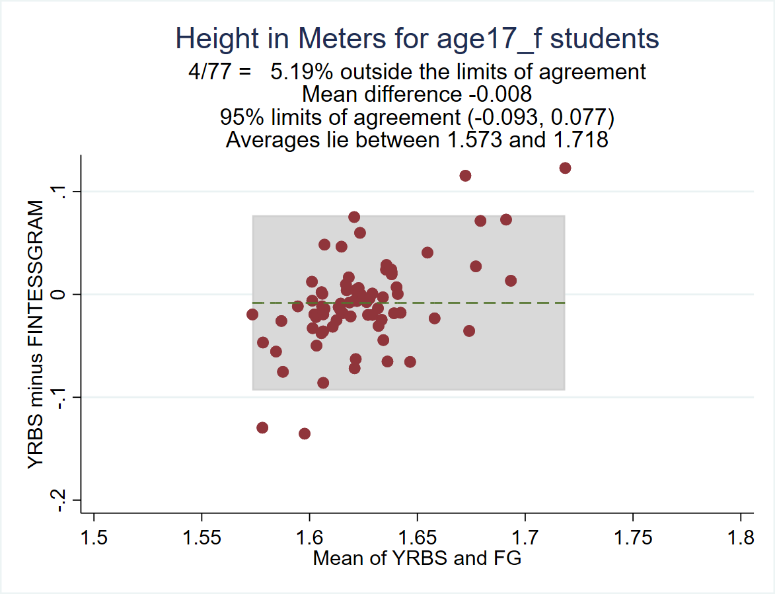

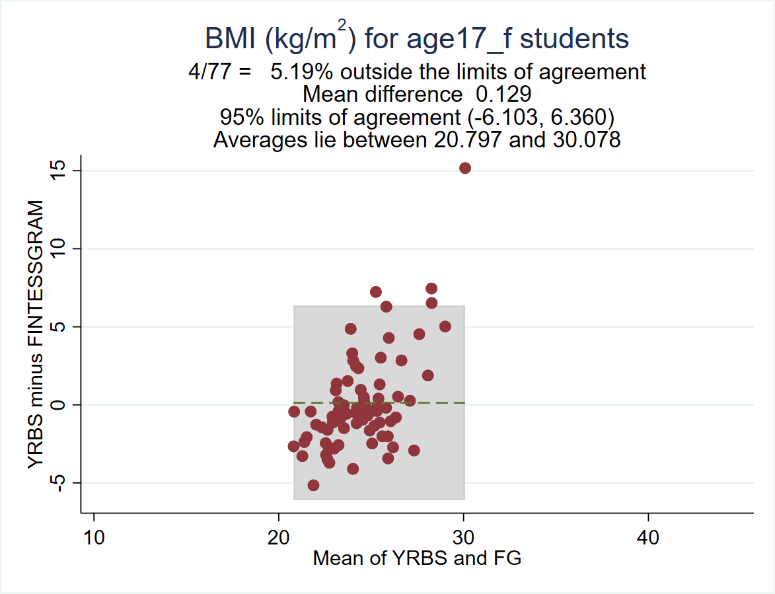

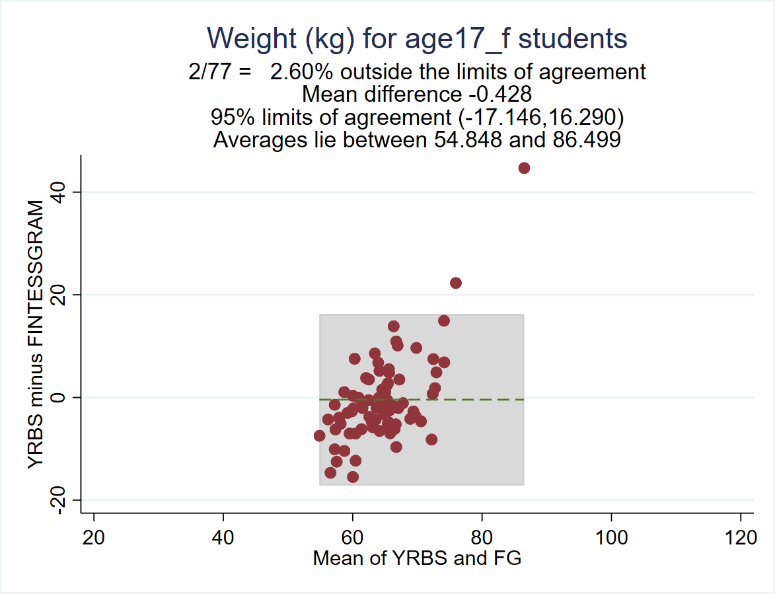

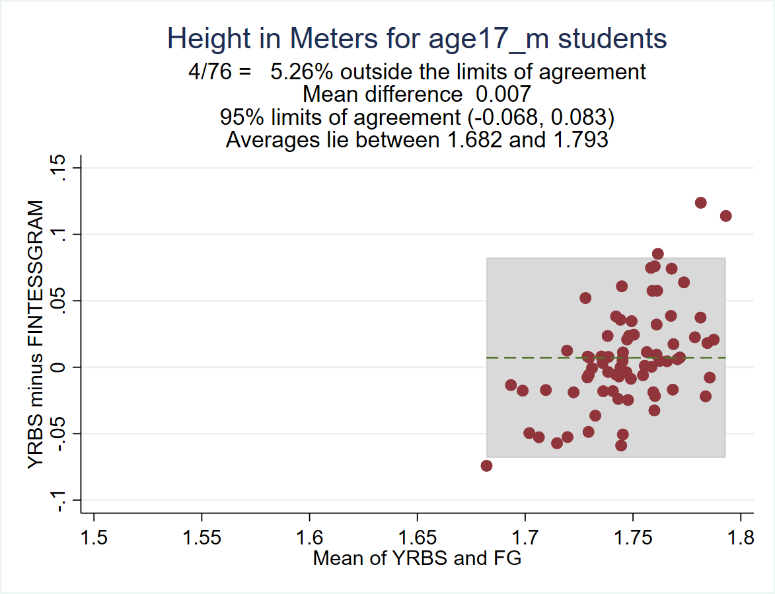

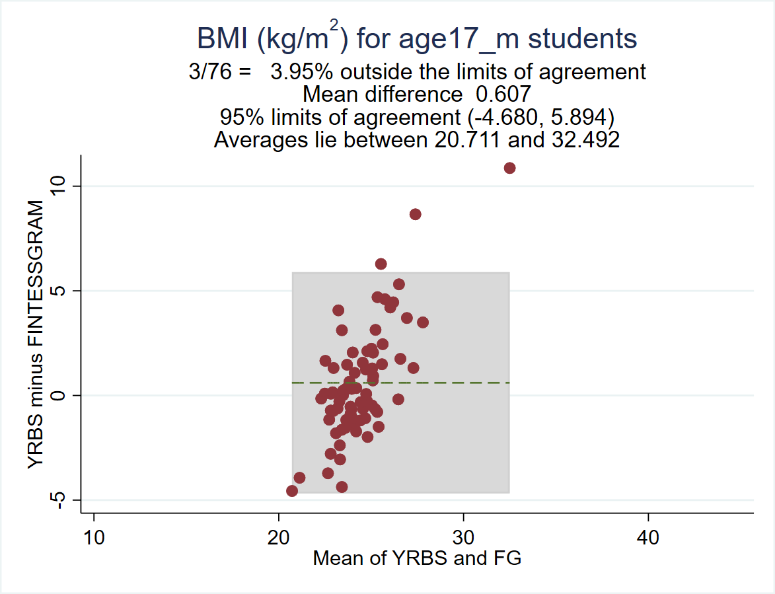

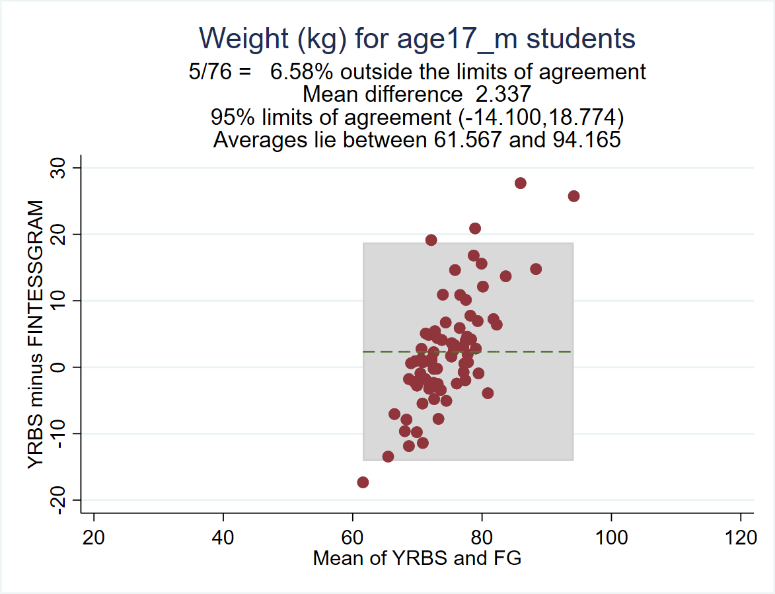


**Figure 22: Bland-Altman Plots of height, weight, and BMI, for Age 18 females and Age 18 males – all students**


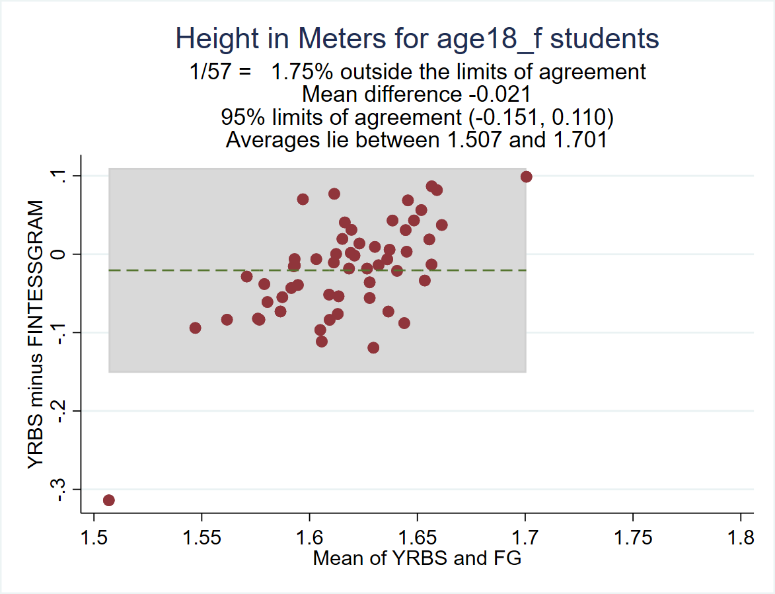

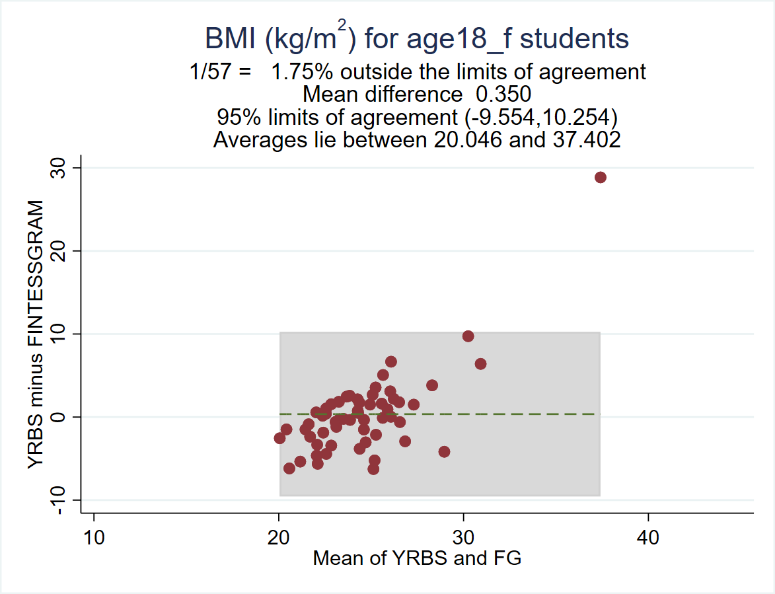

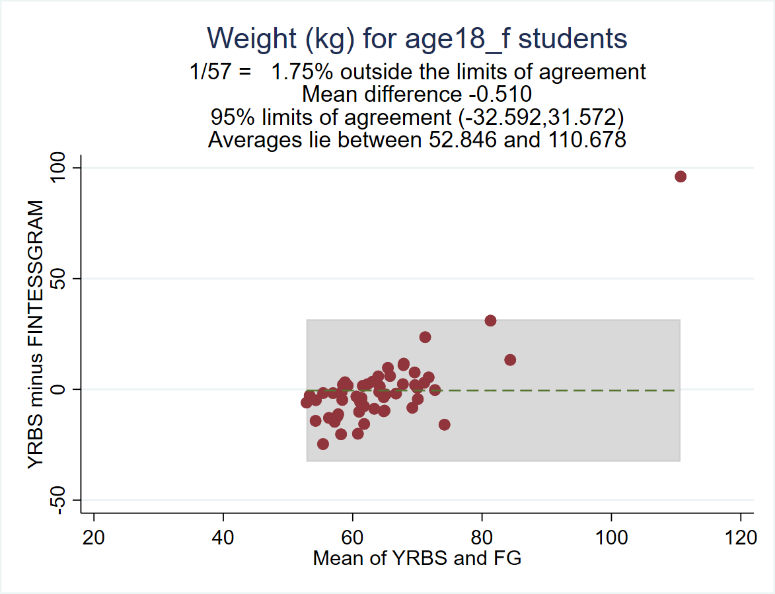

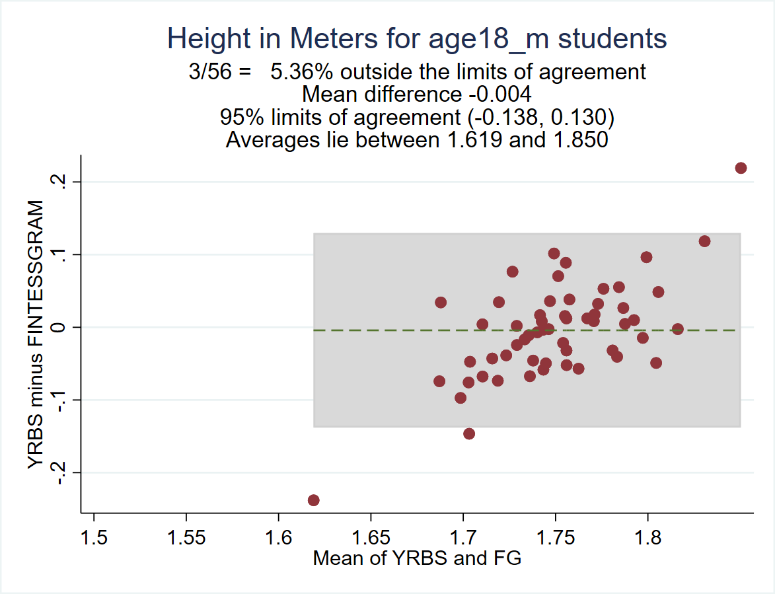

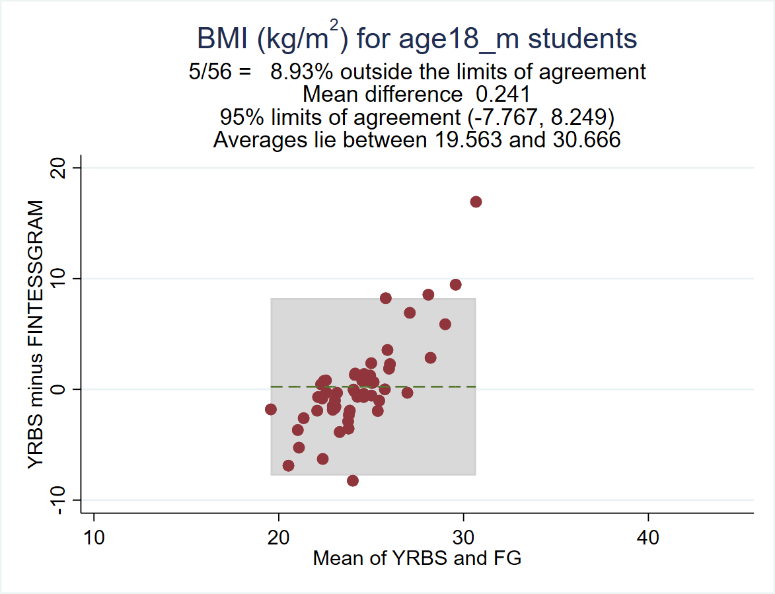

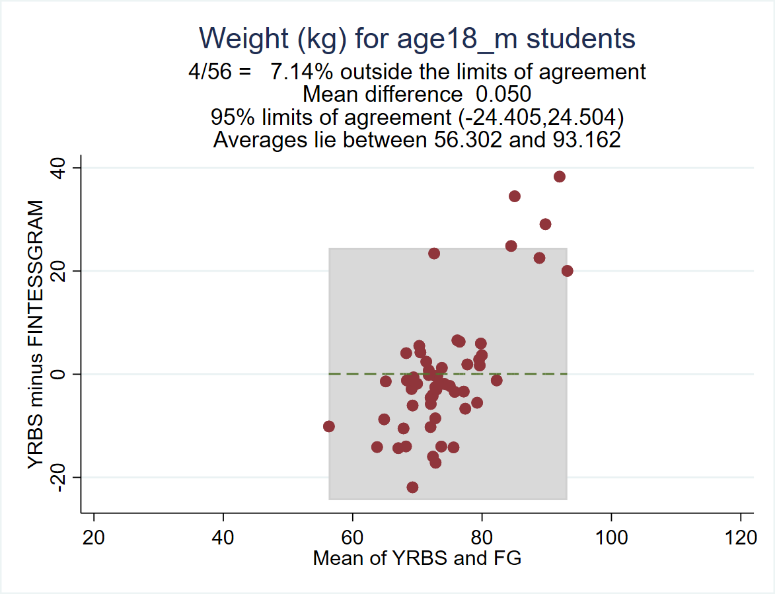


**Figure 23: Bland-Altman Plots of height, weight, and BMI, for Age 13 females and Age 13 males – students with obesity**


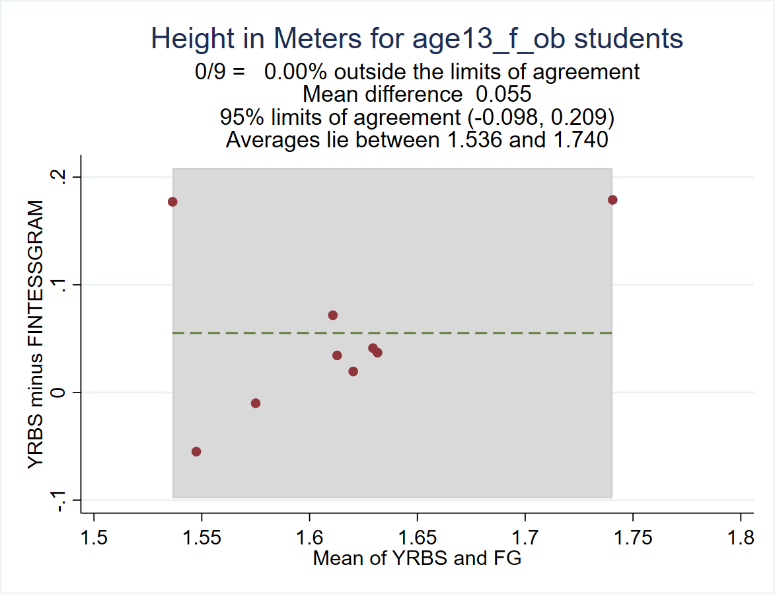

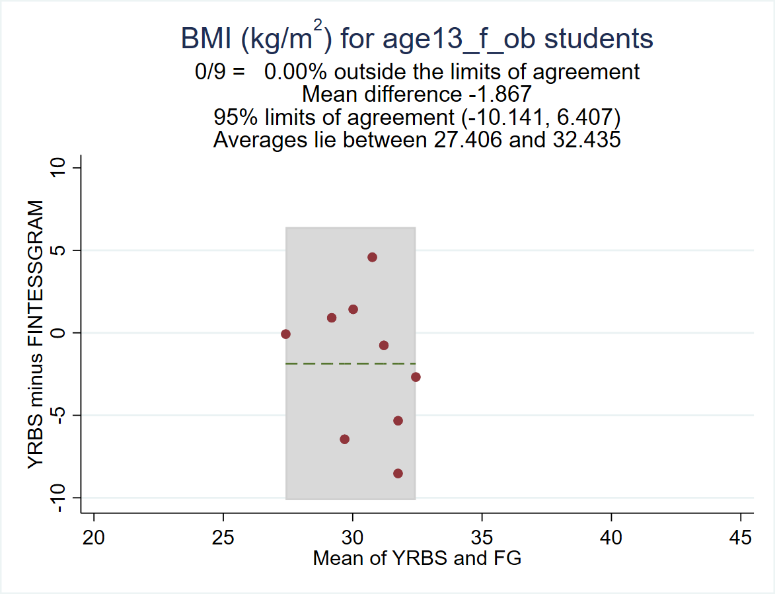

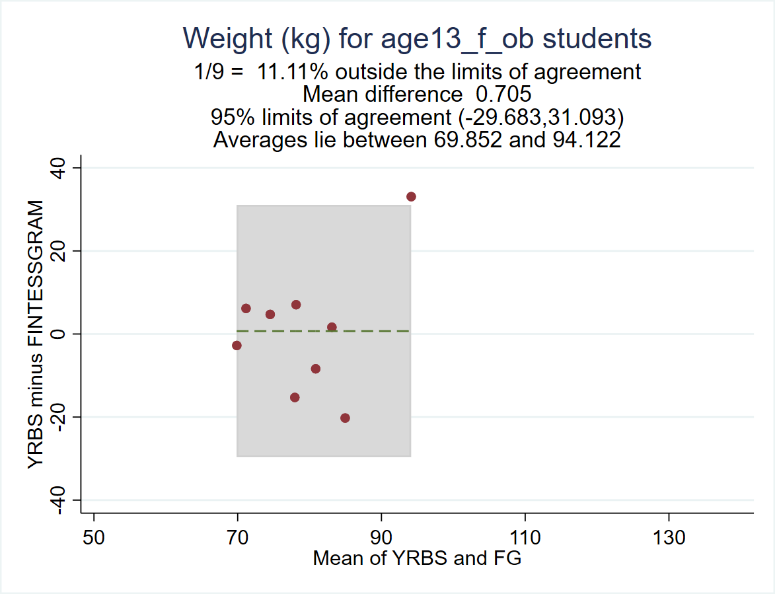

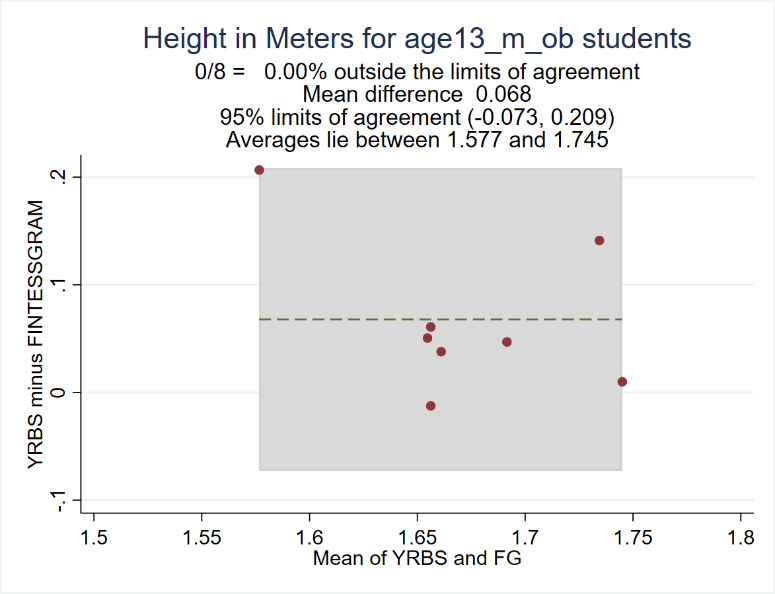

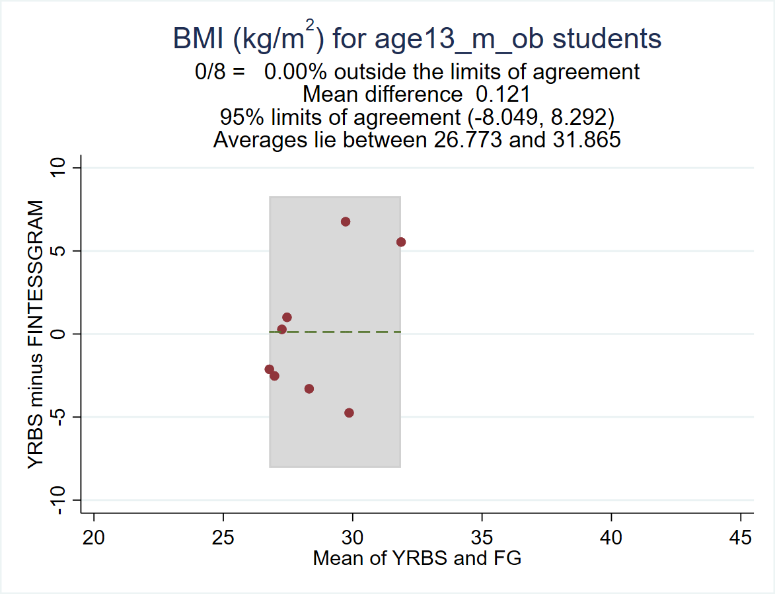

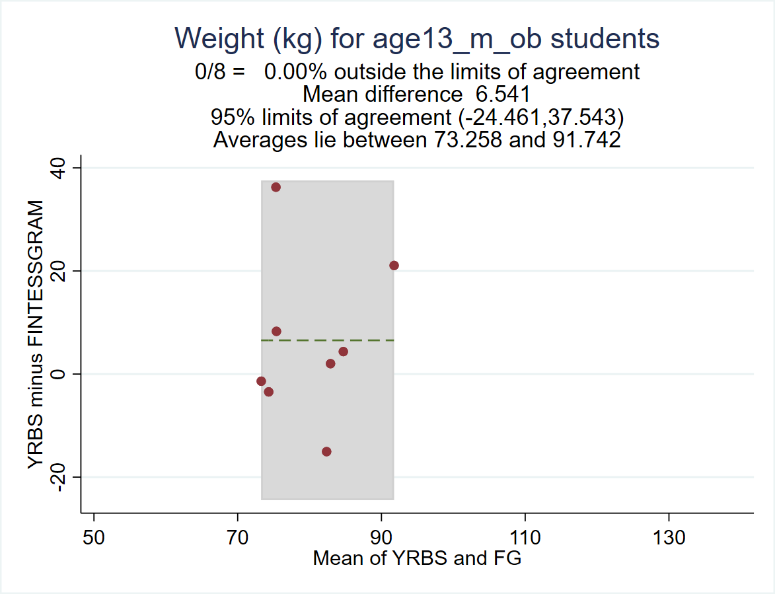


**Figure 24: Bland-Altman Plots of height, weight, and BMI, for Age 14 females and Age 14 males – students with obesity**


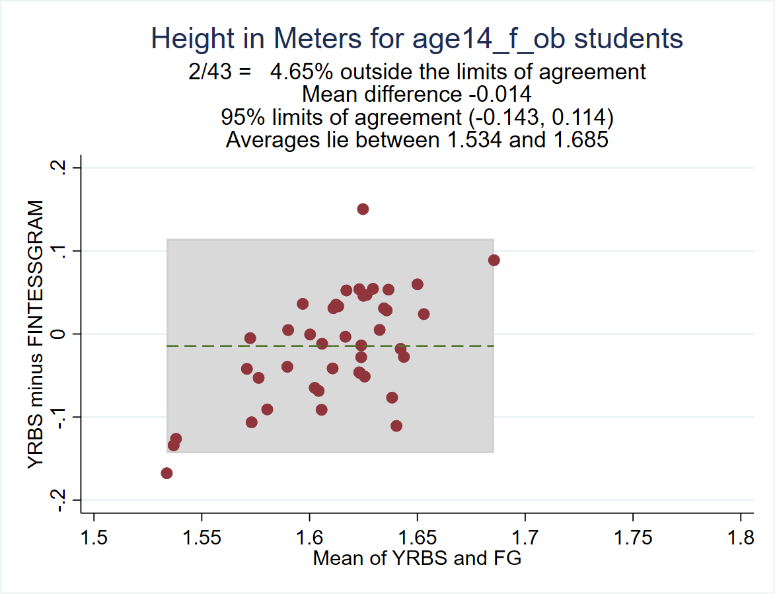

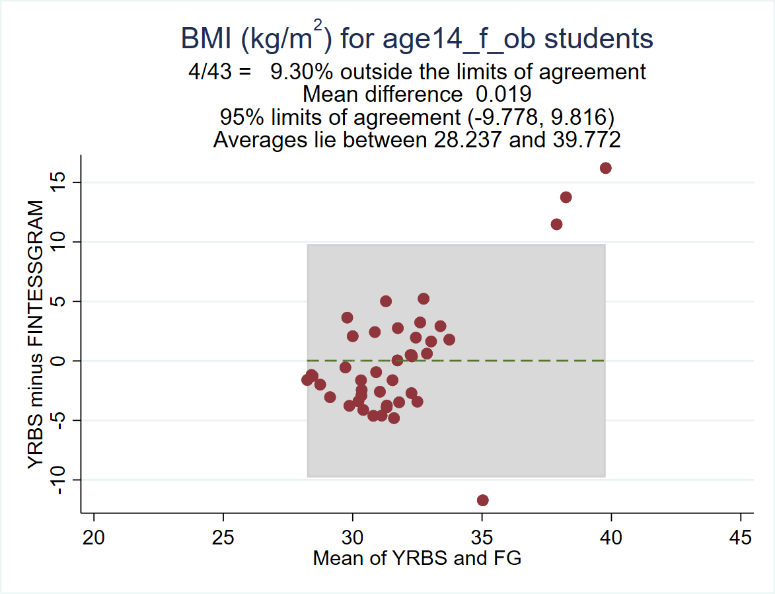

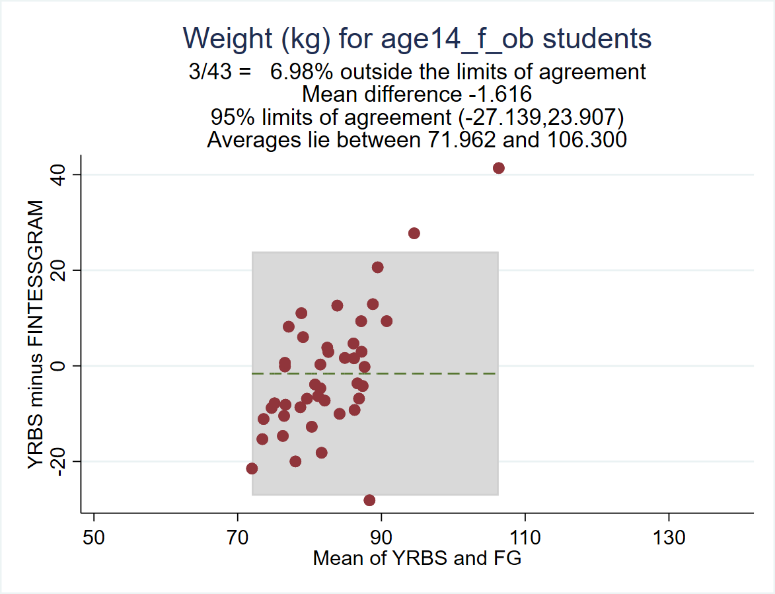

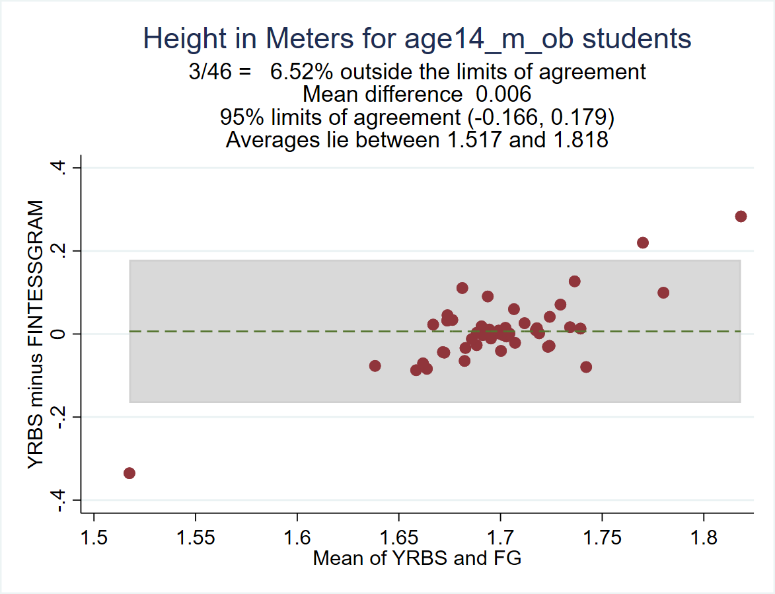

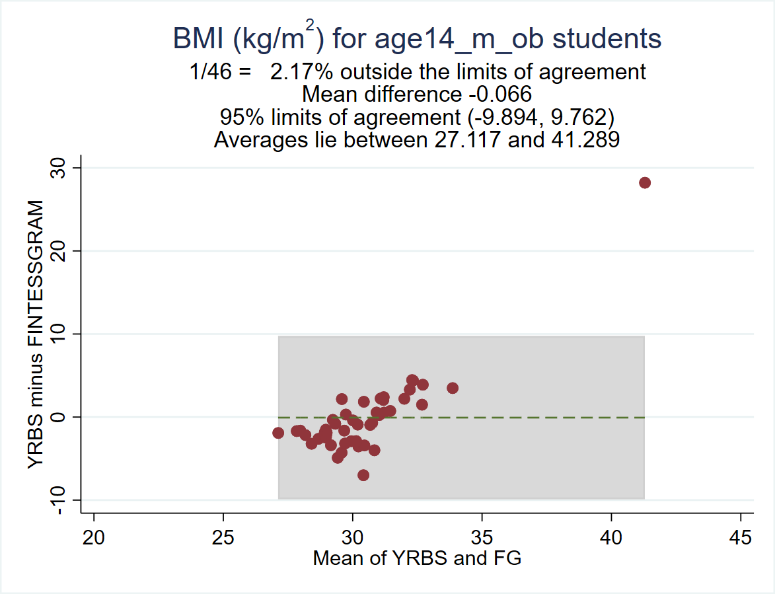

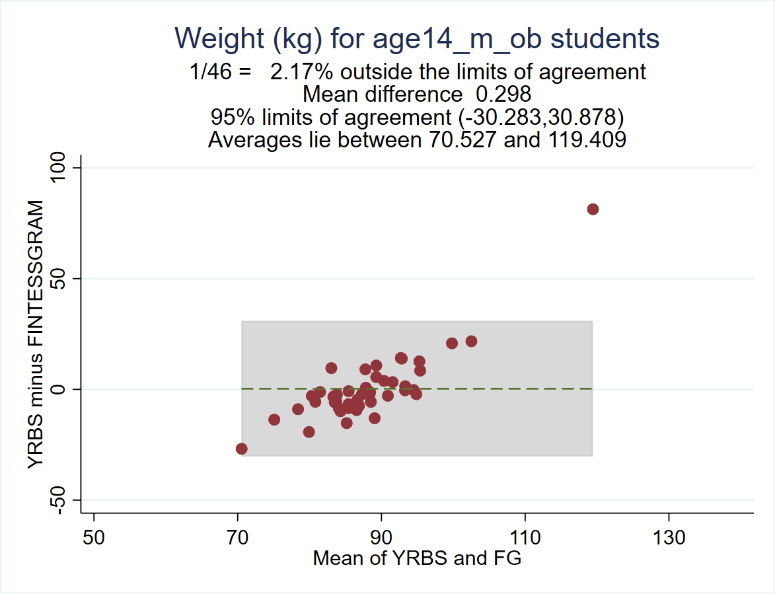


**Figure 25: Bland-Altman Plots of height, weight, and BMI, for Age 15 females and Age 15 males – students with obesity**


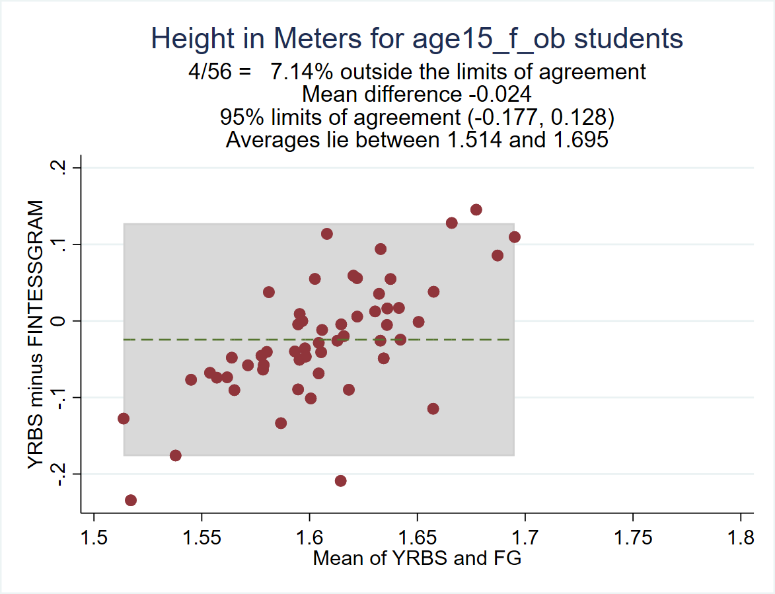

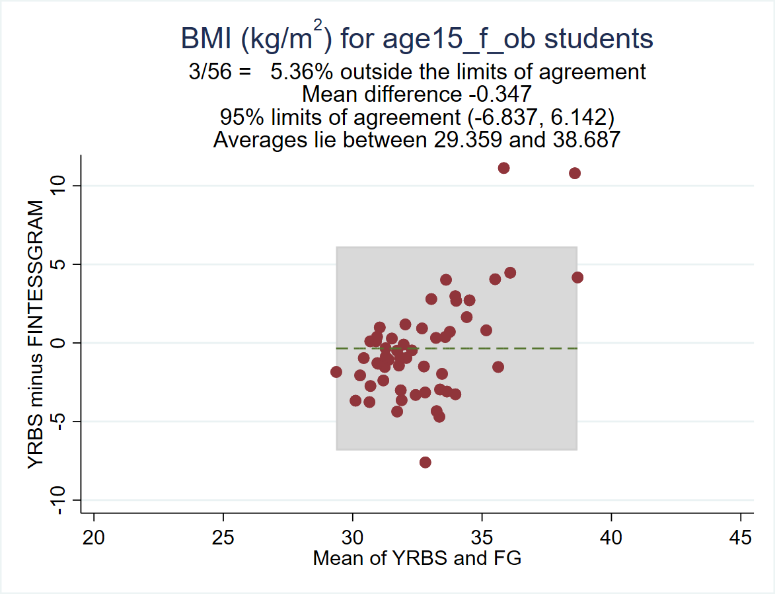

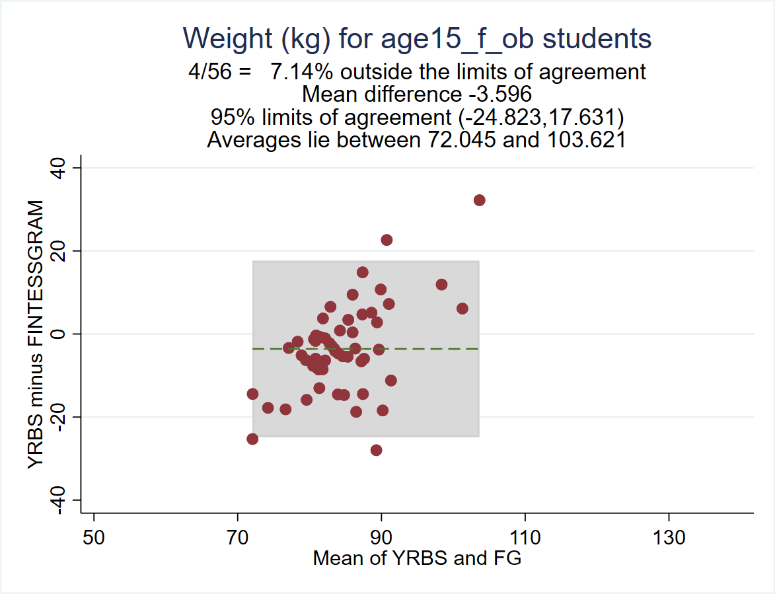

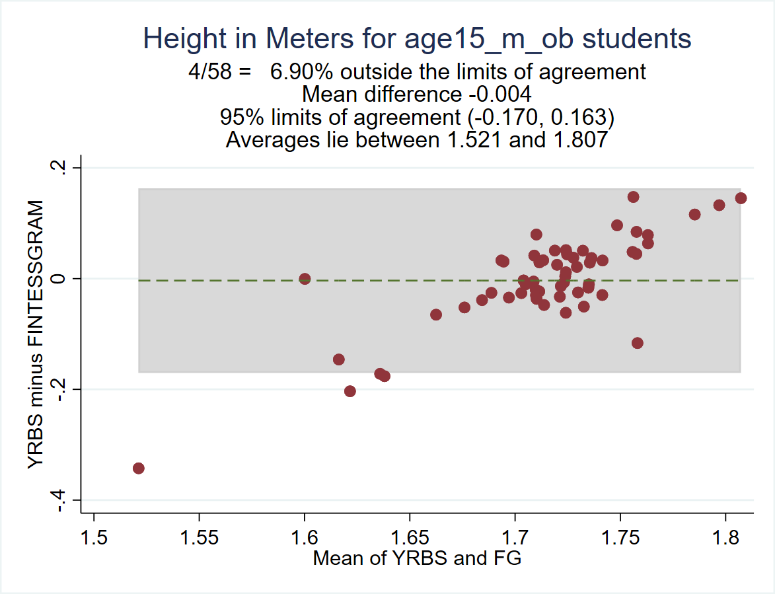


**Figure 26: Bland-Altman Plots of height, weight, and BMI, for Age 16 females and Age 16 males – students with obesity**

**Figure 27: Bland-Altman Plots of height, weight, and BMI, for Age 17 females and Age 17 males – students with obesity**

**Figure 28: Bland-Altman Plots of height, weight, and BMI, for Age 18 females and Age 18 males – students with obesity**

**Figure 29: Bland-Altman Plots of height, weight, and BMI, for Age 14 females and Age 14 males – students with severe obesity**

**Figure 30: Bland-Altman Plots of height, weight, and BMI, for Age 15 females and Age 15 males – students with severe obesity**

**Figure 31: Bland-Altman Plots of height, weight, and BMI, for Age 16 females and Age 16 males – students with severe obesity**

**Figure 32: Bland-Altman Plots of height, weight, and BMI, for Age 17 females and Age 17 males – students with severe obesity**

**Figure 33: Bland-Altman Plots of height, weight, and BMI, for Age 18 females and Age 18 males – students with severe obesity**
